# Willingness and capacity of publicly-funded vector control programs in the USA to engage in tick management

**DOI:** 10.1186/s13071-024-06400-8

**Published:** 2024-07-22

**Authors:** James C. Burtis, Erik Foster, Rebecca J. Eisen, Lars Eisen

**Affiliations:** Division of Vector-Borne Diseases, National Center for Emerging and Zoonotic Infectious Diseases, Centers for Disease Control and Prevention, Fort Collins, CO 80521 USA

**Keywords:** Tick control, Tick suppression, Tick surveillance, Tick bite prevention, Education, Mosquito control

## Abstract

**Background:**

The vast majority of vector-borne diseases in the USA are associated with mosquitoes or ticks. Mosquito control is often conducted as part of community programs run by publicly-funded entities. By contrast, tick control focuses primarily on individual residential properties and is implemented predominantly by homeowners and the private pest control firms they contract. We surveyed publicly-funded vector control programs (VCPs), presumed to focus mainly on mosquitoes, to determine what tick-related services they currently offer, and their interest in and capacity to expand existing services or provide new ones.

**Methods:**

We distributed a survey to VCPs in the Northeast, Upper Midwest and Pacific Coast states of the USA, where humans are at risk for bites by tick vectors (*Ixodes scapularis* or *Ixodes pacificus*) of agents causing Lyme disease and other tick-borne diseases. The data we report are based on responses from 118 VCPs engaged in vector control and with at least some activities focused on ticks.

**Results:**

Despite our survey targeting geographic regions where ticks and tick-borne diseases are persistent and increasing public health concerns, only 11% (12/114) of VCPs reported they took direct action to suppress ticks questing in the environment. The most common tick-related activities conducted by the VCPs were tick bite prevention education for the public (70%; 75/107 VCPs) and tick surveillance (48%; 56/116). When asked which services they would most likely include as part of a comprehensive tick management program, tick bite prevention education (90%; 96/107), tick surveillance (89%; 95/107) and tick suppression guidance for the public (74%; 79/107) were the most common services selected. Most VCPs were also willing to consider engaging in activities to suppress ticks on public lands (68%; 73/107), but few were willing to consider suppressing ticks on privately owned land such as residential properties (15%; 16/107). Across all potential tick-related services, funding was reported as the biggest obstacle to program expansion or development, followed by personnel.

**Conclusions:**

Considering the hesitancy of VCPs to provide tick suppression services on private properties and the high risk for tick bites in peridomestic settings, suppression of ticks on residential properties by private pest control operators will likely play an important role in the tick suppression landscape in the USA for the foreseeable future. Nevertheless, VCPs can assist in this effort by providing locally relevant guidelines to homeowners and private pest control firms regarding best practices for residential tick suppression efforts and associated efficacy evaluations. Publicly-funded VCPs are also well positioned to educate the public on personal tick bite prevention measures and to collect tick surveillance data that provide information on the risk of human encounters with ticks within their jurisdictions.

**Graphical Abstract:**

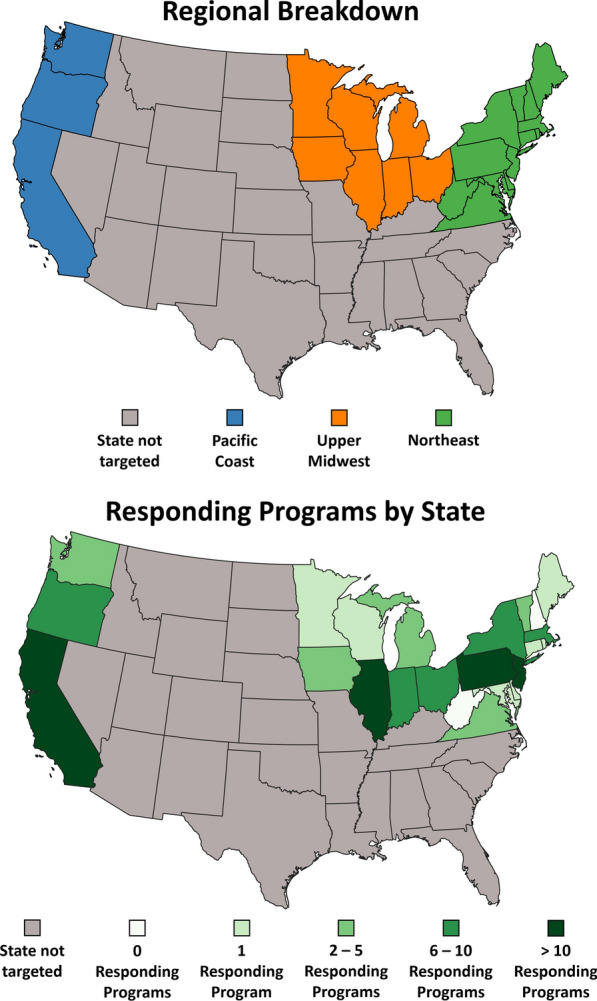

**Supplementary Information:**

The online version contains supplementary material available at 10.1186/s13071-024-06400-8.

## Background

The vast majority of vector-borne diseases in the USA are associated with either mosquitoes or ticks, with Lyme disease being the most common vector-borne disease in the conterminous USA [1]. *Ixodes scapularis* is the primary vector of causative agents of Lyme disease (*Borrelia burgdorferi* sensu stricto [*Borrelia burgdorferi* s.s.], *Borrelia mayonii*) and several other medically significant pathogens (*Anaplasma phagocytophilum*, *Babesia microti*, *Bo. miyamotoi* and Powassan virus) in the eastern USA, and *Ixodes pacificus* vectors some of these pathogens (*A. phagocytophilum*, *Bo. burgdorferi* s.s. and *Bo. miyamotoi*) on the West Coast [2, 3]. Public health efforts aiming to reduce bites by these and other tick species are predominantly educational, focusing on promotion of personal protective measures, such as using repellents, wearing appropriate clothing and avoiding tick habitat [4]. Unfortunately, these preventative measures are not used widely and consistently by the public [5, 6].

Another way to reduce the risk of human tick bites is to suppress tick populations in the environment. Suppression of ticks biting humans has primarily focused on individual residential properties, where biting ticks are commonly encountered [7–10]. In these settings, the homeowner and contracted private pest control operators are responsible for deployment of tick control measures [11]. Commercial vector control firms predominantly use the direct application of acaricides to suppress ticks on residential properties, with other suppression methods (e.g. interventions targeting rodents, or landscape management) used less commonly [12]. Tick suppression operations are rarely conducted by publicly-funded vector control programs (VCPs hereafter), whose primary focus is often on mosquito control. A National Association of County and City Health Officials (NACCHO) survey conducted in 2020 found that only 3% of responding local vector programs nationally were actively engaged in tick suppression activities, while 79% of agencies indicated they routinely engaged in mosquito control [13, 14]. Another national survey conducted by Mader et al. in 2018 reported a higher overall proportion (12%) of vector-borne disease professionals directly conducting or financially supporting tick suppression, with the majority of respondents involved in tick suppression being located in the Northeast [15]. However, these responses included tick suppression research activities, which are usually designed to investigate the efficacy of a suppression method and not deployed in a routine fashion, in addition to operational routine tick suppression.

Overall, there is very limited information about which tick suppression methods are used operationally by VCPs, or their interest in developing the capacity to provide new tick suppression services. These types of questions were addressed in a recent survey in New Jersey [16], but similar larger scale efforts covering key regions of the USA with a high burden of tick-borne diseases have been lacking. Tick suppression methods with the potential to be deployed at neighborhood or community level are still being explored, but questions remain about their cost [17] as well as their ability to reduce human tick bites [11, 18]. Most existing tick suppression methods are also difficult to scale up from individual properties to a neighborhood or community level in a cost-effective manner [17], further complicating the path to future large-scale deployment of tick management programs. Interest in deploying tick suppression may exist in some high-risk areas, such as New Jersey [16], but potential obstacles to the development and maintenance of tick management programs must be identified.

In the USA, mosquito control is often conducted as part of community programs run by publicly-funded entities; these programs typically include public education and mosquito surveillance components together with mosquito control [13]. Similarly, a comprehensive publicly-funded tick management program would likely include educational services and tick surveillance activities together with the deployment of various tick suppression methods. Apart from New Jersey [16], there is limited information on how commonly tick surveillance and educational services are provided by state or local VCPs, or whether these would be interested in expanding or developing such programmatic capacity.

We therefore conducted a survey of existing VCPs to determine which tick-related services they currently offer, and their interest in and capacity to expand existing services or provide new ones. Our survey focused on the Northeast, Upper Midwest and Pacific Coast states of the USA, where humans are at a relatively high risk for Lyme disease and other tick-borne diseases associated with bites by *Ixodes* ticks. It was designed to also identify potential obstacles VCPs face related to developing tick-related services. The primary objective of this survey was to determine which tick-related services are currently available and which services might be provided as part of a comprehensive tick management program if implemented by existing VCPs.

## Methods

### Target audience and survey distribution

The target audience for our survey comprised those working in VCPs who were involved in vector control activities, at least as part of their overall responsibilities, in the Northeast, Upper Midwest and Pacific Coast states of the USA. The organization of such VCPs varies among states and localities. Some VCPs are operated by entities solely dedicated to vector surveillance and control (e.g. mosquito abatement districts), whereas others represent one part of the activities conducted by local, county or state health or environmental departments. States in the Upper Midwest and Northeast were selected because they have high incidences of Lyme disease [19, 20]. Four lower incidence states in the Upper Midwest (Illinois, Indiana, Michigan, and Ohio) and three in the Pacific Coast region (California, Oregon, and Washington) were also included as ticks infected with *Bo. burgdorferi* s.s. and other pathogens transmitted by *I. scapularis* or *I. pacificus* have been detected in those states [21, 22]. This survey was focused on areas with high risk for exposure to *Ixodes*-borne pathogens, so did not include states where people are at risk mainly for pathogens transmitted by metastriate *Amblyomma* or *Dermacentor* ticks. All responding entities had to be directly involved in routine vector control activities at the state, county or local level to continue through the entire survey.

The survey was designed and distributed using REDCap (Research Electronic Data Capture) electronic data capture tools hosted at the Centers for Disease Control and Prevention (CDC) [23, 24]. Respondents were granted access to the survey using an open access REDCap link, which was distributed by email. An internal CDC list of VCPs was used to develop our initial list of contacts. Emails were sent directly to the managers, directors or entomologists at each identified VCP. We also utilized several regional professional listservs to further distribute our survey; specifically, listservs generated by the CDC-funded regional Centers of Excellence in Vector-Borne Diseases and local American Mosquito Control Association chapters. Recipients of our emails were also encouraged to share the survey link with others in their community of practice.

### Survey instrument and design

The survey contained a total of 170 questions, but was highly adaptive, so respondents did not view all questions. The full survey with logic as displayed in the survey codebook is provided in Supplemental file 1. The survey was divided into seven sections, each covering a different tick-related service with the exception of the first section, which asked demographic questions about the responding entities. The following five sections each focused on a different potential service that might be offered by a comprehensive tick management program: (i) provision of tick bite prevention education to the public; (ii) provision of tick suppression guidance to the public or private pest control firms; (iii) tick surveillance to assess risk of tick bites; (iv) efficacy assessment of tick suppression deployed by the public or private pest control firms; and (iv) suppression of tick populations. The section focused on the suppression of tick populations included specific questions related to four general categories of tick suppression methods: (i) acaricide application; (ii) rodent-targeted interventions; (iii) habitat and landscape management; and (4) deer-targeted interventions. In the final section of our survey, we asked respondents to choose the services they were most likely to offer as part of a comprehensive tick management program and estimate the approximate cost to offer each service.

The survey was opened for approximately 2 months (22 March 2023 through to 19 May 2023). The target of the survey was the VCP, rather than the individual respondent, so multiple individuals were allowed to take the survey as a group and record a single response so that their answers could be more comprehensive. Only one response for each VCP was used in the results reported here. If a VCP responded more than one time, we kept the most complete survey. If multiple survey responses from a single VCP were complete, then we kept the survey with the highest number of people participating in that response. If multiple responses from a VCP were recorded, each with one person participating, then the response from the highest-ranking respondent (e.g., director) was retained. Our results include only responses from entities which were actively engaged in routine vector control activities, with the goal of determining which tick-related activities this community is currently engaged in, as well as their interest in offering new tick-related services. Therefore, if an entity responded they were not currently engaged in vector control operations for public health purposes (Table 1), the survey ended, and the entity was not included in the reported results. If a respondent completed a section, their responses were included in the total for that section, even if they did not complete all seven sections of the survey. Risk and public concern for tick-borne disease can vary within states, so we wanted to ensure survey responses were focused on those agencies where ticks might be a feasible target and primary concern, as developing a tick control program in areas with limited tick-borne disease risk should not be a priority. At the end of each section of the survey, respondents were asked why they might be uninterested in providing that service. This question was asked eight times (Supplemental file 2), and those who responded more than 50% of the time that that ticks were not a concern in their jurisdiction were not included in the reported results.

**Table 1:** The demographic information of 118 responding vector control programs actively engaged in the control of vectors of human disease agents

Question	Response level	All regions combined	Northeast region	Upper Midwest region	Pacific Coast region
What is the jurisdictional level of your agency?	Regional	1% (1/118)	2% (1/55)	0% (0/33)	0% (0/30)
	State	7% (8/118)	15% (8/55)	0% (0/33)	0% (0/30)
	County	81% (95/118)	75% (41/55)	85% (28/33)	87% (26/30)
	City	6% (7/118)	9% (5/55)	6% (2/33)	0% (0/30)
	Other	6% (7/118)	0% (0/55)	9% (3/33)	13% (4/30)
Please indicate the size of the population that your program serves	0–49,999	11% (13/118)	7% (4/55)	9% (3/33)	20% (6/30)
	50,000–99,999	14% (16/118)	9% (5/55)	24% (8/33)	10% (3/30)
	100,000–249,999	19% (22/118)	18% (10/55)	27% (9/33)	10% (3/30)
	250,000–499,999	21% (25/118)	24% (13/55)	24% (8/33)	13% (4/30)
	500,000–999,999	20% (24/118)	31% (17/55)	6% (2/33)	17% (5/30)
	1,000,000+	15% (18/118)	11% (6/55)	9% (3/33)	30% (9/30)
Does your agency take direct action to control vector populations for public health purposes?	Yes	100% (118/118)	100% (55/55)	100% (33/33)	100% (30/30)
	No	0% (0/118)	0% (0/55)	0% (0/33)	0% (0/30)
What disease vectors or arthropod pests does your agency currently target?^a^	Ticks	18% (21/118)	18% (10/55)	12% (4/33)	23% (7/30)
	Mosquitoes	98% (115/118)	96% (53/55)	100% (33/33)	97% (29/30)
	Fleas	5% (6/118)	0% (0/55)	3% (1/33)	17% (5/30)
	Bed bugs	3% (4/118)	0% (0/55)	0% (0/33)	13% (4/30)
	Other^b^	11% (13/118)	5% (3/55)	9% (3/33)	23% (7/30)

^a^Respondents could select multiple options for this question. This question is also logic dependent on the previous question, which asked specifically about control of vector populations

^b^Common ‘Other’ responses included, greenhead flies, black flies, rodents and yellowjackets. All ‘Other’ responses also selected at least one of the four other options for this question

### Statistical analyses

We calculated the proportion of VCPs across regions that are currently engaged or interested in engaging in tick surveillance activities, educational services and tick suppression.

Chi-squared (contingency table) tests were used to assess the significance of regional differences in proportional responses for the questions which directly measured what activities were being offered and the interest of VCPs to expand these activities or initiate new ones. No analyses were conducted for VCPs currently deploying tick control as we did not have sufficient responses for an analysis. Chi-squared tests were also used to assess regional differences in the proportions of VCPs that believed the community in their jurisdiction would support a tax increase for tick-related services. A total of 22 Chi-squared tests were conducted, and all *P*-values were corrected for multiple comparisons with the Benjamini and Hochberg method [25]. All analyses were conducted in R version 4.2.1 [26].

## Results

### Response rate and VCP demographics

**Fig. 1:**
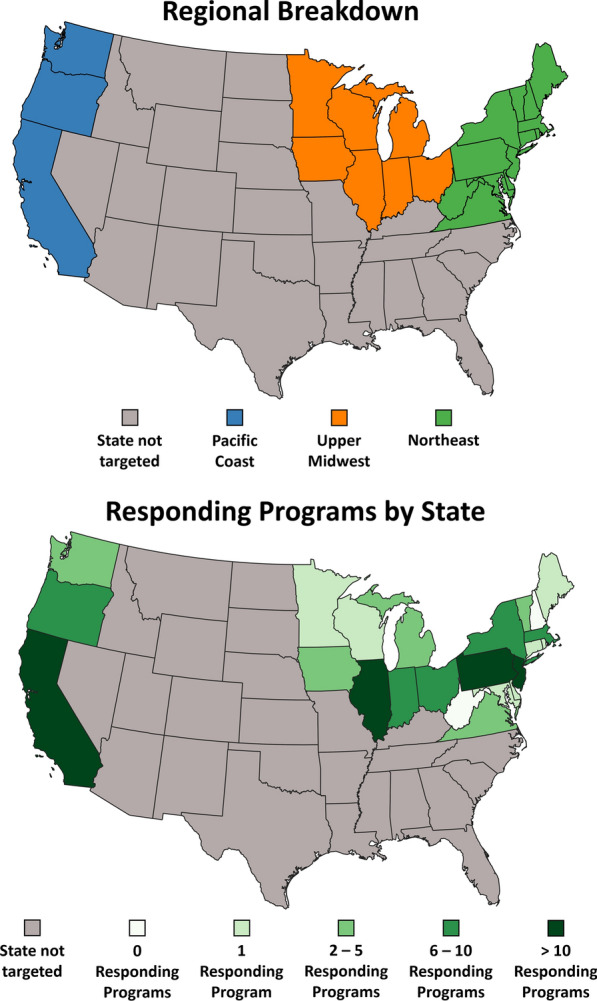
Map of the United States showing the states included in each of the three regions (top) and the number of responding vector control programs from each state targeted in our survey (bottom)

Our initial contact list included 382 VCPs, located across 23 states in the three targeted regions (see Fig. 1 for a map showing these states and regions). A total of 196 entities completed at least one section of the survey. Of these responding entities, 40 indicated they did not conduct any type of vector control operations, and the survey ended for these entities at the section “Introduction.” These entities did not continue through the other survey sections and were excluded from the final count of responding VCPs. Another 26 responses were removed because they were repeat responses from the same entities. Finally, 12 responses were excluded because they consistently indicated that ticks were not a concern in their jurisdiction. Of the 12 entities who did not consider ticks to be a concern, seven were from the Central Valley of California and southern California where acarological risk is low, two were in Oregon and one each were from the states of Washington, Michigan and Virginia. A summary of these excluded responses can be found in Supplemental file 3. The final number of unique responding VCPs which were actively engaged in vector control operations was 118. The majority of these 118 VCPs were either dedicated solely to vectors (47%, *N* = 55) or public health departments (31%, *N* = 36); the remaining VCPs were split between public works departments (8%, *N* = 10), environmental health agencies (8%, *N* = 10) and others (6%, *N* = 7) that did not fit into these categories (e.g. animal control). Most of the 118 VCPs were organized at the county-level (81%, *N* = 95) and served populations of > 50,000 people (89%, *N* = 105). The vast majority (98%, *N* = 115) of the VCPs considered mosquitoes to be an important target vector for their control operations (Table 1). Only 18% (*N* = 21) of agencies responded that they considered ticks to be an important target for their operations. This question was logically conditional on the preceding one, which asked about vector control operations, but did not explicitly mention control or suppression. Therefore, some respondents may have interpreted this question to be asking about broader tick-related activities and this percentage may be an overestimate for tick suppression operations. A more conservative value is shown in the section Tick suppression activities.

Across the three regions, the Northeast contributed the most VCP responses with 55 responses, followed by the Midwest with 33 responses and the Pacific Coast with 30 responses (Fig. 1). We received a response from at least one VCP in each state, with the exception of New Hampshire and West Virginia from which no responses were received (Fig. 1). Three states (California, New Jersey and Pennsylvania) each provided > 10 unique VCP responses. All seven sections of the survey were completed by 107 (91%) of the 118 responding VCPs. VCPs that completed a section were included in the total for that section, even if they did not complete all seven sections of the survey. Hence, the denominator (i.e. total number of responding VCPs) varies between the different sections of the survey described below.

### Tick surveillance activities

The data presented in the following sections are for tick surveillance for all regions combined because there were no significant differences between regions for any of the survey questions (see Table 2). Nearly half of responding VCPs (48%, 56/116 of responding VCPs) reported they conducted tick surveillance to assess the local risk of tick bites. The overwhelming majority (98%, 55/56) of those responding VCPs used tick dragging or flagging methods to collect these data. The most common use of these data was to inform the public of high-risk areas for exposure to ticks (61%, 34/56) and to map the local tick exposure risk (57%, 32/56). Few VCPs (9%, 5/56) used surveillance data to direct or evaluate their own tick suppression efforts.

**Table 2:** The proportion of responding vector control programs that are currently conducting tick surveillance activities and their interest in expanding those services, and the interest of vector control programs not conducting tick surveillance to develop this capacity

Question	Response	All regions combined	Northeast region	Upper Midwest region	Pacific Coast region	Chi-squared P-value^a^
*Tick surveillance*						
Does your agency directly conduct tick surveillance (e.g. tick dragging/wildlife collections/veterinary submission/public submissions) to measure the risk of tick exposure in your jurisdiction?	Yes	48% (56/116)	56% (30/54)	39% (13/33)	45% (13/29)	N.S *P* = 0.557
	No	52% (60/116)	44% (24 /54)	61% (20/33)	55% (16/29)	
Do you want to expand your agency's capacity to use or conduct tick surveillance to measure the risk of tick exposure in your jurisdiction?	Yes	82% (46/56)	77% (23/30)	92% (12/13)	85% (11/13)	N.S *P* = 0.636
	No	18% (10/56)	23% (7/30)	8% (1/13)	15% (2/13)	
If resources, including funding and training opportunities, were available, would your agency be interested in developing the capacity to inform the public of high-risk areas for exposure to ticks and tickborne disease agents?	Yes	83% (50/60)	79% (19/24)	90% (18/20)	81% (13/16)	N.S *P* = 0.727
	No	17% (10/60)	21% (5/24)	10% (2/20)	19% (3/16)	
*Tick control efficacy assessment*^b^						
Does your agency assess the efficacy of tick suppression deployed by pest control firms or property owners on public or private property?	Yes	0% (0/107)	0% (0/51)	0% (0/31)	0% (0/25)	N.A
	No	100% (107/107)	100% (51/51)	100% (31/31)	100% (25/25)	
If resources, including funding and training opportunities, were available, would your agency be interested in assessing tick suppression efficacy?	Yes	45% (48/107)	49% (25/51)	28% (9/31)	56% (14/25)	N.S *P* = 0.221
	No	55% (59/107)	51% (26/51)	72% (22/31)	44% (11/25)	

*N.A.* Not applicable, *N.S.* non-significant

^a^Chi-squared *P*-values correspond to tests comparing responses between the three regions, corrected for multiple comparisons using the Benjamini–Hochberg method

^b^No program offered this service, so interest in expanding an existing program could not be determined

Of those VCPs already conducting tick surveillance activities to assess tick-bite risk, 82% (46/56 of responding VCPs) were interested in expanding their capacity to do so. Of those VCPs who did not conduct tick surveillance activities, 83% (50/60) indicated they would be interested in starting to do so in the future if resources and training were provided. None of the 107 responding VCPs currently used tick surveillance to assess the efficacy of tick suppression deployed by the public or private pest control firms. Since no VCP was currently engaged in this activity, we could not measure interest in expanding existing capacity. Of the responding VCPs who did not offer this service, 45% (48/107) were interested in developing the capacity to do so if resources were available (Table 2).

### Educational services

The data presented here for educational services are for all regions combined, as there were no significant differences between regions for any of the survey questions (see Table 3). Guidance for the deployment of tick suppression methods by the public or private pest control firms was provided by 40% (43/107 of responding VCPs) of responding VCPs. The most common tick suppression method for which guidance was provided was habitat or landscape management (84%, 36/43 of responding VCPs), followed by acaricide application (37%, 16/43). Responding VCPs used a variety of methods to distribute this information, including presentations at public events (84%, 36/43), websites (79%, 34/43), print materials (79%, 34/43) and social media (65%, 28/43). Of those responding VCPs already providing tick suppression guidance, 79% (34/43) were interested in expanding their current services. Of those VCPs not currently providing this service, 66% (42/64) were interested in developing the capacity to do so if resources were provided (Table 3).

**Table 3:** The proportion of responding vector control programs that are currently offering tick-related educational services and their interest in expanding those services

Question	Response	All regions combined	Northeast region	Upper Midwest region	Pacific Coast region	Chi-squared *P*-value^a^
*Tick suppression guidance*						
Does your agency provide guidance to deploy tick suppression on private, residential properties through educational materials or site visits in your jurisdiction?	Yes	40% (43/107)	45% (23/51)	42% (13/31)	28% (7/25)	N.S *P* = 0.557
	No	60% (64/107)	55% (28/51)	58% (18/31)	72% (18/25)	
Do you want to expand your agency's capacity to provide tick suppression guidance to residential property owners?	Yes	79% (34/43)	78% (18/23)	92% (12/13)	57% (4/7)	N.S *P* = 0.214
	No	21% (9/43)	22% (5/23)	8% (1/13)	43% (3/7)	
If resources, including funding and training opportunities, were available, would your agency be interested in providing tick suppression guidance to residential property owners?	Yes	66% (42/64)	64% (18/28)	61% (11/18)	72% (13/18)	N.S *P* = 0.788
	No	34% (22/64)	36% (10/28)	39% (7/18)	28% (5/18)	
*Educational materials on tick bite prevention*						
Does your agency create their own educational materials (e.g. website/print/social media/public event displays) regarding personal tick bite prevention?	Yes	49% (52/107)	53% (27/51)	52% (16/31)	36% (9/25)	N.S *P* = 0.557
	No	51% (55/107)	47% (24/51)	48% (15/31)	64% (16/25)	
Does your agency work with a group or link to a group's information for education regarding personal tick bite prevention?	Yes	58% (62/107)	67% (34/51)	62% (19/31)	36% (9/25)	N.S *P* = 0.137
	No	42% (45 /107)	33% (17/51)	39% (12/31)	64% (16/25)	
Do you want to expand your agency's capacity to educate the public about tick-bite prevention?	Yes	81% (42/52)	85% (23/27)	75% (12/16)	78% (7/9)	N.S p = 0.788
	No	19% (10/52)	15% (4/27)	25% (4/16)	22% (2/9)	
If resources, including funding and training opportunities were available, would your agency be interested in developing the capacity to educate the public about personal tick-bite prevention?	Yes	78% (43/55)	79% (19/24)	60% (9/15)	94% (15/16)	N.S *P* = 0.214
	No	22% (12/55)	21% (5/24)	40% (6/15)	6% (1/16)	

The interest of vector control programs to start offering new educational services is also shown

*N.S.* Non-significant

^a^Chi-squared *P*-values correspond to tests comparing responses between the three regions, corrected for multiple comparisons using the Benjamini–Hochberg method

Some form of tick bite prevention educational services were provided by 70% (75/107) of responding VCPs. As noted in Table 3, this could include providing materials developed by their own program (49%, 52/107 of responding VCPs) or by linking to another organization’s information (58%, 62/107). For VCPs developing their own educational materials, they were mostly distributed to the public using physical printed materials (84%, 44/52 of responding VCPs) and social media (77%, 40/52). VCPs linking to outside tick bite prevention educational resources most commonly linked to sources from local and state health departments (84%, 52/62 of responding VCPs) or the CDC (77%, 48/62). Of the responding VCPs that develop their own educational materials, 81% (42/52) would be interested in expanding these services. Fully 78% (43/55) of responding VCPs which do not currently develop their own educational materials for tick bite prevention were interested in doing so if resources were provided (Table 3).

### Tick suppression activities

The majority of responding VCPs across regions (87%, 99/114 of responding VCPs) indicated they did not take direct action to suppress ticks in their jurisdictions. Three responding VCPs indicated they deployed only ‘other’ methods of tick suppression. Reviewing written responses, these ‘other’ methods referred to tick dragging or flagging activities, which would be classified as surveillance rather than suppression; therefore, these VCPs were considered to be the same as the ‘never’ category with regards to engaging in tick suppression. Thus, 89% (102/114) of responding VCPs did not deploy tick suppression. For those few VCPs who did engage in tick suppression activities (11%, 12/114), acaricide application was by far the most commonly used method (92%, 11/12). Tick suppression activities were most common in VCPs responding from states in the Northeast, with 19% (10/53) of VCPs responding they deploy tick control at least once per year. Only 7% (2/28) of responding VCPs in the Pacific Coast states conducted tick suppression activities and no VCP did so in the Upper Midwest (Table 4).

**Table 4:** The proportion of responding vector control programs currently engaged in tick suppression and the different methods of tick population suppression used

Question	Response	All regions combined	Northeast region	Upper Midwest region	Pacific Coast region
On average, how often does your agency use tick suppression methods?	Never	89% (102/114)	81% (43/53)	100% (33/33)	93% (26/28)
	Once a year	1% (1/114)	2% (1/53)	0% (0/33)	0% (0/28)
	Multiple times per year	5% (6/114)	11% (6/53)	0% (0/33)	0% (0/28)
	Other^b^	4% (5/114)	6% (3/53)	0% (0/33)	7% (2/28)
What, if any, tick suppression methods does your agency currently deploy?^a^	Application of acaricides to vegetation	92% (11/12)	90% (9/10)	0% (0)	100% (2/2)
	Deer-targeted intervention	17% (2/12)	20% (2/10)	0% (0)	0% (0/2)
	Rodent-targeted intervention	8% (1/12)	10% (1/10)	0% (0)	0% (0/2)
	Habitat or landscape management	25% (3/12)	10% (1/10)	0% (0)	100% (2/2)
	Other^b^	8% (1/12)	0% (0/10)	0% (0)	50% (1/2)

^a^Indicates respondents could select multiple options for this question

^b^‘Other’ responses included on demand services requested by another agency or otherwise intermittently, and services that were previously offered and discontinued

Since current tick suppression activities were uncommon, we focused on interest in offering new tick suppression services across four general methodological categories: acaricide application, rodent-targeted interventions, habitat and landscape management, and deer-targeted interventions (see also Table 5). Across all regions, acaricide application was the most commonly selected method that responding VCPs indicated they would consider providing (71%, 73/103 of responding VCPs) if resources were available, followed by rodent-targeted interventions (58%, 64/110) and habitat and landscape management (50%, 53/107). Deer-targeted interventions were the least likely methods VCPs would consider offering (43%, 46/107), though notably over 40% of VCPs would consider this approach (Table 5). Interest in providing tick suppression services on private properties was limited, with between 26 and 33% of VCPs responding they would deploy various tick suppression methods on private property. However, interest in treating private properties was significantly higher in the Pacific Coast region (64–73% of positive responses across methods) compared with all other regions (Table 5).

**Table 5:** The interest of vector control programs regarding the development of tick suppression services using different methods

Question	Response	All regions combined	Northeast	Upper Midwest	Pacific Coast	Chi-squared *P*-value^a^
*Acaricides*						
If resources, including funding and training opportunities, were available, would your agency be interested in developing the capacity to apply acaricides to suppress ticks?	Yes	71% (73/103)	75% (33/44)	76% (25/33)	58% (15/26)	N.S 0.488
	No	29% (30/103)	25% (11/44)	24% (8/33)	42% (11/26)	
If your agency were to apply acaricides, would it treat private property?	Yes	30% (22/73)	9% (3/33)	32% (8/25)	73% (11/15)	*P* = 0.003*
	No	70% (51/73)	91% (30/33)	68% (17/25)	27% (4/15)	
*Rodent-targeted interventions*						
If resources, including funding and training opportunities, were available, would your agency be interested in developing the capacity to deploy rodent-targeted tick suppression?	Yes	58% (64/110)	59% (30/51)	63% (20/32)	52% (14/27)	N.S *P* = 0.787
	No	42% (46/110)	41% (21/51)	38% (12/32)	48% (13/27)	
If your agency were to deploy rodent-targeted tick suppression, would it treat private property?	Yes	33% (21/63)	17% (5/29)	30% (6/20)	71% (10/14)	*P* = 0.012*
	No	67% (42/63)	83% (24/29)	70% (14/20)	29% (4/14)	
*Habitat and landscape management*						
If resources, including funding and training opportunities, were available, would your agency be interested in developing the capacity to deploy habitat or landscape management tick suppression?	Yes	50% (53/107)	50% (25/50)	53% (17/32)	44% (11/25)	N.S *P* = 0.788
	No	50% (54/107)	50% (25/50)	47% (15/32)	56% (14/25)	
If your agency were to deploy habitat or landscape management tick suppression, would it target private properties?	Yes	26% (14/53)	12% (3/25)	24% (4/17)	64% (7/11)	*P* = 0.024*
	No	74% (39/53)	88% (22/25)	77% (13/17)	36% (4/11)	
*Deer-targeted intervention*						
If resources, including funding and training opportunities, were available, would your agency be interested in developing the capacity to deploy deer-targeted tick suppression?	Yes	43% (46/107)	49% (24/49)	39% (12/31)	37% (10/27)	N.S *P* = 0.695
	No	57% (61/107)	51% (25/49)	61% (19/31)	63% (17/27)	
Would your agency deploy deer-targeted tick suppression on private property?	Yes	28% (13/46)	13% (3/24)	25% (3/12)	70% (7/10)	*P* < 0.001*
	No	72% (33/46)	88% (21/24)	75% (9/12)	30% (3/10)	

*Significant difference at an alpha-level of 0.05

*N.S.* Non-significant

^a^Chi-squared *P*-values correspond to tests comparing responses between the three regions, been corrected for multiple comparisons using the Benjamini–Hochberg method

### Obstacles to program development or expansion

Regarding the expansion of currently offered tick-related services or the development of new services, responding VCPs were asked to rank from one (greatest obstacle) to five (lowest obstacle) whether equipment, funding, personnel, standardized protocols, or training posed the greatest challenge. Funding was consistently ranked as the number one obstacle across all educational services, tick surveillance, and tick suppression activities. Personnel was consistently ranked as the second largest obstacle, with two exceptions. The first being that training was indicated to be a bigger obstacle than personnel for the development of tick suppression services using deer-targeted interventions. Training was also identified as a bigger obstacle than personnel for the development of new programs to educate the public about tick suppression methods (Tables 6 and 7).

**Table 6:** Ranking by responding vector control programs on a scale of 1 (greatest obstacle) to 5 (lowest obstacle) regarding whether equipment, funding, personnel, standardized protocols or training posed the greatest challenge to the expansion or development of surveillance and educational services

Tick-related services	Ranking score^a^					Number of responding VCPs
*Tick surveillance*						
Program expansion	2.04 Funding	2.57 Personnel	3.37 Standardized protocols	3.37 Training	3.65 Equipment	46
Program development	2.53 Funding	2.76 Personnel	3.06 Training	3.24 Equipment	3.31 Standardized protocols	50
*Tick control efficacy assessment*						
Program expansion^b^	N.A	N.A	N.A	N.A	N.A	0
Program development	2.17 Funding	2.63 Personnel	3.28 Equipment	3.41 Training	3.50 Standardized protocols	48
*Tick suppression guidance*						
Program expansion	2.29 Funding	2.47 Personnel	3.12 Training	3.21 Standardized protocols	3.91 Equipment	34
Program development	2.45 Funding	2.76 Training	3.10 Personnel	3.14 Standardized protocols	3.57 Equipment	42
*Educational materials on tick bite prevention*^c^						
Program expansion	2.14 Funding	2.76 Personnel	3.17 Access to digital educational material	3.19 Access to physical educational material	3.73 Training	42
Program development	2.44 Funding	2.79 Personnel	3.00 Access to physical educational material	3.30 Access to digital educational material	3.47 Training	43

*N.A.* Not applicable,* VCPs* vector control programs

^a^The values represent the mean ranking across the total number of responses

^b^No program offered this service, so obstacles to program expansion was not measured

^c^Options differed for this section as equipment and standardized protocols were not applicable to the development of this service

**Table 7:** Ranking by vector control programs on a scale of 1 (greatest obstacle) to 5 (lowest obstacle) regarding whether equipment, funding, personnel, standardized protocols or training posed the greatest challenge to the development of tick suppression services using one of four methods

Tick suppression services	Ranking score^a^					Number of responding VCPs
*Acaricides*						
Program development	2.21 Funding	2.95 Personnel	3.18 Standardized protocols	3.27 Equipment	3.39 Training	73
*Rodent-targeted interventions*						
Program development	2.33 Funding	2.8 Personnel	3.10 Training	3.32 Standardized protocols	3.44 Equipment	63
*Habitat and landscape management*						
Program development	2.32 Funding	3.02 Personnel	3.09 Training	3.28 Equipment	3.28 Standardized protocols	53
*Deer-targeted interventions*						
Program development	2.43 Funding	3.00 Training	3.04 Personnel	3.24 Standardized protocols	3.28 Equipment	46

* VCPs* Vector control programs

^a^The values represent the mean ranking across the total number of responses

### Interest and cost of tick management programmatic activities

Among the 107 VCPs that identified which services they would be likely to include as part of a comprehensive tick management program if funding and other resources were not a limitation, 90% (*N* = 96 responding VCPs) would likely include tick bite prevention education and 89% (*N* = 95 responding VCPs) would likely include tick surveillance to assess tick bite risk. Somewhat surprisingly, given that resources were not considered to be a limitation in this question, only 74% (*N* = 79 responding VCPs) would likely include offering tick suppression guidance and 68% (*N* = 73 responding VCPs) would likely include offering suppression of tick populations on public properties as an option. Substantially fewer VCPs listed tick suppression efficacy assessments for other pest control operators (29%, *N* = 31 responding VCPs) or suppressing tick populations on private properties (15%, *N* = 16 responding VCPs) as services they were likely to offer (Table 8).

**Table 8:** The interest of vector control programs to provide different services that might be included in a comprehensive tick management program

Service	VCPs interested in offering service^a^			
	All regions combined	Northeast region	Upper Midwest region	Pacific Coast region
Tick bite prevention education	90% (96/107)	92% (47/51)	87% (27/31)	88% (22/25)
Tick surveillance	89% (95/107)	82% (42/51)	99% (30/31)	92% (23/25)
Tick suppression guidance for property owners and managers	74% (79/107)	75% (38/51)	71% (22/31)	76% (19/25)
Suppression of tick populations on public properties	68% (73/107)	75% (38/51)	68% (21/31)	56% (14/25)
Tick suppression efficacy assessments for other operators	29% (31/107)	29% (15/51)	23% (7/31)	36% (9/25)
Suppression of tick populations on private properties	15% (16/107)	10% (5/51)	10% (3/31)	32% (8/25)

* VCPs* Vector control programs

^a^Values are shown as the percentage of responding VCPs, with* n*/*N* VCPs shown in parentheses

We also asked VCPs to estimate the cost for providing each service they selected. These values were expressed as a percentage that the responding VCP’s current budget would need to increase to cover the costs of each service. The majority of responding VCPs believed that tick bite prevention education (80%, 77/96 of responding VCPs), tick suppression guidance (77%, 61/79), tick surveillance (63%, 60/95) and efficacy assessments for private operators (58%, 18/31) would each require an increase in their current budget of 0% to 25%. The estimated cost of direct activities to suppress ticks were more varied, with 77% (56/73) of VCPs believing that their budget would need to increase by somewhere between 0% and 75% to offer tick suppression on public properties; 81% (13/16) estimated they would require a similar increase to offer tick suppression services on private properties (Table 9).

**Table 9:** The perceived relative cost to vector control programs for providing different services that might be included in a comprehensive tick management program

Service	Approximate % increase in program budget needed	All regions combined	Northeast region	Upper Midwest region	Pacific Coast region
Tick bite prevention education	0–25	80% (77/96)	83% (39/47)	74% (20/27)	82% (18/22)
	26–50	10% (10/96)	9% (4/47)	19% (5/27)	5% (1/22)
	51–75	5% (5/96)	4% (2/47)	4% (1/27)	9% (2/22)
	76–100	4% (4/96)	4% (2/47)	4% (1/27)	5% (1/22)
Tick surveillance	0–25	63% (60/95)	64% (27/42)	57% (17/30)	70% (16/23)
	26–50	15% (14/95)	19% (8/42)	10% (3/30)	13% (3/23)
	51–75	14% (13/95)	10% (4/42)	23% (7/30)	9% (2/23)
	76–100	8% (8/95)	7% (3/42)	10% (3/30)	9% (2/23)
Tick suppression guidance for property owners and managers	0–25	77% (61/79)	76% (29/38)	68% (15/22)	90% (17/19)
	26–50	14% (11/79)	16% (6/38)	18% (4/22)	5% (1/19)
	51–75	6% (5/79)	8% (3/38)	9% (2/22)	0% (0/19)
	76–100	3% (2/79)	0% (0/38)	5% (1/22)	5% (1/19)
Suppression of tick populations on public properties	0–25	29% (21/73)	13% (5/38)	48% (10/21)	43% (6/14)
	26–50	32% (23/73)	45% (17/38)	14% (3/21)	21% (3/14)
	51–75	16% (12/73)	21% (8/38)	10% (2/21)	14% (2/14)
	76–100	23% (17/73)	21% (8/38)	29% (6/21)	21% (3/14)
Suppression efficacy assessments for other operators	0–25	58% (18/31)	60% (9/15)	57% (4/7)	56% (5/9)
	26–50	32% (10/31)	33% (5/15)	43% (3/7)	22% (2/9)
	51–75	3% (1/31)	7% (1/15)	0% (0/7)	0% (0/9)
	76–100	6% (2/31)	0% (0/15)	0% (0/7)	22% (2/9)
Suppression of tick populations on private properties	0–25	25% (4/16)	20% (1/5)	33% (1/3)	25% (2/8)
	26–50	25% (4/16)	20% (1/5)	33% (1/3)	25% (2/8)
	51–75	31% (5/16)	40% (2/5)	33% (1/3)	25% (2/8)
	76–100	19% (3/16)	20% (1/5)	0% (0/3)	25% (2/8)

Costs are expressed as the percent increase needed to a program’s budget to provide each service

We also asked whether VCPs believed that the public in their jurisdiction would support a tax increase to enable them to offer new or expanded tick-related services. Overall, only 21% (22/107) of responding VCPs believed that the public would support a tax increase. In the Northeast, 29% (15/51) of responding VCPs believed a tax increase would be supported in their area. By contrast, only 12% (3/25) of respondents from the Pacific Coast and 10% (3/31) of respondents in the Upper Midwest believed the public would support a tax increase. The overall differences between regions were not large enough to be significant (*df* = 2, *χ*^2^ = 5.96, *P* = 0.170).

## Discussion

### Main findings

Of the 118 VCPs which participated in our survey, 70% provided tick bite prevention education and 48% conducted tick surveillance, but only 11% conducted any form of tick suppression (in most cases the application of acaricides to vegetation). Our findings are similar to those from two previous surveys, including one in the state of New Jersey which included a combined 43 county mosquito control programs and local health departments [16], and a national survey which included 140 vector-borne disease professionals [15]. These studies targeted entities with a presumed interest in tick-related services in areas that are endemic for tick-borne diseases (New Jersey [16]) or via a targeted recruitment strategy [15]. Another national survey [13, 14] focused broadly on local vector programs across the USA without consideration for tick-borne disease incidence. As this later survey included many areas with relatively low risk for *Ixodes*-borne diseases, it was not surprising that lower percentages of the responding programs (*N* = 483) reported providing public education regarding ticks and tick-borne diseases (35%) or conducting tick surveillance (21%) or tick control (3%). It also should be noted that nearly two thirds of the responding programs in this NACCHO survey were managed by local health departments or other local entities that may have broad responsibilities (e.g. public works) and only one third were completely dedicated to vector control. Finally, Dye-Braumuller et al. surveyed 150 publicly-funded VCPs in the southeastern USA, where tick-borne diseases associated with *Amblyomma* ticks predominate, and noted that while 96% of the VCPs reported addressing mosquitoes, only 8% addressed ticks [27]. Taken together, our survey and those conducted previously [13–16, 27] indicate limited engagement of VCPs in tick suppression, and modest engagement in tick surveillance activities, with engagement in tick bite prevention education being relatively common in *Ixodes*-borne disease endemic areas.

Our survey responses revealed an interest among VCPs in *Ixodes*-borne disease endemic areas to expand their tick-related services. Roughly 90% of entities favored including tick bite prevention education and tick surveillance and 68% favored inclusion of tick suppression on public lands. As with the previous study in New Jersey [16], fewer VCPs (15%) favored providing tick suppression on private properties. In our survey, the primary challenges to developing new tick management capacity, or to expanding existing capacity, were universally reported to be funding and personnel limitations. Again, this finding agrees with the results of previous national and statewide studies [15, 16]. Regarding tick control, these studies also indicated a concern about a lack of best management practices and limited evidence for the impact of large-scale tick management practices suitable for public lands [15, 16].

Overall, our survey revealed strong interest among the responding VCPs, which predominantly represented county level jurisdictions, to develop or strengthen their capacity to address ticks. However, respondents also note consistently that this cannot be achieved without the VCPs gaining additional resources. This main finding echoes a previous survey effort focused on New Jersey, where it was concluded that, given adequate funding, the majority of these entities were willing to create new or expand existing tick management programs [16]. Securing the resources needed to develop and maintain such programs will be critical to the development of a national public health strategy to prevent and control tick-borne diseases [28]. In our survey, only 21% of responding VCPs believed that the public in their jurisdiction would support a tax increase to offer new tick-related services. However, a recent survey of the public in a Lyme disease endemic area in the Upper Midwest indicated that 81% of citizens would be willing to pay at least $10 more per year in taxes for area-wide tick suppression services [6]. As noted by Mader et al., there is a need to explore different solutions to sustainably fund tick management programs as a single funding model may not be the best option in all situations [15].

### Tick bite prevention education and tick suppression guidance

As in the previous survey conducted in New Jersey [15], most responding VCPs in our survey provided educational information on tick-bite prevention to the public in the form of VCP-generated materials or links to other resources (e.g. websites with tick-bite prevention information provided by state health departments, CDC, or the U.S. Environmental Protection Agency). Tick-bite prevention education is affordable, and dissemination of this information does not require specialized skills not already possessed by most VCPs. There is no doubt that educational materials should be a cornerstone for a publicly-funded comprehensive tick management program. However, tick-bite prevention measures are not widely and consistently used by the public [5, 6]. In our survey, the ability to effectively communicate with the public was indicated as a concern for many VCPs (Supplemental file 2). More effective communication about the need for people to protect themselves against tick bites, leading to actual behavior change, is a critical area for improvement.

Educational guidance specific to tick suppression was offered by some of the VCPs that responded to our survey, but less frequently than tick bite prevention education. The most common tick suppression method for which responding VCPs provided guidance was habitat or landscape management. Provision of actionable guidance for other tick suppression methods may be more challenging as they require the use of acaricides and in some cases target vertebrate hosts. For example, the broadcast of acaricides may be controversial due to perceived environmental or health effects, and often their deployment requires specialized training and licensing. Some programs may also be reluctant to recommend specific tick control methods if clear evidence for their ability to reduce human tick bites or tick-borne disease is lacking [11, 18, 29, 30].

### Tick surveillance

Tick surveillance activities aimed at assessing tick bite risk were conducted by about half of the VCPs who responded to our survey, and over 80% of the VCPs not conducting tick surveillance were interested in developing that capacity. This held true across all three study regions. Strong interest in tick surveillance may be related, in part, to CDC providing funding for this activity via state health departments, as well as the availability of comprehensive guidance from CDC for the development of tick surveillance programs [3, 31–33]. Prior to the initiation of CDC’s national tick surveillance program, respondents to a previous survey cited funding and lack of guidance as barriers to conducting tick surveillance [15]. Most VCPs reported that they use tick surveillance data to identify high-risk areas, with few using tick surveillance data to direct or evaluate tick suppression deployed by their program. This is an unsurprising result given how few responding agencies conduct tick suppression activities. No agency indicated whether they evaluated the efficacy of tick suppression deployed by private landowners or pest control firms, which remains a knowledge gap.

We did not ask questions about specific reasons for conducting tick surveillance, but such information was generated in previous studies [15, 16]. Frequently reported reasons for conducting tick surveillance included detection and reporting of the local presence of vector ticks by species; detection of the presence and prevalence of tick-borne pathogens in ticks; and monitoring and mapping the presence and abundance of ticks by species, including the monitoring of patterns of geographic spread. It also should be noted that we did not distinguish between active and passive tick surveillance in our survey. This would be interesting to explore in the future as passive tick surveillance efforts are on the rise in the USA [21, 34].

### Tick suppression

In contrast to the vast majority of VCPs reportedly being engaged in mosquito control (98%), only a small percentage (11%) of the VCPs in our survey reported being engaged in tick suppression activities. These VCPs were located predominantly in the Northeast and used synthetic acaricides applied to the landscape as their primary tick control method. However, across all study regions there was substantial interest among VCPs in developing tick control capacity if adequate resources were to be made available. The overall percentage of VCPs with interest in developing the capacity to deploy specific tick control strategies ranged from 71% for the application of acaricides to vegetation to 58% for rodent-targeted methods, 50% for habitat and landscape management and 43% for deer-targeted methods. The strong interest in using acaricide application to control ticks is not surprising as previous surveys of both public and private pest control operators indicated that the application of synthetic acaricides was their most commonly used tick suppression method [12, 16, 36, 37]. This may be, in part, because many agencies already use pesticide application methods to target mosquitoes [13], and there is broad evidence that acaricides can suppress ticks in residential settings [38]. Overall, responding VCPs were less interested in using other tick suppression methods. Rodent-targeted and habitat management methods have shown some potential to reduce questing tick densities, but their efficacy has varied dramatically across evaluations [11, 35, 39]. The use of deer-targeted interventions can be particularly complex due to potential regulatory issues [5, 40]. Moreover, as previously noted, there is very limited evidence that tick suppression results in reductions in human tick bites or the number of tick-borne disease cases. The cost of upscaling tick suppression operations to the city, county or state-level must also be determined before these services can be widely offered by VCPs [17].

In our survey, interest in providing tick suppression services on private properties was limited. The expressed hesitancy of VCPs to target private land is particularly notable as tick bite risk is believed to be highest in peridomestic settings, at least in the Northeast [7–10]. Only roughly one third (26–33%) of those VCPs in our survey that were broadly interested in developing tick control capacity on public lands were willing to consider including private properties for tick suppression activities. This finding aligns with the previous study from New Jersey [16] where only 22% of county mosquito control programs indicated interest in suppressing ticks on private land, in contrast with 56% being interested in suppressing ticks on public land. In our survey, tick control on private properties was also ranked as a low priority among potential components of a comprehensive tick management program. Reluctance to control ticks specifically on private properties may be associated with logistical considerations (e.g. need for homeowner permission to access and treat the properties) as well as legal constraints regarding tick control on private land. Responding VCPs in the Pacific Coast states were significantly more likely to be willing to target private land than VCPs in other regions in our survey; this may be partially due to the amount of federal land in the western USA, often with complex regulatory systems [41, 42], which may increase the difficulty of conducting tick suppression operations on public lands in the west. Given the results of our survey, it appears that privately owned pest control firms, together with homeowners, will likely be the primary parties responsible for tick suppression on residential properties, particularly in the eastern USA, for the foreseeable future. However, VCPs can still assist in this effort by providing locally relevant guidelines to homeowners and private pest control firms regarding best practices for residential tick control and how to assess the efficacy of tick suppression efforts.

### Study limitations

The Northeast region represented a large proportion of our survey responses, likely because this region has a large number of states (Fig. 1) and numerous small counties. Moreover, our survey results in the Northeast were driven disproportionally by New Jersey and Pennsylvania, while those for the Pacific Coast were driven predominantly by VCPs in California. The views of VCPs in these states may not be representative of VCPs across all states in the targeted regions. It should also be noted that our survey focused on parts of the USA where *Ixodes* species are the primary vectors of tick-borne disease agents, thus likely introducing some bias in our conclusions relative to parts of the country where *Amblyomma* or *Dermacentor* species are dominant vectors. We were also not able to determine why some agencies consistently indicated that ticks were not a concern in their jurisdiction. They may have done so because acarological risk was low in their service area, or it may have been due to a lack of public concern.

Our results indicated that implementing new tick-related services were contingent on resource availability, but it is possible that responding agencies were bounded by the reality of their funding situations. The high cost of area-wide tick suppression [17] may have led many to select less costly surveillance and educational options to include as part of their hypothetical comprehensive tick management programs. It is also possible that the lack of demonstrated efficacy for tick suppression methods to reduce human tick bites or tick-borne disease [11, 18, 29, 30] caused agencies not to consider this service. It is notable that no survey has attempted to determine whether the governing bodies responsible for funding VCPs would prioritize increasing funding for the development of tick-related services. Considering the importance of funding indicated in our survey and others [16, 27], the priorities of these governing bodies are likely to play an important role in the development of comprehensive tick management programs.

Our survey did not measure the familiarity of responding VCPs with different tick suppression methods, but the previous study from New Jersey did so. The authors of that study reported that county mosquito control programs were familiar with some tick suppression methods, particularly the use of synthetic acaricides (44% of respondents indicated they were ‘very familiar’ with this method). Between 11% and 33% indicated being ‘very familiar’ with the other potential tick suppression methods that were listed [16]. Less than half of respondents in the New Jersey survey indicated a high degree of familiarity with even the most commonly used tick suppression method (acaricide application). Such lack of knowledge may have caused VCPs responding to our survey to be hesitant to indicate they would include tick suppression as a component of a comprehensive tick management program.

A large number of responding VCPs indicated an interest in expanding tick-related services within their existing programs. However, follow-up questions were not adequate to resolve what that expansion would include (e.g. conducting the same activities at larger scale, including additional approaches or methodologies). While we were able to quantify an interest in programmatic expansion, follow-up surveys are needed to better understand how much and in what ways VCPs might expand their services. It would also be useful to determine a per capita dollar amount that would be required for VCPs to provide these tick-related services. This would connect results from previous surveys of the public [6] with the cost of developing comprehensive tick management programs. These costs could also be compared with current operating budgets for VCPs offering mosquito control services to better determine how much their budgets would need to increase to begin offering tick suppression services.

## Conclusions

Despite our survey targeting geographic regions where ticks and tick-borne diseases are persistent and increasing public health concerns, only 11% of VCPs reported they took direct action to suppress ticks questing in the environment. Funding and personnel were the most cited reasons for not providing tick control services. If funding and resources were not a limitation, nearly two thirds (68%) of VCPs responded they would consider including tick suppression services on public land. However, even if resources were not a limitation, only 15% of VCPs responded they would offer tick suppression services on private properties. These responses demonstrate a substantial gap in current and aspirational tick control services provided by VCPs. Given the hesitancy of VCPs to target private land for tick suppression and the high risk for tick bites in peridomestic settings, at least in the Northeast, suppression of ticks on residential properties by private pest control operators will likely play an important role in the tick suppression landscape in the USA for the foreseeable future. Nevertheless, VCPs can still assist in this effort by providing locally relevant guidelines to homeowners and private pest control firms regarding best practices for residential tick suppression efforts and associated efficacy evaluations. Publicly-funded VCPs are also well positioned to educate the public on tick bite prevention measures and to collect tick surveillance data which provides information on risk of human encounters with ticks within their jurisdictions.

### Supplementary Information

Supplemental Material 1.

Supplemental Material 2.

Supplemental Material 3.

## Data Availability

The data that support the findings of this study are available from the corresponding author upon reasonable request.
