# Supplementary material for: Willingness and capacity of publicly-funded vector control programs in the USA to engage in tick management
Source: Parasit Vectors. 2024 Jul 22;17:316. doi: 10.1186/s13071-024-06400-8 (PMC11265149; doi:10.1186/s13071-024-06400-8)
Supplement: Supplementary file 1 — Supplemental Material 1. [file 13071_2024_6400_MOESM1_ESM.pdf]

Data Dictionary Codebook

	#	Variable / Field Name	Field Label<br>*Field Note*	Field Attributes (Field Type, Validation, Choices, Calculations, etc.)
Instrument: **Subsection 1: General Information** (subsection_1_general_information) Enabled as survey				
	1	[record_id]	Record ID	text
	2	[sub1_a_agency_name]	Section Header: *General agency and organizational information*<br>What is the name of your agency?	text, Required

	3	[sub1_h_state]	Please select the state in which your office is located:	dropdown, Required<br>1 CA<br>2 CT<br>3 DE<br>4 IA<br>5 IL<br>6 IN<br>7 MA<br>8 MD<br>9 ME<br>10 MI<br>11 MN<br>12 NH<br>13 NJ<br>14 NY<br>15 OH<br>16 OR<br>17 PA<br>18 RI<br>19 VA<br>20 VT<br>21 WA<br>22 WI<br>23 WV

	4	[sub1_b_agency_type]	What type of agency do you work for?	radio, Required<br>1 mosquito or vector control district not incorporated in another agency at your jurisdictional level<br>2 private firm with contract for community-wide vector control<br>3 public health agency<br>4 environmental health agency<br>5 public works<br>6 animal control<br>7 other
	5	[sub1_b_1_other_specify]<br>Show the field ONLY if:<br>[sub1_b_agency_type] = '7'	Please describe other agency type:	text
	6	[sub1_c_agency_jurisdiction]<br>Show the field ONLY if:<br>[sub1_b_agency_type] = '1' or<br>[sub1_b_agency_type] = '3' or<br>[sub1_b_agency_type] = '4' or<br>[sub1_b_agency_type] = '5' or<br>[sub1_b_agency_type] = '6' or<br>[sub1_b_agency_type] = '7'	What is the jurisdictional level of your agency?	radio, Required<br>1 city<br>2 county<br>3 state<br>4 regional or multi-state<br>5 other
	7	[sub1_d_program_size]<br>Show the field ONLY if:<br>[sub1_b_agency_type] = '1' or<br>[sub1_b_agency_type] = '3' or<br>[sub1_b_agency_type] = '4' or<br>[sub1_b_agency_type] = '5' or<br>[sub1_b_agency_type] = '6' or<br>[sub1_b_agency_type] = '7'	Please indicate the size of the population that your program serves:	radio, Required<br>1 0 - 9,999<br>2 10, 000 - 24,999<br>3 25, 000 - 49,999<br>4 50, 000 - 99,999<br>5 100, 000 - 249,999<br>6 250, 000 - 499,999<br>7 500, 000 - 999,999<br>8 1, 000,000 +

<table>
<tr><td></td><td>8</td><td>[sub1_h]</td><td>We encourage groups of people working within the same agency or organization to take this survey together, if possible. How many people from your organization are participating in answering this survey today?</td><td>radio, Required<table><tr><td>1</td><td>1</td></tr><tr><td>2</td><td>2</td></tr><tr><td>3</td><td>3</td></tr><tr><td>4</td><td>4</td></tr><tr><td>5</td><td>5</td></tr><tr><td>6</td><td>>5</td></tr></table></td></tr>
<tr><td></td><td>9</td><td>[sub1_e_job_rank]
Show the field ONLY if:
[sub1_b_agency_type] = '1' or
[sub1_b_agency_type] = '3' or
[sub1_b_agency_type] = '4' or
[sub1_b_agency_type] = '5' or
[sub1_b_agency_type] = '6' or
[sub1_b_agency_type] = '7'</td><td>What are the job levels, ranks, titles, or roles of the people currently answering this survey?[select all that apply]</td><td>checkbox, Required<table><tr><td>1</td><td>sub1_e_job_rank___1</td><td>director</td></tr><tr><td>2</td><td>sub1_e_job_rank___2</td><td>supervisor</td></tr><tr><td>3</td><td>sub1_e_job_rank___3</td><td>superintendent</td></tr><tr><td>4</td><td>sub1_e_job_rank___4</td><td>biologist</td></tr><tr><td>5</td><td>sub1_e_job_rank___5</td><td>entomologist</td></tr><tr><td>6</td><td>sub1_e_job_rank___6</td><td>epidemiologist</td></tr><tr><td>7</td><td>sub1_e_job_rank___7</td><td>technician</td></tr><tr><td>8</td><td>sub1_e_job_rank___8</td><td>other</td></tr></table></td></tr>
<tr><td></td><td>10</td><td>[sub1_e_1_other_specify]
Show the field ONLY if:
[sub1_e_job_rank(8)] = '1'</td><td>Please describe any other role(s):</td><td>text</td></tr>
<tr><td></td><td>11</td><td>[sub1_g_vector_control]
Show the field ONLY if:
[sub1_b_agency_type] = '1' or
[sub1_b_agency_type] = '3' or
[sub1_b_agency_type] = '4' or
[sub1_b_agency_type] = '5' or
[sub1_b_agency_type] = '6' or
[sub1_b_agency_type] = '7'</td><td>Does your agency take direct action (e.g. pesticide application / habitat modification / biological control) to control vector populations for public health purposes?</td><td>yesno, Required<table><tr><td>1</td><td>Yes</td></tr><tr><td>0</td><td>No</td></tr></table></td></tr>
</table>

<table>
<tr><td></td><td>12</td><td>[sub1_g_1_vector_targets]
Show the field ONLY if:
[sub1_g_vector_control] = '1'</td><td>What disease vectors or arthropod pests does your agency currently target?[select all that apply]</td><td>checkbox, Required
<table>
<tr><td>1</td><td>sub1_g_1_vector_targets___1</td><td>ticks</td></tr>
<tr><td>2</td><td>sub1_g_1_vector_targets___2</td><td>mosquitoes</td></tr>
<tr><td>3</td><td>sub1_g_1_vector_targets___3</td><td>fleas</td></tr>
<tr><td>4</td><td>sub1_g_1_vector_targets___4</td><td>bed bugs</td></tr>
<tr><td>5</td><td>sub1_g_1_vector_targets___5</td><td>other</td></tr>
</table></td></tr>
<tr><td></td><td>13</td><td>[sub1_g_1_a_other]
Show the field ONLY if:
[sub1_g_1_vector_targets(5)] = '1'</td><td>Please describe any other disease vector(s) your agency targets:</td><td>text</td></tr>
<tr><td></td><td>14</td><td>[sub1_g_1_a_tick_targets]
Show the field ONLY if:
[sub1_g_1_vector_targets(1)] = '1'</td><td>What tick species does your agency target for control operations?
[select all that apply]</td><td>checkbox, Required
<table>
<tr><td>1</td><td>sub1_g_1_a_tick_targets___1</td><td>Ixodes scapularis</td></tr>
<tr><td>2</td><td>sub1_g_1_a_tick_targets___2</td><td>Ixodes pacificus</td></tr>
<tr><td>3</td><td>sub1_g_1_a_tick_targets___3</td><td>Dermacentor andersoni</td></tr>
<tr><td>4</td><td>sub1_g_1_a_tick_targets___4</td><td>Dermacentor occidentalis</td></tr>
<tr><td>5</td><td>sub1_g_1_a_tick_targets___5</td><td>Dermacentor variabilis</td></tr>
<tr><td>6</td><td>sub1_g_1_a_tick_targets___6</td><td>Amblyomma americanum</td></tr>
<tr><td>7</td><td>sub1_g_1_a_tick_targets___7</td><td>Amblyomma maculatum</td></tr>
<tr><td>8</td><td>sub1_g_1_a_tick_targets___8</td><td>Haemaphysalis longicornis</td></tr>
<tr><td>9</td><td>sub1_g_1_a_tick_targets___9</td><td>Rhipicephalus sanguineus</td></tr>
<tr><td>10</td><td>sub1_g_1_a_tick_targets___10</td><td>other</td></tr>
<tr><td>11</td><td>sub1_g_1_a_tick_targets___11</td><td>do not know</td></tr>
</table></td></tr>
</table>

	15	[sub1_g_1_a_1_other_ticks]<br><br>Show the field ONLY if: [sub1_g_1_a_tick_targets(10)] = '1'	Please specify any other tick species your agency targets for control operations:	text
	16	[sub2_i]	How important do you think ticks and tick-borne diseases are in your jurisdiction?	radio, Required<br>1 not important<br>2 somewhat important<br>3 important<br>4 very important<br>5 extremely important
	17	[sub1_f_tick_control_pressure]<br><br>Show the field ONLY if: [sub1_b_agency_type] = '1' or [sub1_b_agency_type] = '3' or [sub1_b_agency_type] = '4' or [sub1_b_agency_type] = '5' or [sub1_b_agency_type] = '6' or [sub1_b_agency_type] = '7'	Has your agency faced any public or political pressure to start offering tick management services?	yesno, Required<br>1 Yes<br>0 No

	18	[sub1_f_1_pressure_sources]<br><br>Show the field ONLY if:<br>[sub1_f_tick_control_pressure] = '1'	What are the primary sources of pressure your agency faces? [select all that apply]	checkbox, Required<br>1 — sub1_f_1_pressure_sources___1 — public<br>2 — sub1_f_1_pressure_sources___2 — politicians (federal)<br>3 — sub1_f_1_pressure_sources___3 — politicians (state)<br>4 — sub1_f_1_pressure_sources___4 — politicians (local)<br>5 — sub1_f_1_pressure_sources___5 — federal agencies<br>6 — sub1_f_1_pressure_sources___6 — state-level agencies outside of your own<br>7 — sub1_f_1_pressure_sources___7 — county-level agencies outside of your own<br>8 — sub1_f_1_pressure_sources___8 — city-level agencies outside of your own<br>9 — sub1_f_1_pressure_sources___9 — pressure within your agency<br>10 — sub1_f_1_pressure_sources___10 — other
	19	[sub1_f_1]<br><br>Show the field ONLY if:<br>[sub1_f_1_pressure_sources(10)] = '1'	Please describe any other source(s) of pressure your agency faces:	text
	20	[sub1_g_2_contracting]<br><br>Show the field ONLY if:<br>[sub1_g_vector_control] = '0'	If your agency is not directly involved in vector control activities, is your agency responsible for contracting vector control to another agency or private company?	yesno, Required<br>1 — Yes<br>0 — No

	21	[sub1_g_2_1_contracting_agency]<br>Show the field ONLY if:<br>[sub1_g_2_contracting] = '1'	What type(s) of organization(s) do you contract for vector control in your jurisdiction? [select all that apply]	checkbox, Required<br>1 sub1_g_2_1_contracting_agency___1 standalone mosquito control district<br>2 sub1_g_2_1_contracting_agency___2 private vector control firm<br>3 sub1_g_2_1_contracting_agency___3 public health agency<br>4 sub1_g_2_1_contracting_agency___4 environmental health agency<br>5 sub1_g_2_1_contracting_agency___5 public works agency<br>6 sub1_g_2_1_contracting_agency___6 animal control<br>7 sub1_g_2_1_contracting_agency___7 other
	22	[sub1_g_2_1_a_other_contract]<br>Show the field ONLY if:<br>[sub1_g_2_1_contracting_agency(7)] = '1'	Please describe any other agency or organization you contract for vector control in your jurisdiction:	text
	23	[subsection_1_general_information_complete]	Section Header: *Form Status*<br>Complete?	dropdown<br>0 Incomplete<br>1 Unverified<br>2 Complete
Instrument: **Subsection 2: Tick surveillance and risk mitigation** (subsection_2_tick_surveillance_and_risk_mitigation) Enabled as survey				
	24	[sub2_a_identify_risk]	Section Header: *Capacity for tick surveillance, identification of high-risk areas for tick exposure, and public education and outreach regarding personal tick-bite prevention methods in high-risk areas*<br>Does your agency directly conduct tick surveillance (e.g. tick dragging / wildlife collections / veterinary submission / public submissions) to measure the risk of tick exposure in your jurisdiction?	yesno, Required<br>1 Yes<br>0 No

	25	[sub2_b]	Is there another agency or group that collects tick surveillance data in your jurisdiction?	yesno, Required<br>1 Yes<br>0 No
	26	[sub2_b_1]<br><br>Show the field ONLY if:<br>[sub2_b] = '1'	What other groups or agencies collect tick surveillance data within your jurisdiction?[select all that apply]	checkbox, Required<br>1 sub2_b_1___1 state or local department of health<br>2 sub2_b_1___2 state department of the environment / natural resources / agriculture<br>3 sub2_b_1___3 local parks and recreation agencies<br>4 sub2_b_1___4 academic institution / university extension services<br>5 sub2_b_1___5 local non-profit<br>6 sub2_b_1___6 other
	27	[sub2_b_1_a]<br><br>Show the field ONLY if:<br>[sub2_b_1(6)] = '1'	What other groups or agencies collect tick surveillance data in your jurisdiction?	text
	28	[sub2_a_1_funding]<br><br>Show the field ONLY if:<br>[sub2_a_identify_risk] = '1'	What are the funding sources to conduct tick surveillance activities?[select all that apply]	checkbox<br>1 sub2_a_1_funding___1 local property taxes<br>2 sub2_a_1_funding___2 state taxes<br>3 sub2_a_1_funding___3 other type of state funding<br>4 sub2_a_1_funding___4 county taxes<br>5 sub2_a_1_funding___5 town or city taxes<br>6 sub2_a_1_funding___6 private donations<br>7 sub2_a_1_funding___7 surcharge on services or goods<br>8 sub2_a_1_funding___8 federal funds<br>9 sub2_a_1_funding___9 other

<table>
<tr><td></td><td>29</td><td>[sub2_a_1_a_funding_other]

Show the field ONLY if:
[sub2_a_1_funding(9)] = '1'</td><td>Please describe any other funding source(s) for tick surveillance activities:</td><td>text</td></tr>
<tr><td></td><td>30</td><td>[sub2_a_2_surviellance_methods]

Show the field ONLY if:
[sub2_a_identify_risk] = '1'</td><td>What tick surveillance methods does your agency, or group of agencies, use to to measure the risk of tick exposure in your jurisdiction?[select all that apply]</td><td>checkbox, Required
<table>
<tr><td>1</td><td>sub2_a_2_surviellance_methods___1</td><td>collecting ticks from vegetation (e.g. tick dragging / flagging)</td></tr>
<tr><td>2</td><td>sub2_a_2_surviellance_methods___2</td><td>wildlife / small mammal surveys</td></tr>
<tr><td>3</td><td>sub2_a_2_surviellance_methods___3</td><td>tick submissions from public</td></tr>
<tr><td>4</td><td>sub2_a_2_surviellance_methods___4</td><td>veterinary data</td></tr>
<tr><td>5</td><td>sub2_a_2_surviellance_methods___5</td><td>deer check stations</td></tr>
<tr><td>6</td><td>sub2_a_2_surviellance_methods___6</td><td>carbon dioxide traps</td></tr>
<tr><td>7</td><td>sub2_a_2_surviellance_methods___7</td><td>other</td></tr>
</table></td></tr>
<tr><td></td><td>31</td><td>[sub2_a_2_a_surviellance_other]

Show the field ONLY if:
[sub2_a_2_surviellance_methods(7)] = '1'</td><td>Please describe any other surveillance methods your agency uses to measure the risk of tick exposure in your jurisdiction:</td><td>text</td></tr>
</table>

<table>
<tr><td></td><td>32</td><td>[sub2_a_3_using_data]

Show the field ONLY if:
[sub2_a_identify_risk] = '1'</td><td>In what ways does your agency use tick surveillance data? [select all that apply]</td><td>checkbox, Required<table>
<tr><td>1</td><td>sub2_a_3_using_data___1</td><td>mapping the local tick (or tickborne disease) exposure risk</td></tr>
<tr><td>2</td><td>sub2_a_3_using_data___2</td><td>informing the public of high-risk areas for exposure to ticks and tickborne disease agents through print or digital media</td></tr>
<tr><td>3</td><td>sub2_a_3_using_data___3</td><td>posting signage in high-use public areas</td></tr>
<tr><td>4</td><td>sub2_a_3_using_data___4</td><td>directing tick control efforts</td></tr>
<tr><td>5</td><td>sub2_a_3_using_data___5</td><td>evaluating tick control efforts</td></tr>
<tr><td>6</td><td>sub2_a_3_using_data___6</td><td>research</td></tr>
<tr><td>7</td><td>sub2_a_3_using_data___7</td><td>other</td></tr>
</table></td></tr>
<tr><td></td><td>33</td><td>[sub2_a_3_a_uses_other]

Show the field ONLY if:
[sub2_a_3_using_data(7)] = '1'</td><td>Please describe any other way(s) your agency uses surveillance data:</td><td>text</td></tr>
<tr><td></td><td>34</td><td>[sub2_a_4_pathogen_testing]

Show the field ONLY if:
[sub2_a_identify_risk] = '1'</td><td>Does your agency test the ticks it collects for human pathogens?</td><td>yesno, Required<table>
<tr><td>1</td><td>Yes</td></tr>
<tr><td>0</td><td>No</td></tr>
</table></td></tr>
<tr><td></td><td>35</td><td>[sub2_a_6_interest_starting]

Show the field ONLY if:
[sub2_a_identify_risk] = '0'</td><td>If resources, including funding and training opportunities, were available, would your agency be interested in developing the capacity to inform the public of high-risk areas for exposure to ticks and tickborne disease agents?</td><td>yesno, Required<table>
<tr><td>1</td><td>Yes</td></tr>
<tr><td>0</td><td>No</td></tr>
</table></td></tr>
</table>

<table>
<tr>
<td></td>
<td>36</td>
<td>[sub2_a_4_a_testing_agency]
Show the field ONLY if:
[sub2_a_4_pathogen_testing] = '1'</td>
<td>Who is responsible for testing the ticks your agency collects? [select all that apply]</td>
<td>checkbox, Required
<table>
<tr><td>1</td><td>sub2_a_4_a_testing_agency___1</td><td>your organization</td></tr>
<tr><td>2</td><td>sub2_a_4_a_testing_agency___2</td><td>county-level health department</td></tr>
<tr><td>3</td><td>sub2_a_4_a_testing_agency___3</td><td>county-level vector control</td></tr>
<tr><td>4</td><td>sub2_a_4_a_testing_agency___4</td><td>state-level health department</td></tr>
<tr><td>5</td><td>sub2_a_4_a_testing_agency___5</td><td>state-level vector control</td></tr>
<tr><td>6</td><td>sub2_a_4_a_testing_agency___6</td><td>state-level department of agriculture / environment</td></tr>
<tr><td>7</td><td>sub2_a_4_a_testing_agency___7</td><td>academic institution / university extension services</td></tr>
<tr><td>8</td><td>sub2_a_4_a_testing_agency___8</td><td>private laboratory</td></tr>
<tr><td>9</td><td>sub2_a_4_a_testing_agency___9</td><td>Centers for Disease Control and Prevention</td></tr>
<tr><td>10</td><td>sub2_a_4_a_testing_agency___10</td><td>other</td></tr>
</table>
</td>
</tr>
<tr>
<td></td>
<td>37</td>
<td>[sub2_a_4_a_1_other_agency]
Show the field ONLY if:
[sub2_a_4_a_testing_agency(10)] = '1'</td>
<td>Please specify any other agencies responsible for testing ticks your agency collects:</td>
<td>text</td>
</tr>
</table>

<table>
<tr><td></td><td>38</td><td>[sub2_a_4_b]

Show the field ONLY if:
[sub2_a_4_pathogen_testing] = '1'</td><td>What are the pathogens for which your agency tests? [select all that apply]</td><td>checkbox, Required
<table>
<tr><td>1</td><td>sub2_a_4_b___1</td><td>Anaplasma phagocytophilum</td></tr>
<tr><td>2</td><td>sub2_a_4_b___2</td><td>Babesia microti</td></tr>
<tr><td>3</td><td>sub2_a_4_b___3</td><td>Bourbon virus</td></tr>
<tr><td>4</td><td>sub2_a_4_b___4</td><td>Colorado tick fever virus</td></tr>
<tr><td>5</td><td>sub2_a_4_b___5</td><td>Borrelia burgdorferi</td></tr>
<tr><td>6</td><td>sub2_a_4_b___6</td><td>Borrelia miyamotoi</td></tr>
<tr><td>7</td><td>sub2_a_4_b___7</td><td>Borrelia mayonii</td></tr>
<tr><td>8</td><td>sub2_a_4_b___8</td><td>Ehrlichia chaffeensis</td></tr>
<tr><td>9</td><td>sub2_a_4_b___9</td><td>Ehrlichia ewingii</td></tr>
<tr><td>10</td><td>sub2_a_4_b___10</td><td>Ehrlichia muris eauclairensis</td></tr>
<tr><td>11</td><td>sub2_a_4_b___11</td><td>Francisella tularensis</td></tr>
<tr><td>12</td><td>sub2_a_4_b___12</td><td>Heartland virus</td></tr>
<tr><td>13</td><td>sub2_a_4_b___13</td><td>Powassan virus</td></tr>
<tr><td>14</td><td>sub2_a_4_b___14</td><td>Rickettsia parkeri</td></tr>
<tr><td>15</td><td>sub2_a_4_b___15</td><td>Rickettsia rickettsii</td></tr>
<tr><td>16</td><td>sub2_a_4_b___16</td><td>Other</td></tr>
</table></td></tr>
<tr><td></td><td>39</td><td>[sub2_a_4_other]

Show the field ONLY if:
[sub2_a_4_b(16)] = '1'</td><td>Please list any other pathogens for which your agency tests.</td><td>text</td></tr>
<tr><td></td><td>40</td><td>[sub2_a_5_expand_surviellance]

Show the field ONLY if:
[sub2_a_identify_risk] = '1'</td><td>Do you want to expand your agency's capacity to use or conduct tick surveillance to measure the risk of tick exposure in your jurisdiction?</td><td>yesno, Required
<table>
<tr><td>1</td><td>Yes</td></tr>
<tr><td>0</td><td>No</td></tr>
</table></td></tr>
<tr><td></td><td>41</td><td>[sub2_a_5_a_header]

Show the field ONLY if:
[sub2_a_5_expand_surviellance] = '1'</td><td>What would be most helpful to expand your agency's capacity to measure the risk of tick exposure in your jurisdiction?

Please rank below choices in order of priority, with highest priority as '1' and lowest priority as '5':</td><td>descriptive</td></tr>
</table>

	42	[sub2_a_5_a1]<br><br>Show the field ONLY if:<br>[sub2_a_5_expand_surviellance] = '1'	funding	radio (Matrix - ranking), Required<br>1 1<br>2 2<br>3 3<br>4 4<br>5 5
	43	[sub2_a_5_a2]<br><br>Show the field ONLY if:<br>[sub2_a_5_expand_surviellance] = '1'	personnel	radio (Matrix - ranking), Required<br>1 1<br>2 2<br>3 3<br>4 4<br>5 5
	44	[sub2_a_5_a3]<br><br>Show the field ONLY if:<br>[sub2_a_5_expand_surviellance] = '1'	equipment	radio (Matrix - ranking), Required<br>1 1<br>2 2<br>3 3<br>4 4<br>5 5
	45	[sub2_a_5_a4]<br><br>Show the field ONLY if:<br>[sub2_a_5_expand_surviellance] = '1'	standardized protocols	radio (Matrix - ranking), Required<br>1 1<br>2 2<br>3 3<br>4 4<br>5 5

	46	[sub2_a_5_a5]<br><br>Show the field ONLY if:<br>[sub2_a_5_expand_surviellance] = '1'	training	radio (Matrix - ranking), Required<br>1 | 1<br>2 | 2<br>3 | 3<br>4 | 4<br>5 | 5
	47	[sub2_a_5_a_1]<br><br>Show the field ONLY if:<br>[sub2_a_5_expand_surviellance] = '1'	Please describe anything else you might require to help expand your agency's capacity to conduct surveillance and identify high-risk areas:	text
	48	[sub2_a_5_b]<br><br>Show the field ONLY if:<br>[sub2_a_5_expand_surviellance] = '1'	Please select any other potential roadblocks to the development or expansion of your agency's capacity to measure the risk of tick exposure in your jurisdiction: [select all that apply]	checkbox, Required<br>1 | sub2_a_5_b___1 | constrained by legislative mandate<br>2 | sub2_a_5_b___2 | concerns for safety of staff<br>3 | sub2_a_5_b___3 | lack of public support<br>4 | sub2_a_5_b___4 | lack of administrative support<br>5 | sub2_a_5_b___5 | public not likely to use information<br>6 | sub2_a_5_b___6 | inability to effectively disseminate information<br>7 | sub2_a_5_b___7 | other
	49	[sub2_a_5_b_1]<br><br>Show the field ONLY if:<br>[sub2_a_5_b(7)] = '1'	Please describe any other reasons your agency might be uninterested in measuring the risk of tick exposure in your jurisdiction:	text
	50	[sub2_a_6_a_header]<br><br>Show the field ONLY if:<br>[sub2_a_6_interest_starting] = '1'	What would be most helpful to expand your agency's capacity to use or conduct tick surveillance to measure the risk of tick exposure in your jurisdiction?<br><br>Please rank below choices in order of priority, with highest priority as '1' and lowest priority as '5':	descriptive

	51	[sub2_a_6_a1]<br><br>Show the field ONLY if:<br>[sub2_a_6_interest_starting] = '1'	funding	radio (Matrix - ranking), Required<br>1 1<br>2 2<br>3 3<br>4 4<br>5 5
	52	[sub2_a_6_a2]<br><br>Show the field ONLY if:<br>[sub2_a_6_interest_starting] = '1'	personnel	radio (Matrix - ranking), Required<br>1 1<br>2 2<br>3 3<br>4 4<br>5 5
	53	[sub2_a_6_a3]<br><br>Show the field ONLY if:<br>[sub2_a_6_interest_starting] = '1'	equipment	radio (Matrix - ranking), Required<br>1 1<br>2 2<br>3 3<br>4 4<br>5 5
	54	[sub2_a_6_a4]<br><br>Show the field ONLY if:<br>[sub2_a_6_interest_starting] = '1'	standardized protocols	radio (Matrix - ranking), Required<br>1 1<br>2 2<br>3 3<br>4 4<br>5 5

	55	[sub2_a_6_a5]<br><br>Show the field ONLY if:<br>[sub2_a_6_interest_starting] = '1'	training	radio (Matrix - ranking), Required<br>1 1<br>2 2<br>3 3<br>4 4<br>5 5
	56	[sub2_a_6_a_1_capacity_other]<br><br>Show the field ONLY if:<br>[sub2_a_6_interest_starting] = '1'	Please describe anything else you might require to help expand your agency's capacity to conduct surveillance and identify high-risk areas:	text
	57	[sub2_a_6_e]<br><br>Show the field ONLY if:<br>[sub2_a_6_interest_starting] = '1'	Please select any other potential roadblocks to the development or expansion of your agency's capacity to measure the risk of tick exposure in your jurisdiction: [select all that apply]	checkbox, Required<br>1 sub2_a_6_e___1 constrained by legislative mandate<br>2 sub2_a_6_e___2 concerns for safety of staff<br>3 sub2_a_6_e___3 lack of public support<br>4 sub2_a_6_e___4 lack of administrative support<br>5 sub2_a_6_e___5 public not likely to use information<br>6 sub2_a_6_e___6 inability to effectively disseminate information<br>7 sub2_a_6_e___7 other
	58	[sub2_a_6_e_1]<br><br>Show the field ONLY if:<br>[sub2_a_6_e(7)] = '1'	Please describe any other reasons your agency might be uninterested in measuring the risk of tick exposure in your jurisdiction:	text

	59	[sub2_a_6_b_surveillance_resources]<br><br>Show the field ONLY if: [sub2_a_6_interest_starting] = '1'	If resources were available, what tick surveillance methods might your agency use?[select all that apply]	checkbox, Required<br>1 sub2_a_6_b_surveillance_resources___1 collecting ticks from vegetation (e.g. tick dragging / flagging)<br>2 sub2_a_6_b_surveillance_resources___2 tick submissions from public<br>3 sub2_a_6_b_surveillance_resources___3 veterinary data<br>4 sub2_a_6_b_surveillance_resources___4 other<br>5 sub2_a_6_b_surveillance_resources___5 do not know
	60	[sub2_a_6_b_1_surveillance_other]<br><br>Show the field ONLY if: [sub2_a_6_b_surveillance_resources(4)] = '1'	Please describe any other tick surveillance method(s) your agency might use:	text
	61	[sub2_a_6_c_surveillance_use]<br><br>Show the field ONLY if: [sub2_a_6_interest_starting] = '1'	How would your agency likely use tick surveillance data? [select all that apply]	checkbox, Required<br>1 sub2_a_6_c_surveillance_use___1 inform the public of high-risk areas<br>2 sub2_a_6_c_surveillance_use___2 inform deployment of tick control operations<br>3 sub2_a_6_c_surveillance_use___3 evaluate deployed tick control<br>4 sub2_a_6_c_surveillance_use___4 other

	62	[sub2_a_6_c_1_other_uses]<br>Show the field ONLY if:<br>[sub2_a_6_c_surveillance_use(4)] = '1'	Please describe any other ways your agency might use surveillance data:	text
	63	[sub2_a_6_d_why_not_interested]<br>Show the field ONLY if:<br>[sub2_a_6_interest_starting] = '0'	Why is your agency uninterested in measuring the risk of tick exposure in your jurisdiction? [select all that apply]	checkbox, Required<br>1 | sub2_a_6_d_why_not_interested___1 | ticks and tickborne disease are not a priority concern in our jurisdiction<br>2 | sub2_a_6_d_why_not_interested___2 | constrained by legislative mandate<br>3 | sub2_a_6_d_why_not_interested___3 | concerns for safety of staf[illegible]<br>4 | sub2_a_6_d_why_not_interested___4 | lack of public support<br>5 | sub2_a_6_d_why_not_interested___5 | lack of administrativ[illegible] support<br>6 | sub2_a_6_d_why_not_interested___6 | lack of traine[illegible] personnel<br>7 | sub2_a_6_d_why_not_interested___7 | public not likely to use information<br>8 | sub2_a_6_d_why_not_interested___8 | inability to effectively disseminate information

				9 | sub2_a_6_d_why_not_interested___9 | information already available elsewhere or collected by another organization<br>10 | sub2_a_6_d_why_not_interested___10 | other
	64	[sub2_a_6_d_1_not_interested_other]<br>Show the field ONLY if: [sub2_a_6_d_why_not_interested(10)] = '1'	Please describe any other reasons your agency might be uninterested in measuring the risk of tick exposure in your jurisdiction:	text
	65	[subsection_2_tick_surveillance_and_risk_mitigation_complete]	Section Header: *Form Status*<br>Complete?	dropdown<br>0 | Incomplete<br>1 | Unverified<br>2 | Complete

Instrument: **Subsection 3: Tick control methods** (subsection_3_tick_control_methods) Enabled as survey

	66	[sub3_header]	Capacity to deploy tick control targeting high-use public property or private property	descriptive

<table>
<tr><td></td><td>67</td><td>[sub3_a_finding_methods]</td><td>Where does your agency acquire information regarding available tick control methods?[select all that apply]</td><td>checkbox, Required
<table>
<tr><td>1</td><td>sub3_a_finding_methods___1</td><td>scientific literature</td></tr>
<tr><td>2</td><td>sub3_a_finding_methods___2</td><td>county health department</td></tr>
<tr><td>3</td><td>sub3_a_finding_methods___3</td><td>state health department</td></tr>
<tr><td>4</td><td>sub3_a_finding_methods___4</td><td>Centers for Disease Control and Prevention</td></tr>
<tr><td>5</td><td>sub3_a_finding_methods___5</td><td>university extension services</td></tr>
<tr><td>6</td><td>sub3_a_finding_methods___6</td><td>academic institution</td></tr>
<tr><td>7</td><td>sub3_a_finding_methods___7</td><td>meetings</td></tr>
<tr><td>8</td><td>sub3_a_finding_methods___8</td><td>companies who produce pest management products</td></tr>
<tr><td>9</td><td>sub3_a_finding_methods___9</td><td>local private pest management operators</td></tr>
<tr><td>10</td><td>sub3_a_finding_methods___10</td><td>professional groups (e.g. ESA / AMCA / ASTHO / NACCHO)</td></tr>
<tr><td>11</td><td>sub3_a_finding_methods___11</td><td>other</td></tr>
</table></td></tr>
<tr><td></td><td>68</td><td>[sub3_a_1]

Show the field ONLY if:
[sub3_a_finding_methods(11)] = '1'</td><td>Please describe any other source(s) your agency uses to acquire information regarding tick control methods:</td><td>text</td></tr>
</table>

	69	[sub3_b_control_frequency]	On average, how often does your agency deploy tick control?	radio, Required<br>1 multiple times per year<br>2 once a year<br>3 never<br>4 other
	70	[sub3_b_2_other_frequency]<br>Show the field ONLY if: [sub3_b_control_frequency] = '4'	Please describe any other frequency at which your agency deploys tick control:	text
	71	[sub3_b_1_annual_frequency]<br>Show the field ONLY if: [sub3_b_control_frequency] = '1'	Please provide an approximate number of tick control applications your agency deploys per year:	text
	72	[sub3_c_methods_used]<br>Show the field ONLY if: [sub3_b_control_frequency] = '1' or [sub3_b_control_frequency] = '2' or [sub3_b_control_frequency] = '4'	What, if any, tick control methods does your agency currently deploy?[select all that apply]	checkbox, Required<br>1 sub3_c_methods_used___1 application of acaricides (e.g. pesticide targeting ticks) to vegetation (synthetic, biological, or natural)<br>2 sub3_c_methods_used___2 deer-targeted intervention<br>3 sub3_c_methods_used___3 rodent-targeted intervention<br>4 sub3_c_methods_used___4 habitat /or landscape management<br>5 sub3_c_methods_used___5 other
	73	[sub3_c_1_other_method]<br>Show the field ONLY if: [sub3_c_methods_used(5)] = '1'	Please describe any other tick control method(s) your agency deploys:	notes

	74	[subsection_3_tick_control_methods_complete]	Section Header: *Form Status*<br>Complete?	dropdown<br>0 Incomplete<br>1 Unverified<br>2 Complete
Instrument: **Subsection 3a: Acaricide application** (subsection_3a_acaricide_application) Enabled as survey				
	75	[sub3a_i]<br>Show the field ONLY if:<br>[sub3_c_methods_used(1)] = '1'	Section Header: *Capacity to apply acaricides*<br>What types of acaricide products does your agency deploy for tick control?[select all that apply]	checkbox, Required<br>1 sub3a_i___1 synthetic chemical<br>2 sub3a_i___2 natural botanical product<br>3 sub3a_i___3 entomopathogenic fungus<br>4 sub3a_i___4 other
	76	[sub3a_i_1]<br>Show the field ONLY if:<br>[sub3a_i(4)] = '1'	Please describe any other type(s) of acaricide products your agency deploys for tick control?:	text
	77	[sub3a_a]<br>Show the field ONLY if:<br>[sub3_c_methods_used(1)] = '1'	What are the funding sources for your agency's acaricide applications?[select all that apply]	checkbox, Required<br>1 sub3a_a___1 local property taxes<br>2 sub3a_a___2 state taxes<br>3 sub3a_a___3 county taxes<br>4 sub3a_a___4 town or city taxes<br>5 sub3a_a___5 private donations<br>6 sub3a_a___6 surcharge on services or goods<br>7 sub3a_a___7 federal funds<br>8 sub3a_a___8 other
	78	[sub3a_a_1]<br>Show the field ONLY if:<br>[sub3a_a(8)] = '1'	Please describe any other funding source(s) your agency uses for acaricide applications:	text

	79	[sub3a_d]<br><br>Show the field ONLY if:<br>[sub3_c_methods_used(1)] = '1'	Do acaricide applications target private property, public property, or both?	radio, Required<br>1 | private properties<br>2 | public properties<br>3 | both private public properties
	80	[sub3a_b]<br><br>Show the field ONLY if:<br>[sub3a_d] = '2' or [sub3a_d] = '3'	What triggers your agency to apply acaricides on public property?[select all that apply]	checkbox, Required<br>1 | sub3a_b___1 | response to tick surveillance data<br>2 | sub3a_b___2 | response to human case data<br>3 | sub3a_b___3 | ongoing program deployed at predetermined sites<br>4 | sub3a_b___4 | based on public complaint or request<br>5 | sub3a_b___5 | other
	81	[sub3a_b_1]<br><br>Show the field ONLY if:<br>[sub3a_b(5)] = '1'	Please describe any other reason(s) that trigger your agency to apply acaricides on public property:	text
	82	[sub3a_d_2]<br><br>Show the field ONLY if:<br>[sub3a_d] = '3' or [sub3a_d] = '1'	What triggers your agency to apply acaricides on private property?[select all that apply]	checkbox, Required<br>1 | sub3a_d_2___1 | response to tick surveillance data<br>2 | sub3a_d_2___2 | response to human case data<br>3 | sub3a_d_2___3 | ongoing program deployed at predetermined sites<br>4 | sub3a_d_2___4 | landowner request<br>5 | sub3a_d_2___5 | other
	83	[sub3a_d_2_1]<br><br>Show the field ONLY if:<br>[sub3a_d_2(5)] = '1'	Please describe any other reason(s) that trigger your agency to deploy acaricides on private property:	notes

	84	[sub3a_c]<br>Show the field ONLY if:<br>[sub3a_d] = '2' or [sub3a_d] = '3'	Approximately how many public properties does your agency generally treat with acaricide applications per year?	radio, Required<br>1 1-5<br>2 5-10<br>3 10-50<br>4 >50
	85	[sub3a_j]<br>Show the field ONLY if:<br>[sub3a_d] = '3' or [sub3a_d] = '1'	Approximately how many private properties does your agency generally treat with acaricide applications per year?	radio, Required<br>1 1-5<br>2 5-10<br>3 10-50<br>4 >50
	86	[sub3a_d_1]<br>Show the field ONLY if:<br>[sub3a_d] = '2' or [sub3a_d] = '3'	What type(s) of public property does your agency target for acaricide application(s)?[select all that apply]	checkbox, Required<br>1 sub3a_d_1___1 parks<br>2 sub3a_d_1___2 picnic areas<br>3 sub3a_d_1___3 community centers<br>4 sub3a_d_1___4 school grounds<br>5 sub3a_d_1___5 forested areas<br>6 sub3a_d_1___6 campgrounds<br>7 sub3a_d_1___7 summer camps<br>8 sub3a_d_1___8 golf course<br>9 sub3a_d_1___9 other
	87	[sub3a_d_1_a]<br>Show the field ONLY if:<br>[sub3a_d_1(9)] = '1'	Please describe any other type(s) of public property your agency targets for acaricide applications:	text

	88	[sub3a_e]<br><br>Show the field ONLY if:<br>[sub3_c_methods_used(1)] = '1'	How does your agency evaluate the efficacy of acaricide applications?[select all that apply]	checkbox, Required<br>1 sub3a_e___1 reduction of tick density based on surveillance (e.g. tick flagging / dragging)<br>2 sub3a_e___2 reduction in ticks submitted by the public<br>3 sub3a_e___3 reduction in public complaints or service requests<br>4 sub3a_e___4 reduction in human tickborne disease cases<br>5 sub3a_e___5 other evaluation metric<br>6 sub3a_e___6 unfamiliar with evaluation methods<br>7 sub3a_e___7 no evaluation
	89	[sub3a_e_1]<br><br>Show the field ONLY if:<br>[sub3a_e(5)] = '1'	Please describe any other ways your agency evaluates acaricide applications:	text
	90	[sub3a_f]<br><br>Show the field ONLY if:<br>[sub3_c_methods_used(1)] = '1'	Do you want to expand your agency's capacity to apply acaricides on private property?	yesno, Required<br>1 Yes<br>0 No
	91	[sub3a_f_1_header]<br><br>Show the field ONLY if:<br>[sub3a_f] = '1'	What would be most helpful to expand your agency's capacity to apply acaricides on private property?<br><br>Please rank below choices in order of priority, with highest priority as '1' and lowest priority as '5':	descriptive
	92	[sub3a_f_1a]<br><br>Show the field ONLY if:<br>[sub3a_f] = '1'	funding	radio (Matrix - ranking), Required<br>1 1<br>2 2<br>3 3<br>4 4<br>5 5

	93	[sub3a_f_1b]<br><br>Show the field ONLY if:<br>[sub3a_f] = '1'	personnel	radio (Matrix - ranking), Required<br>1 1<br>2 2<br>3 3<br>4 4<br>5 5
	94	[sub3a_f_1c]<br><br>Show the field ONLY if:<br>[sub3a_f] = '1'	equipment	radio (Matrix - ranking), Required<br>1 1<br>2 2<br>3 3<br>4 4<br>5 5
	95	[sub3a_f_1d]<br><br>Show the field ONLY if:<br>[sub3a_f] = '1'	standardized protocols	radio (Matrix - ranking), Required<br>1 1<br>2 2<br>3 3<br>4 4<br>5 5
	96	[sub3a_f_1e]<br><br>Show the field ONLY if:<br>[sub3a_f] = '1'	training	radio (Matrix - ranking), Required<br>1 1<br>2 2<br>3 3<br>4 4<br>5 5
	97	[sub3a_f_1_a]<br><br>Show the field ONLY if:<br>[sub3a_f] = '1'	Please describe anything else you might require to help expand your agency's capacity to apply acaricides on private property:	text

	98	[sub3a_j_1]<br><br>Show the field ONLY if:<br>[sub3a_f] = '1'	Please select any other potential roadblocks to the development or expansion of your agency's capacity to apply acaricides to private property.[select all that apply]	checkbox, Required<br>1 | sub3a_j_1___1 | constrained by legislative mandate<br>2 | sub3a_j_1___2 | concerns for safety of staff<br>3 | sub3a_j_1___3 | public perceptions of environmental concerns<br>4 | sub3a_j_1___4 | public perceptions of personal/family health concerns<br>5 | sub3a_j_1___5 | limited evidence of control efficacy<br>6 | sub3a_j_1___6 | lack of administrative support<br>7 | sub3a_j_1___7 | public not likely to use information<br>8 | sub3a_j_1___8 | high risk areas protected under federal and state regulation<br>9 | sub3a_j_1___9 | local / state / federal agency concern with effects on wildlife population / health<br>10 | sub3a_j_1___10 | other
	99	[sub3a_j_1_a]<br><br>Show the field ONLY if:<br>[sub3a_j_1(10)] = '1'	Please describe any other potential roadblocks to the development or expansion of your agency's capacity to apply acaricides to private property:	text
	100	[sub3a_g]<br><br>Show the field ONLY if:<br>[sub3_c_methods_used(1)] = '1'	Do you want to expand your agency's capacity to apply acaricides on public property?	yesno, Required<br>1 | Yes<br>0 | No
	101	[sub3a_g_1_header]<br><br>Show the field ONLY if:<br>[sub3a_g] = '1'	What would be most helpful to expand your agency's capacity to apply acaricides on public property?<br><br>Please rank below choices in order of priority, with highest priority as '1' and lowest priority as '5':	descriptive

	102	[sub3a_g_1a]<br><br>Show the field ONLY if:<br>[sub3a_g] = '1'	funding	radio (Matrix - ranking), Required<br>1 1<br>2 2<br>3 3<br>4 4<br>5 5
	103	[sub3a_g_1b]<br><br>Show the field ONLY if:<br>[sub3a_g] = '1'	personnel	radio (Matrix - ranking), Required<br>1 1<br>2 2<br>3 3<br>4 4<br>5 5
	104	[sub3a_g_1c]<br><br>Show the field ONLY if:<br>[sub3a_g] = '1'	equipment	radio (Matrix - ranking), Required<br>1 1<br>2 2<br>3 3<br>4 4<br>5 5
	105	[sub3a_g_1d]<br><br>Show the field ONLY if:<br>[sub3a_g] = '1'	standardized protocols	radio (Matrix - ranking), Required<br>1 1<br>2 2<br>3 3<br>4 4<br>5 5

	106	[sub3a_g_1e]<br><br>Show the field ONLY if:<br>[sub3a_g] = '1'	training	radio (Matrix - ranking), Required<br>1 | 1<br>2 | 2<br>3 | 3<br>4 | 4<br>5 | 5
	107	[sub3a_g_1_a]<br><br>Show the field ONLY if:<br>[sub3a_g] = '1'	Please describe anything else you might require to help expand your agency's capacity to apply acaricides on public property:	text
	108	[sub3a_k_1]<br><br>Show the field ONLY if:<br>[sub3a_g] = '1'	Please select any other potential roadblocks to the development or expansion of your agency's capacity to apply acaricides to public property.[select all that apply]	checkbox, Required<br>1 | sub3a_k_1___1 | constrained by legislative mandate<br>2 | sub3a_k_1___2 | concerns for safety of staff<br>3 | sub3a_k_1___3 | public perceptions of environmental concerns<br>4 | sub3a_k_1___4 | public perceptions of personal/family health concerns<br>5 | sub3a_k_1___5 | limited evidence of control efficacy<br>6 | sub3a_k_1___6 | lack of administrative support<br>7 | sub3a_k_1___7 | public not likely to use information<br>8 | sub3a_k_1___8 | high risk areas protected under federal and state regulation<br>9 | sub3a_k_1___9 | local / state / federal agency concern with effects on wildlife population / health<br>10 | sub3a_k_1___10 | other

	109	[sub3a_k_1_a]<br>Show the field ONLY if:<br>[sub3a_k_1(10)] = '1'	Please describe any other potential roadblocks to the development or expansion of your agency's capacity to apply acaricides to private property:	text
	110	[sub3a_h]<br>Show the field ONLY if:<br>[sub3_c_methods_used(1)] = '0'	If resources, including funding and training opportunities, were available, would your agency be interested in developing the capacity to apply acaricides to control ticks?	yesno, Required<br>1 Yes<br>0 No
	111	[sub3a_h_1]<br>Show the field ONLY if:<br>[sub3a_h] = '1'	If your agency were to apply acaricides for tick control, what type(s) of public property would be most feasible for your agency to target?[select all that apply]	checkbox, Required<br>1 sub3a_h_1___1 parks<br>2 sub3a_h_1___2 picnic areas<br>3 sub3a_h_1___3 community centers<br>4 sub3a_h_1___4 school grounds<br>5 sub3a_h_1___5 forested areas<br>6 sub3a_h_1___6 campgrounds<br>7 sub3a_h_1___7 summer camps<br>8 sub3a_h_1___8 golf course<br>9 sub3a_h_1___9 other
	112	[sub3a_h_1_a]<br>Show the field ONLY if:<br>[sub3a_h_1(9)] = '1'	Please describe any other type(s) of public property your agency might target for acaricide applications:	text
	113	[sub3a_h_2]<br>Show the field ONLY if:<br>[sub3a_h] = '1'	If your agency were to apply acaricides, would it treat private property?	yesno, Required<br>1 Yes<br>0 No
	114	[sub3a_h_3_header]<br>Show the field ONLY if:<br>[sub3a_h] = '1'	What would be most helpful to expand your agency's capacity to apply acaricides?<br><br>Please rank below choices in order of priority, with highest priority as '1' and lowest priority as '5':	descriptive, Required

	115	[sub3a_h_3a]<br>Show the field ONLY if:<br>[sub3a_h] = '1'	funding	radio (Matrix - ranking), Required<br>1 1<br>2 2<br>3 3<br>4 4<br>5 5
	116	[sub3a_h_3b]<br>Show the field ONLY if:<br>[sub3a_h] = '1'	personnel	radio (Matrix - ranking), Required<br>1 1<br>2 2<br>3 3<br>4 4<br>5 5
	117	[sub3a_h_3c]<br>Show the field ONLY if:<br>[sub3a_h] = '1'	equipment	radio (Matrix - ranking), Required<br>1 1<br>2 2<br>3 3<br>4 4<br>5 5
	118	[sub3a_h_3d]<br>Show the field ONLY if:<br>[sub3a_h] = '1'	standardized protocols	radio (Matrix - ranking), Required<br>1 1<br>2 2<br>3 3<br>4 4<br>5 5

	119	[sub3a_h_3e]<br><br>Show the field ONLY if:<br>[sub3a_h] = '1'	training	radio (Matrix - ranking), Required<br>1 | 1<br>2 | 2<br>3 | 3<br>4 | 4<br>5 | 5
	120	[sub3a_h_3_a]<br><br>Show the field ONLY if:<br>[sub3a_h] = '1'	Please describe anything else you might require to help expand your agency's capacity to apply acaricides:	text
	121	[sub3a_l_1]<br><br>Show the field ONLY if:<br>[sub3a_h] = '1'	Please select any other potential roadblocks to the development or expansion of your agency's capacity to apply acaricides.[select all that apply]	checkbox, Required<br>1 | sub3a_l_1___1 | constrained by legislative mandate<br>2 | sub3a_l_1___2 | concerns for safety of staff<br>3 | sub3a_l_1___3 | public perceptions of environmental concerns<br>4 | sub3a_l_1___4 | public perceptions of personal/family health concerns<br>5 | sub3a_l_1___5 | limited evidence of control efficacy<br>6 | sub3a_l_1___6 | lack of administrative support<br>7 | sub3a_l_1___7 | public not likely to use information<br>8 | sub3a_l_1___8 | high risk areas protected under federal and state regulation<br>9 | sub3a_l_1___9 | local / state / federal agency concern with effects on wildlife population / health<br>10 | sub3a_l_1___10 | other

	122	[sub3a_l_1_a]<br><br>Show the field ONLY if:<br>[sub3a_l_1(10)] = '1'	Please describe any other potential roadblocks to the development or expansion of your agency's capacity to apply acaricides to private property:	text
	123	[sub3a_h_4]<br><br>Show the field ONLY if:<br>[sub3a_h] = '0'	Why is your agency uninterested in applying acaricides? [select all that apply]	checkbox, Required<br>1 | sub3a_h_4___1 | ticks and tickborne disease are not a priority concern in our jurisdiction<br>2 | sub3a_h_4___2 | constrained by legislative mandate<br>3 | sub3a_h_4___3 | concerns for safety of staff<br>4 | sub3a_h_4___4 | public perceptions of environmental concerns<br>5 | sub3a_h_4___5 | public perceptions of personal/family health concerns<br>6 | sub3a_h_4___6 | limited evidence of control efficacy<br>7 | sub3a_h_4___7 | lack of administrative support<br>8 | sub3a_h_4___8 | lack of trained personnel<br>9 | sub3a_h_4___9 | local / state / federal agency concern with effects on wildlife population / health<br>10 | sub3a_h_4___10 | other
	124	[sub3a_h_4_a]<br><br>Show the field ONLY if:<br>[sub3a_h_4(10)] = '1'	Please describe any other reason(s) your agency is uninterested in applying acaricides:	text
	125	[subsection_3a_acaricide_application_complete]	Section Header: *Form Status*<br>Complete?	dropdown<br>0 | Incomplete<br>1 | Unverified<br>2 | Complete

Instrument: **Subsection 3c: Rodent targeted intervention** (subsection_3c_rodent_targeted_intervention) Enabled as survey

	126	[sub3c_i]<br><br>Show the field ONLY if:<br>[sub3_c_methods_used(3)] = '1'	Section Header: *Capacity to deploy rodent-targeted tick control*<br><br>What rodent-targeted tick control methods does your agency deploy?[select all that apply]	checkbox, Required<br>1 sub3c_i___1 damminix tick tubes<br>2 sub3c_i___2 thermacell tick tubes<br>3 sub3c_i___3 select TCS bait boxes<br>4 sub3c_i___4 other
	127	[sub3c_i_1]<br><br>Show the field ONLY if:<br>[sub3c_i(4)] = '1'	Please describe any other rodent-targeted tick control method(s) your agency deploys:	text
	128	[sub3c_a]<br><br>Show the field ONLY if:<br>[sub3_c_methods_used(3)] = '1'	What are the funding sources for your agency's rodent-targeted tick control activities?[select all that apply]	checkbox, Required<br>1 sub3c_a___1 local property taxes<br>2 sub3c_a___2 state taxes<br>3 sub3c_a___3 county taxes<br>4 sub3c_a___4 town or city taxes<br>5 sub3c_a___5 private donations<br>6 sub3c_a___6 surcharge on services or goods<br>7 sub3c_a___7 federal funds<br>8 sub3c_a___8 other
	129	[sub3c_a_1]<br><br>Show the field ONLY if:<br>[sub3c_a(8)] = '1'	Please describe any other funding source(s) your agency uses for rodent-targeted tick control:	text
	130	[sub3c_d]<br><br>Show the field ONLY if:<br>[sub3_c_methods_used(3)] = '1'	Does your agency target private property, public property, or both, when deploying rodent-targeted tick control?	radio, Required<br>1 private properties<br>2 public properties<br>3 both private and public properties

	131	[sub3c_b]<br><br>Show the field ONLY if:<br>[sub3c_d] = '3' or [sub3c_d] = '2'	What triggers your agency to deploy rodent-targeted tick control on public property?[select all that apply]	checkbox, Required<br>1 sub3c_b___1 in response to tick surveillance data<br>2 sub3c_b___2 in response to human case data<br>3 sub3c_b___3 ongoing program deployed at predetermined sites<br>4 sub3c_b___4 based on public complaint or request<br>5 sub3c_b___5 other
	132	[sub3c_b_1]<br><br>Show the field ONLY if:<br>[sub3c_b(5)] = '1'	Please describe any other reason(s) that trigger your agency to deploy rodent-targeted tick control on public property:	text
	133	[sub3c_d_2]<br><br>Show the field ONLY if:<br>[sub3c_d] = '3' or [sub3c_d] = '1'	What triggers your agency to deploy rodent-targeted tick control on private property?[select all that apply]	checkbox, Required<br>1 sub3c_d_2___1 in response to tick surveillance data<br>2 sub3c_d_2___2 in response to human case data<br>3 sub3c_d_2___3 ongoing program deployed at predetermined sites<br>4 sub3c_d_2___4 landowner request<br>5 sub3c_d_2___5 other
	134	[sub3c_d_2_1]<br><br>Show the field ONLY if:<br>[sub3c_d_2(5)] = '1'	Please describe any other reason(s) that trigger your agency to deploy rodent-targeted tick control on private property:	text
	135	[sub3c_c]<br><br>Show the field ONLY if:<br>[sub3c_d] = '2' or [sub3c_d] = '3'	At approximately how many public properties does your agency deploy rodent-targeted tick control per year?	radio, Required<br>1 1-5<br>2 5-10<br>3 10-50<br>4 >50

	136	[sub3c_c_2]<br><br>Show the field ONLY if:<br>[sub3c_d] = '3' or [sub3c_d] = '1'	At approximately how many private properties does your agency deploy rodent-targeted tick control per year?	radio, Required<br>1 1-5<br>2 5-10<br>3 10-50<br>4 >50
	137	[sub3c_d_1]<br><br>Show the field ONLY if:<br>[sub3c_d] = '2' or [sub3c_d] = '3'	What type(s) of public property does your agency target when using rodent-targeted tick control?[select all that apply]	checkbox, Required<br>1 sub3c_d_1___1 parks<br>2 sub3c_d_1___2 picnic areas<br>3 sub3c_d_1___3 community centers<br>4 sub3c_d_1___4 school grounds<br>5 sub3c_d_1___5 forested areas<br>6 sub3c_d_1___6 campgrounds<br>7 sub3c_d_1___7 summer camps<br>8 sub3c_d_1___8 golf course<br>9 sub3c_d_1___9 other
	138	[sub3c_d_1_a]<br><br>Show the field ONLY if:<br>[sub3c_d_1(9)] = '1'	Please describe any other type(s) of public property your agency targets with rodent-targeted tick control:	text

	139	[sub3c_e]<br><br>Show the field ONLY if:<br>[sub3_c_methods_used(3)] = '1'	How does your agency evaluate the efficacy of rodent-targeted tick control?[select all that apply]	checkbox, Required<br>1 | sub3c_e___1 | reduction of tick density based on surveillance (e.g. tick flagging / dragging)<br>2 | sub3c_e___2 | reduction in ticks submitted by the public<br>3 | sub3c_e___3 | reduction in public complaints or service requests<br>4 | sub3c_e___4 | reduction in human tickborne disease cases<br>5 | sub3c_e___5 | other evaluation metric<br>6 | sub3c_e___6 | unfamiliar with evaluation methods<br>7 | sub3c_e___7 | no evaluation
	140	[sub3c_e_1]<br><br>Show the field ONLY if:<br>[sub3c_e(5)] = '1'	Please describe any other way(s) your agency evaluates the efficacy of rodent-targeted tick control:	text
	141	[sub3c_f]<br><br>Show the field ONLY if:<br>[sub3_c_methods_used(3)] = '1'	Do you want to expand your agency's capacity to deploy rodent-targeted tick control on private property?	yesno, Required<br>1 | Yes<br>0 | No
	142	[sub3c_f_1_header]<br><br>Show the field ONLY if:<br>[sub3c_f] = '1'	What would be most helpful to expand your agency's capacity to deploy rodent-targeted tick control on private property?<br><br>Please rank below choices in order of priority, with highest priority as '1' and lowest priority as '5':	descriptive
	143	[sub3c_f_1a]<br><br>Show the field ONLY if:<br>[sub3c_f] = '1'	funding	radio (Matrix - ranking), Required<br>1 | 1<br>2 | 2<br>3 | 3<br>4 | 4<br>5 | 5

	144	[sub3c_f_1b]<br>Show the field ONLY if:<br>[sub3c_f] = '1'	personnel	radio (Matrix - ranking), Required<br>1 1<br>2 2<br>3 3<br>4 4<br>5 5
	145	[sub3c_f_1c]<br>Show the field ONLY if:<br>[sub3c_f] = '1'	equipment	radio (Matrix - ranking), Required<br>1 1<br>2 2<br>3 3<br>4 4<br>5 5
	146	[sub3c_f_1d]<br>Show the field ONLY if:<br>[sub3c_f] = '1'	standardized protocols	radio (Matrix - ranking), Required<br>1 1<br>2 2<br>3 3<br>4 4<br>5 5
	147	[sub3c_f_1e]<br>Show the field ONLY if:<br>[sub3c_f] = '1'	training	radio (Matrix - ranking), Required<br>1 1<br>2 2<br>3 3<br>4 4<br>5 5
	148	[sub3c_f_1_a]<br>Show the field ONLY if:<br>[sub3c_f] = '1'	Please describe anything else you might require to help expand your agency's capacity to deploy rodent-targeted tick control on private property:	text

<table>
<tr><td></td><td>149</td><td>[sub3c_j_1]

Show the field ONLY if:
[sub3c_f] = '1'</td><td>Please select any other potential roadblocks to the development or expansion of your agency's capacity to deploy rodent-targeted tick control on private property. [select all that apply]</td><td>checkbox, Required<table><tr><td>1</td><td>sub3c_j_1___1</td><td>constrained by legislative mandate</td></tr><tr><td>2</td><td>sub3c_j_1___2</td><td>concerns for safety of staff</td></tr><tr><td>3</td><td>sub3c_j_1___3</td><td>public perceptions of environmental concerns</td></tr><tr><td>4</td><td>sub3c_j_1___4</td><td>public perceptions of personal/family health concerns</td></tr><tr><td>5</td><td>sub3c_j_1___5</td><td>limited evidence of control efficacy</td></tr><tr><td>6</td><td>sub3c_j_1___6</td><td>lack of administrative support</td></tr><tr><td>7</td><td>sub3c_j_1___7</td><td>public not likely to use information</td></tr><tr><td>8</td><td>sub3c_j_1___8</td><td>high risk areas protected under federal and state regulation</td></tr><tr><td>9</td><td>sub3c_j_1___9</td><td>local / state / federal agency concern with effects on wildlife population / health</td></tr><tr><td>10</td><td>sub3c_j_1___10</td><td>other</td></tr></table></td></tr>
<tr><td></td><td>150</td><td>[sub3c_j_1_a]

Show the field ONLY if:
[sub3c_j_1(10)] = '1'</td><td>Please describe any other potential roadblocks to the development or expansion of your agency's capacity to deploy rodent-targeted tick control on private property:</td><td>text</td></tr>
<tr><td></td><td>151</td><td>[sub3c_g]

Show the field ONLY if:
[sub3_c_methods_used(3)] = '1'</td><td>Do you want to expand your agency's capacity to deploy rodent-targeted tick control on public property?</td><td>yesno, Required<table><tr><td>1</td><td>Yes</td></tr><tr><td>0</td><td>No</td></tr></table></td></tr>
<tr><td></td><td>152</td><td>[sub3c_g_1_header]

Show the field ONLY if:
[sub3c_g] = '1'</td><td>What would be most helpful to expand your agency's capacity to deploy rodent-targeted tick control on public property?

Please rank below choices in order of priority, with highest priority as '1' and lowest priority as '5':</td><td>descriptive</td></tr>
</table>

	153	[sub3c_g_1a]<br><br>Show the field ONLY if:<br>[sub3c_g] = '1'	funding	radio (Matrix - ranking), Required<br>1 1<br>2 2<br>3 3<br>4 4<br>5 5
	154	[sub3c_g_1b]<br><br>Show the field ONLY if:<br>[sub3c_g] = '1'	personnel	radio (Matrix - ranking), Required<br>1 1<br>2 2<br>3 3<br>4 4<br>5 5
	155	[sub3c_g_1c]<br><br>Show the field ONLY if:<br>[sub3c_g] = '1'	equipment	radio (Matrix - ranking), Required<br>1 1<br>2 2<br>3 3<br>4 4<br>5 5
	156	[sub3c_g_1d]<br><br>Show the field ONLY if:<br>[sub3c_g] = '1'	standardized protocols	radio (Matrix - ranking), Required<br>1 1<br>2 2<br>3 3<br>4 4<br>5 5

	157	[sub3c_g_1e]<br>Show the field ONLY if:<br>[sub3c_g] = '1'	training	radio (Matrix - ranking), Required<br>1 | 1<br>2 | 2<br>3 | 3<br>4 | 4<br>5 | 5
	158	[sub3c_g_1_a]<br>Show the field ONLY if:<br>[sub3c_g] = '1'	Please describe anything else you might require to help expand your agency's capacity to deploy rodent-targeted tick control on public property:	text
	159	[sub3c_k_1]<br>Show the field ONLY if:<br>[sub3c_g] = '1'	Please select any other potential roadblocks to the development or expansion of your agency's capacity to deploy rodent-targeted tick control on public property. [select all that apply]	checkbox, Required<br>1 | sub3c_k_1___1 | constrained by legislative mandate<br>2 | sub3c_k_1___2 | concerns for safety of staff<br>3 | sub3c_k_1___3 | public perceptions of environmental concerns<br>4 | sub3c_k_1___4 | public perceptions of personal/family health concerns<br>5 | sub3c_k_1___5 | limited evidence of control efficacy<br>6 | sub3c_k_1___6 | lack of administrative support<br>7 | sub3c_k_1___7 | public not likely to use information<br>8 | sub3c_k_1___8 | high risk areas protected under federal and state regulation<br>9 | sub3c_k_1___9 | local / state / federal agency concern with effects on wildlife population / health<br>10 | sub3c_k_1___10 | other

	160	[sub3c_k_1_a]<br><br>Show the field ONLY if:<br>[sub3c_k_1(10)] = '1'	Please describe any other potential roadblocks to the development or expansion of your agency's capacity to deploy rodent-targeted tick control on public property:	text
	161	[sub3c_h]<br><br>Show the field ONLY if:<br>[sub3_c_methods_used(3)] = '0'	If resources, including funding and training opportunities, were available, would your agency be interested in developing the capacity to deploy rodent-targeted tick control?	yesno, Required<br>1 Yes<br>0 No
	162	[sub3c_h_1]<br><br>Show the field ONLY if:<br>[sub3c_h] = '1'	If your agency were to deploy rodent-targeted tick control, what type(s) of public property would be most feasible for your agency to target?[select all that apply]	checkbox, Required<br>1 sub3c_h_1___1 parks<br>2 sub3c_h_1___2 picnic areas<br>3 sub3c_h_1___3 community centers<br>4 sub3c_h_1___4 school grounds<br>5 sub3c_h_1___5 forested areas<br>6 sub3c_h_1___6 campgrounds<br>7 sub3c_h_1___7 summer camps<br>8 sub3c_h_1___8 golf course<br>9 sub3c_h_1___9 other
	163	[sub3c_h_1_a]<br><br>Show the field ONLY if:<br>[sub3c_h_1(9)] = '1'	Please describe any other type(s) of public property your agency might target:	text
	164	[sub3c_h_2]<br><br>Show the field ONLY if:<br>[sub3c_h] = '1'	If your agency were to deploy rodent-targeted tick control, would it treat private property?	yesno, Required<br>1 Yes<br>0 No
	165	[sub3c_h_3_header]<br><br>Show the field ONLY if:<br>[sub3c_h] = '1'	What would be most helpful to expand your agency's capacity to deploy rodent-targeted tick control?<br><br>Please rank below choices in order of priority, with highest priority as '1' and lowest priority as '5':	descriptive

	166	[sub3c_h_3a]<br>Show the field ONLY if:<br>[sub3c_h] = '1'	funding	radio (Matrix - ranking), Required<br>1 1<br>2 2<br>3 3<br>4 4<br>5 5
	167	[sub3c_h_3b]<br>Show the field ONLY if:<br>[sub3c_h] = '1'	personnel	radio (Matrix - ranking), Required<br>1 1<br>2 2<br>3 3<br>4 4<br>5 5
	168	[sub3c_h_3c]<br>Show the field ONLY if:<br>[sub3c_h] = '1'	equipment	radio (Matrix - ranking), Required<br>1 1<br>2 2<br>3 3<br>4 4<br>5 5
	169	[sub3c_h_3d]<br>Show the field ONLY if:<br>[sub3c_h] = '1'	standardized protocols	radio (Matrix - ranking), Required<br>1 1<br>2 2<br>3 3<br>4 4<br>5 5

<table>
<tr><td></td><td>170</td><td>[sub3c_h_3e]

Show the field ONLY if:
[sub3c_h] = '1'</td><td>training</td><td>radio (Matrix - ranking), Required<table><tr><td>1</td><td>1</td></tr><tr><td>2</td><td>2</td></tr><tr><td>3</td><td>3</td></tr><tr><td>4</td><td>4</td></tr><tr><td>5</td><td>5</td></tr></table></td></tr>
<tr><td></td><td>171</td><td>[sub3c_h_3_a]

Show the field ONLY if:
[sub3c_h] = '1'</td><td>Please describe anything else you might require to help expand your agency's capacity to deploy rodent-targeted tick control:</td><td>text</td></tr>
<tr><td></td><td>172</td><td>[sub3c_l_1]

Show the field ONLY if:
[sub3c_h] = '1'</td><td>Please select any other potential roadblocks to the development or expansion of your agency's capacity to deploy rodent-targeted tick control.[select all that apply]</td><td>checkbox, Required<table>
<tr><td>1</td><td>sub3c_l_1___1</td><td>constrained by legislative mandate</td></tr>
<tr><td>2</td><td>sub3c_l_1___2</td><td>concerns for safety of staff</td></tr>
<tr><td>3</td><td>sub3c_l_1___3</td><td>public perceptions of environmental concerns</td></tr>
<tr><td>4</td><td>sub3c_l_1___4</td><td>public perceptions of personal/family health concerns</td></tr>
<tr><td>5</td><td>sub3c_l_1___5</td><td>limited evidence of control efficacy</td></tr>
<tr><td>6</td><td>sub3c_l_1___6</td><td>lack of administrative support</td></tr>
<tr><td>7</td><td>sub3c_l_1___7</td><td>public not likely to use information</td></tr>
<tr><td>8</td><td>sub3c_l_1___8</td><td>high risk areas protected under federal and state regulation</td></tr>
<tr><td>9</td><td>sub3c_l_1___9</td><td>local / state / federal agency concern with effects on wildlife population / health</td></tr>
<tr><td>10</td><td>sub3c_l_1___10</td><td>other</td></tr>
</table></td></tr>
</table>

	173	[sub3c_l_1_a]<br><br>Show the field ONLY if:<br>[sub3c_l_1(10)] = '1'	Please describe any other potential roadblocks to the development or expansion of your agency's capacity to deploy rodent-targeted tick control:	text
	174	[sub3c_h_4]<br><br>Show the field ONLY if:<br>[sub3c_h] = '0'	Why is your agency uninterested in deploying rodent-targeted tick control?[select all that apply]	checkbox, Required<br>1 | sub3c_h_4___1 | ticks and tickborne disease are not a priority concern in our jurisdiction<br>2 | sub3c_h_4___2 | constrained by legislative mandate<br>3 | sub3c_h_4___3 | concerns for safety of staff<br>4 | sub3c_h_4___4 | local / state / federal agency concern with effects on wildlife population / health<br>5 | sub3c_h_4___5 | public perceptions of environmental concerns<br>6 | sub3c_h_4___6 | public perceptions of personal/family health concerns<br>7 | sub3c_h_4___7 | limited evidence of control efficacy<br>8 | sub3c_h_4___8 | lack of administrative support<br>9 | sub3c_h_4___9 | lack of trained personnel<br>10 | sub3c_h_4___10 | other
	175	[sub3c_h_4_a]<br><br>Show the field ONLY if:<br>[sub3c_h_4(10)] = '1'	Please describe any other reason(s) your agency is uninterested in deploying rodent-targeted tick control:	text
	176	[subsection_3c_rodent_targeted_intervention_complete]	Section Header: *Form Status*<br><br>Complete?	dropdown<br>0 | Incomplete<br>1 | Unverified<br>2 | Complete

Instrument: **Subsection 3d: Habitat or landscape management** (subsection_3d_habitat_or_landscape_management) Enabled as survey

	177	[sub3d_i]<br><br>Show the field ONLY if:<br>[sub3_c_methods_used(4)] = '1'	Section Header: *Capacity to deploy habitat or landscape modification tick control*<br><br>What habitat or landscape modification tick control methods does your agency deploy?[select all that apply]	checkbox, Required<br>1 sub3d_i___1 mowing<br>2 sub3d_i___2 brush clearing<br>3 sub3d_i___3 leaf litter removal<br>4 sub3d_i___4 xeric borders along high-risk tick habitat<br>5 sub3d_i___5 burning<br>6 sub3d_i___6 other
	178	[sub3d_i_1]<br><br>Show the field ONLY if:<br>[sub3d_i(6)] = '1'	Please describe any other habitat or landscape modification tick control method(s) your agency deploys:	text
	179	[sub3d_a]<br><br>Show the field ONLY if:<br>[sub3_c_methods_used(4)] = '1'	What are the funding sources for your agency's habitat or landscape management tick control? [select all that apply]	checkbox, Required<br>1 sub3d_a___1 local property taxes<br>2 sub3d_a___2 state taxes<br>3 sub3d_a___3 county taxes<br>4 sub3d_a___4 town or city taxes<br>5 sub3d_a___5 private donations<br>6 sub3d_a___6 surcharge on services or goods<br>7 sub3d_a___7 federal funds<br>8 sub3d_a___8 other
	180	[sub3d_a_1]<br><br>Show the field ONLY if:<br>[sub3d_a(8)] = '1'	Please describe any other funding source(s) for your agency's habitat or landscape management tick control:	text
	181	[sub3d_d]<br><br>Show the field ONLY if:<br>[sub3_c_methods_used(4)] = '1'	Does your agency deploy habitat or landscape management tick control on private or public properties, or both?	radio, Required<br>1 private properties<br>2 public properties<br>3 both private and public properties

	182	[sub3d_b]<br>Show the field ONLY if:<br>[sub3d_d] = '2' or [sub3d_d] = '3'	What triggers your agency to deploy habitat or landscape management tick control on public property? [select all that apply]	checkbox, Required<br>1 | sub3d_b___1 | in response to tick surveillance data<br>2 | sub3d_b___2 | in response to human case data<br>3 | sub3d_b___3 | ongoing program deployed at predetermined sites<br>4 | sub3d_b___4 | based on public complaint or request<br>5 | sub3d_b___5 | other
	183	[sub3d_b_1]<br>Show the field ONLY if:<br>[sub3d_b(5)] = '1'	Please describe any other reason(s) that trigger your agency to deploy habitat or landscape management tick control on public property:	text
	184	[sub3d_d_2]<br>Show the field ONLY if:<br>[sub3d_d] = '3' or [sub3d_d] = '1'	What triggers your agency to deploy habitat or landscape management tick control on private property? [select all that apply]	checkbox, Required<br>1 | sub3d_d_2___1 | in response to tick surveillance data<br>2 | sub3d_d_2___2 | in response to human case data<br>3 | sub3d_d_2___3 | ongoing program deployed at predetermined sites<br>4 | sub3d_d_2___4 | landowner request<br>5 | sub3d_d_2___5 | other
	185	[sub3d_d_2_1]<br>Show the field ONLY if:<br>[sub3d_d_2(5)] = '1'	Please describe any other reason(s) that trigger your agency to deploy habitat or landscape management tick control on private property:	text
	186	[sub3d_c]<br>Show the field ONLY if:<br>[sub3d_d] = '2' or [sub3d_d] = '3'	On approximately how many public properties does your agency deploy habitat or landscape management tick control per year?	radio, Required<br>1 | 1-5<br>2 | 5-10<br>3 | 10-50<br>4 | >50

<table>
<tr><td></td><td>187</td><td>[sub3d_c_2]

Show the field ONLY if:
[sub3d_d] = '3' or [sub3d_d] = '1'</td><td>On approximately how many private properties does your agency deploy habitat or landscape management tick control per year?</td><td>radio, Required<table><tr><td>1</td><td>1-5</td></tr><tr><td>2</td><td>5-10</td></tr><tr><td>3</td><td>10-50</td></tr><tr><td>4</td><td>>50</td></tr></table></td></tr>
<tr><td></td><td>188</td><td>[sub3d_d_1]

Show the field ONLY if:
[sub3d_d] = '2' or [sub3d_d] = '3'</td><td>On what type(s) of public space(s) does your agency deploy habitat or landscape management tick control? [select all that apply]</td><td>checkbox, Required<table><tr><td>1</td><td>sub3d_d_1___1</td><td>parks</td></tr><tr><td>2</td><td>sub3d_d_1___2</td><td>picnic areas</td></tr><tr><td>3</td><td>sub3d_d_1___3</td><td>community centers</td></tr><tr><td>4</td><td>sub3d_d_1___4</td><td>school grounds</td></tr><tr><td>5</td><td>sub3d_d_1___5</td><td>forested areas</td></tr><tr><td>6</td><td>sub3d_d_1___6</td><td>campgrounds</td></tr><tr><td>7</td><td>sub3d_d_1___7</td><td>summer camps</td></tr><tr><td>8</td><td>sub3d_d_1___8</td><td>golf course</td></tr><tr><td>9</td><td>sub3d_d_1___9</td><td>other</td></tr></table></td></tr>
<tr><td></td><td>189</td><td>[sub3d_d_1_a]

Show the field ONLY if:
[sub3d_d_1(9)] = '1'</td><td>Please describe any other type(s) of public spaces on which your agency deploys habitat or landscape management tick control:</td><td>text</td></tr>
</table>

	190	[sub3d_e]<br><br>Show the field ONLY if:<br>[sub3_c_methods_used(4)] = '1'	How does your agency evaluate the efficacy of habitat or landscape management tick control? [select all that apply]	checkbox, Required<br>1 sub3d_e___1 reduction of tick density based on surveillance (e.g. tick flagging / dragging)<br>2 sub3d_e___2 reduction in ticks submitted by the public<br>3 sub3d_e___3 reduction in public complaints or service requests<br>4 sub3d_e___4 reduction in human tickborne disease cases<br>5 sub3d_e___5 other evaluation metric<br>6 sub3d_e___6 unfamiliar with evaluation methods<br>7 sub3d_e___7 no evaluation
	191	[sub3d_e_1]<br><br>Show the field ONLY if:<br>[sub3d_e(5)] = '1'	Please describe any other way(s) in which your agency evaluates habitat or landscape management tick control:	text
	192	[sub3d_f]<br><br>Show the field ONLY if:<br>[sub3_c_methods_used(4)] = '1'	Do you want to expand your agency's capacity to deploy habitat or landscape management tick control on private properties?	yesno, Required<br>1 Yes<br>0 No
	193	[sub3d_f_1_header]<br><br>Show the field ONLY if:<br>[sub3d_f] = '1'	What would be most helpful to expand your agency's capacity to deploy habitat or landscape management tick control on private properties?<br><br>Please rank below choices in order of priority, with highest priority as '1' and lowest priority as '5':	descriptive

	194	[sub3d_f_1a]<br><br>Show the field ONLY if:<br>[sub3d_f] = '1'	funding	radio (Matrix - ranking), Required<br>1 1<br>2 2<br>3 3<br>4 4<br>5 5
	195	[sub3d_f_1b]<br><br>Show the field ONLY if:<br>[sub3d_f] = '1'	personnel	radio (Matrix - ranking), Required<br>1 1<br>2 2<br>3 3<br>4 4<br>5 5
	196	[sub3d_f_1c]<br><br>Show the field ONLY if:<br>[sub3d_f] = '1'	equipment	radio (Matrix - ranking), Required<br>1 1<br>2 2<br>3 3<br>4 4<br>5 5
	197	[sub3d_f_1d]<br><br>Show the field ONLY if:<br>[sub3d_f] = '1'	standardized protocols	radio (Matrix - ranking), Required<br>1 1<br>2 2<br>3 3<br>4 4<br>5 5

	198	[sub3d_f_1e]<br><br>Show the field ONLY if:<br>[sub3d_f] = '1'	training	radio (Matrix - ranking), Required<br>1 | 1<br>2 | 2<br>3 | 3<br>4 | 4<br>5 | 5
	199	[sub3d_f_1_a]<br><br>Show the field ONLY if:<br>[sub3d_f] = '1'	Please describe anything else you might require to help expand your agency's capacity to deploy habitat or landscape management tick control on private properties:	text
	200	[sub3d_j_1]<br><br>Show the field ONLY if:<br>[sub3d_f] = '1'	Please select any other potential roadblocks to the development or expansion of your agency's capacity to deploy habitat or landscape management tick control on private property.[select all that apply]	checkbox, Required<br>1 | sub3d_j_1___1 | constrained by legislative mandate<br>2 | sub3d_j_1___2 | concerns for safety of staff<br>3 | sub3d_j_1___3 | public perceptions of environmental concerns<br>4 | sub3d_j_1___4 | public perceptions of personal/family health concerns<br>5 | sub3d_j_1___5 | limited evidence of control efficacy<br>6 | sub3d_j_1___6 | lack of administrative support<br>7 | sub3d_j_1___7 | public not likely to use information<br>8 | sub3d_j_1___8 | high risk areas protected under federal and state regulation<br>9 | sub3d_j_1___9 | other
	201	[sub3d_j_1_a]<br><br>Show the field ONLY if:<br>[sub3d_j_1(9)] = '1'	Please describe any other potential roadblocks to the development or expansion of your agency's capacity to deploy habitat or landscape management tick control on private property:	text

	202	[sub3d_g]<br><br>Show the field ONLY if:<br>[sub3_c_methods_used(4)] = '1'	Do you want to expand your agency's capacity to deploy habitat or landscape management tick control on public property?	yesno, Required<br>1 Yes<br>0 No
	203	[sub3d_g_1_header]<br><br>Show the field ONLY if:<br>[sub3d_g] = '1'	What would be most helpful to expand your agency's capacity to use habitat or landscape management tick control methods on public properties or lands?<br><br>Please rank below choices in order of priority, with highest priority as '1' and lowest priority as '5':	descriptive
	204	[sub3d_g_1a]<br><br>Show the field ONLY if:<br>[sub3d_g] = '1'	funding	radio (Matrix - ranking), Required<br>1 1<br>2 2<br>3 3<br>4 4<br>5 5
	205	[sub3d_g_1b]<br><br>Show the field ONLY if:<br>[sub3d_g] = '1'	personnel	radio (Matrix - ranking), Required<br>1 1<br>2 2<br>3 3<br>4 4<br>5 5
	206	[sub3d_g_1c]<br><br>Show the field ONLY if:<br>[sub3d_g] = '1'	equipment	radio (Matrix - ranking), Required<br>1 1<br>2 2<br>3 3<br>4 4<br>5 5

	207	[sub3d_g_1d]<br><br>Show the field ONLY if:<br>[sub3d_g] = '1'	standardized protocols	radio (Matrix - ranking), Required<br>1 1<br>2 2<br>3 3<br>4 4<br>5 5
	208	[sub3d_g_1e]<br><br>Show the field ONLY if:<br>[sub3d_g] = '1'	training	radio (Matrix - ranking), Required<br>1 1<br>2 2<br>3 3<br>4 4<br>5 5
	209	[sub3d_g_1_a]<br><br>Show the field ONLY if:<br>[sub3d_g] = '1'	Please describe anything else that may help expand your agency's capacity to deploy habitat or landscape management tick control on public property:	text

<table>
<tr><td></td><td>210</td><td>[sub3d_k_1]

Show the field ONLY if:
[sub3d_g] = '1'</td><td>Please select any other potential roadblocks to the development or expansion of your agency's capacity to deploy habitat or landscape management tick control on public property.[select all that apply]</td><td>checkbox, Required
<table><tr><td>1</td><td>sub3d_k_1___1</td><td>constrained by legislative mandate</td></tr><tr><td>2</td><td>sub3d_k_1___2</td><td>concerns for safety of staff</td></tr><tr><td>3</td><td>sub3d_k_1___3</td><td>public perceptions of environmental concerns</td></tr><tr><td>4</td><td>sub3d_k_1___4</td><td>public perceptions of personal/family health concerns</td></tr><tr><td>5</td><td>sub3d_k_1___5</td><td>limited evidence of control efficacy</td></tr><tr><td>6</td><td>sub3d_k_1___6</td><td>lack of administrative support</td></tr><tr><td>7</td><td>sub3d_k_1___7</td><td>public not likely to use information</td></tr><tr><td>8</td><td>sub3d_k_1___8</td><td>high risk areas protected under federal and state regulation</td></tr><tr><td>9</td><td>sub3d_k_1___9</td><td>other</td></tr></table></td></tr>
<tr><td></td><td>211</td><td>[sub3d_k_1_a]

Show the field ONLY if:
[sub3d_k_1(9)] = '1'</td><td>Please describe any other potential roadblocks to the development or expansion of your agency's capacity to deploy habitat or landscape management tick control on public property:</td><td>text</td></tr>
<tr><td></td><td>212</td><td>[sub3d_h]

Show the field ONLY if:
[sub3_c_methods_used(4)] = '0'</td><td>If resources, including funding and training opportunities, were available, would your agency be interested in developing the capacity to deploy habitat or landscape management tick control?</td><td>yesno, Required
<table><tr><td>1</td><td>Yes</td></tr><tr><td>0</td><td>No</td></tr></table></td></tr>
</table>

	213	[sub3d_h_1]<br><br>Show the field ONLY if:<br>[sub3d_h] = '1'	If your agency were to deploy habitat or landscape management tick control, what type(s) of public space(s) would be most feasible for your agency to target?[select all that apply]	checkbox, Required<br>1 | sub3d_h_1___1 | parks<br>2 | sub3d_h_1___2 | picnic areas<br>3 | sub3d_h_1___3 | community centers<br>4 | sub3d_h_1___4 | school grounds<br>5 | sub3d_h_1___5 | forested areas<br>6 | sub3d_h_1___6 | campgrounds<br>7 | sub3d_h_1___7 | summer camps<br>8 | sub3d_h_1___8 | golf course<br>9 | sub3d_h_1___9 | other
	214	[sub3d_h_1_a]<br><br>Show the field ONLY if:<br>[sub3d_h_1(9)] = '1'	Please describe other type(s) of public space(s) your agency might target:	text
	215	[sub3d_h_2]<br><br>Show the field ONLY if:<br>[sub3d_h] = '1'	If your agency were to deploy habitat or landscape management tick control, would it target private properties?	yesno, Required<br>1 | Yes<br>0 | No
	216	[sub3d_h_3_header]<br><br>Show the field ONLY if:<br>[sub3d_h] = '1'	What would be most helpful to expand your agency's capacity to deploy habitat or landscape management tick control?<br><br>Please rank below choices in order of priority, with highest priority as '1' and lowest priority as '5':	descriptive
	217	[sub3d_h_3a]<br><br>Show the field ONLY if:<br>[sub3d_h] = '1'	funding	radio (Matrix - ranking), Required<br>1 | 1<br>2 | 2<br>3 | 3<br>4 | 4<br>5 | 5

	218	[sub3d_h_3b]<br><br>Show the field ONLY if:<br>[sub3d_h] = '1'	personnel	radio (Matrix - ranking), Required<br>1 1<br>2 2<br>3 3<br>4 4<br>5 5
	219	[sub3d_h_3c]<br><br>Show the field ONLY if:<br>[sub3d_h] = '1'	equipment	radio (Matrix - ranking), Required<br>1 1<br>2 2<br>3 3<br>4 4<br>5 5
	220	[sub3d_h_3d]<br><br>Show the field ONLY if:<br>[sub3d_h] = '1'	standardized protocols	radio (Matrix - ranking), Required<br>1 1<br>2 2<br>3 3<br>4 4<br>5 5
	221	[sub3d_h_3e]<br><br>Show the field ONLY if:<br>[sub3d_h] = '1'	training	radio (Matrix - ranking), Required<br>1 1<br>2 2<br>3 3<br>4 4<br>5 5
	222	[sub3d_h_3_a]<br><br>Show the field ONLY if:<br>[sub3d_h] = '1'	Please describe anything else you might require to help expand your agency's capacity to deploy habitat or landscape management tick control:	text

<table>
<tr><td></td><td>223</td><td>[sub3d_l_1]

Show the field ONLY if:
[sub3d_h] = '1'</td><td>Please select any other potential roadblocks to the development or expansion of your agency's capacity to deploy habitat or landscape management tick control. [select all that apply]</td><td>checkbox, Required<table>
<tr><td>1</td><td>sub3d_l_1___1</td><td>constrained by legislative mandate</td></tr>
<tr><td>2</td><td>sub3d_l_1___2</td><td>concerns for safety of staff</td></tr>
<tr><td>3</td><td>sub3d_l_1___3</td><td>public perceptions of environmental concerns</td></tr>
<tr><td>4</td><td>sub3d_l_1___4</td><td>public perceptions of personal/family health concerns</td></tr>
<tr><td>5</td><td>sub3d_l_1___5</td><td>limited evidence of control efficacy</td></tr>
<tr><td>6</td><td>sub3d_l_1___6</td><td>lack of administrative support</td></tr>
<tr><td>7</td><td>sub3d_l_1___7</td><td>public not likely to use information</td></tr>
<tr><td>8</td><td>sub3d_l_1___8</td><td>high risk areas protected under federal and state regulation</td></tr>
<tr><td>9</td><td>sub3d_l_1___9</td><td>other</td></tr>
</table></td></tr>
<tr><td></td><td>224</td><td>[sub3d_l_1_a]

Show the field ONLY if:
[sub3d_l_1(9)] = '1'</td><td>Please describe any other potential roadblocks to the development or expansion of your agency's capacity to deploy habitat or landscape management tick control:</td><td>text</td></tr>
</table>

	225	[sub3d_h_4]<br><br>Show the field ONLY if:<br>[sub3d_h] = '0'	Why is your agency uninterested in deploying habitat or landscape management tick control?[select all that apply]	checkbox, Required<br>1 | sub3d_h_4___1 | ticks and tickborne disease are not a priority concern in our jurisdiction<br>2 | sub3d_h_4___2 | constrained by legislative mandate<br>3 | sub3d_h_4___3 | concerns for safety of staff<br>4 | sub3d_h_4___4 | public perceptions of environmental concerns<br>5 | sub3d_h_4___5 | public perceptions of personal/family health concerns<br>6 | sub3d_h_4___6 | limited evidence of control efficacy<br>7 | sub3d_h_4___7 | lack of administrative support<br>8 | sub3d_h_4___8 | lack of trained personnel<br>9 | sub3d_h_4___9 | other
	226	[sub3d_h_4_a]<br><br>Show the field ONLY if:<br>[sub3d_h_4(9)] = '1'	Please describe any other reason(s) your agency is uninterested in deploying habitat or landscape management tick control:	text
	227	[subsection_3d_habitat_or_landscape_management_complete]	Section Header: *Form Status*<br><br>Complete?	dropdown<br>0 | Incomplete<br>1 | Unverified<br>2 | Complete

Instrument: **Subsection 3b: Deer targeted intervention** (subsection_3b_deer_targeted_intervention) Enabled as survey

<table>
<tr><td></td><td>228</td><td>[sub3b_i]

Show the field ONLY if:
[sub3_c_methods_used(2)] = '1'</td><td>Section Header: Capacity to deploy deer-targeted tick control

What deer-targeted tick control methods does your agency deploy?[select all that apply]</td><td>checkbox, Required<table>
<tr><td>1</td><td>sub3b_i___1</td><td>deer removal</td></tr>
<tr><td>2</td><td>sub3b_i___2</td><td>deer reproductive control</td></tr>
<tr><td>3</td><td>sub3b_i___3</td><td>4-poster for topical application of acaricide to deer</td></tr>
<tr><td>4</td><td>sub3b_i___4</td><td>deer fencing</td></tr>
<tr><td>5</td><td>sub3b_i___5</td><td>planting plants unpalatable to deer</td></tr>
<tr><td>6</td><td>sub3b_i___6</td><td>other</td></tr>
</table></td></tr>
<tr><td></td><td>229</td><td>[sub3b_i_1]

Show the field ONLY if:
[sub3b_i(6)] = '1'</td><td>Please describe any other deer-targeted tick control method(s) your agency deploys:</td><td>text</td></tr>
<tr><td></td><td>230</td><td>[sub3b_a]

Show the field ONLY if:
[sub3_c_methods_used(2)] = '1'</td><td>What are the funding sources for your agency's deer-targeted tick control?[select all that apply]</td><td>checkbox, Required<table>
<tr><td>1</td><td>sub3b_a___1</td><td>local property taxes</td></tr>
<tr><td>2</td><td>sub3b_a___2</td><td>state taxes</td></tr>
<tr><td>3</td><td>sub3b_a___3</td><td>county taxes</td></tr>
<tr><td>4</td><td>sub3b_a___4</td><td>town or city taxes</td></tr>
<tr><td>5</td><td>sub3b_a___5</td><td>private donations</td></tr>
<tr><td>6</td><td>sub3b_a___6</td><td>surcharge on services or goods</td></tr>
<tr><td>7</td><td>sub3b_a___7</td><td>federal funds</td></tr>
<tr><td>8</td><td>sub3b_a___8</td><td>other</td></tr>
</table></td></tr>
<tr><td></td><td>231</td><td>[sub3b_a_1]

Show the field ONLY if:
[sub3b_a(8)] = '1'</td><td>Please describe any other funding source(s) your agency uses for deer-targeted tick control:</td><td>text</td></tr>
</table>

	232	[sub3b_b]<br>Show the field ONLY if:<br>[sub3_c_methods_used(2)] = '1'	What triggers your agency to deploy deer-targeted tick control on public property?[select all that apply]	checkbox, Required<br>1 sub3b_b___1 in response to tick surveillance data<br>2 sub3b_b___2 in response to human case data<br>3 sub3b_b___3 ongoing program deployed at predetermined sites<br>4 sub3b_b___4 based on public complaint or request<br>5 sub3b_b___5 other
	233	[sub3b_b_1]<br>Show the field ONLY if:<br>[sub3b_b(5)] = '1'	Please describe any other reason(s) your agency deploys deer-targeted tick control:	text
	234	[sub3b_c]<br>Show the field ONLY if:<br>[sub3_c_methods_used(2)] = '1'	Approximately what size area is covered by your agency's deer-targeted tick control?	radio, Required<br>1 0 - 1 acre (0 - 0.001 sq mi)<br>2 1 - 10 acres (0.001 - 0.016 sq mi)<br>3 10 - 100 acres (0.01 - 0.156 sq mi)<br>4 100 - 1000 acres (0.156 - 1.563 sq mi)<br>6 1,000 - 10,000 acres (1.563 - 15.625 sq mi)<br>7 > 10,000 acres (> 15.625 sq mi)
	235	[sub3b_d_1]<br>Show the field ONLY if:<br>[sub3_c_methods_used(2)] = '1'	What type(s) of public property does your agency target to deploy deer-targeted tick control?[select all that apply]	checkbox, Required<br>1 sub3b_d_1___1 parks<br>2 sub3b_d_1___2 picnic areas<br>3 sub3b_d_1___3 community centers<br>4 sub3b_d_1___4 school grounds<br>5 sub3b_d_1___5 forested areas<br>6 sub3b_d_1___6 campgrounds<br>7 sub3b_d_1___7 summer camps<br>8 sub3b_d_1___8 golf course<br>9 sub3b_d_1___9 other

	236	[sub3b_d_1_a]<br><br>Show the field ONLY if:<br>[sub3b_d_1(9)] = '1'	Please describe any other type(s) of public property your agency targets with deer-targeted tick control:	text
	237	[sub3b_d]<br><br>Show the field ONLY if:<br>[sub3_c_methods_used(2)] = '1'	Does your agency target private property when deploying deer-targeted tick control?	yesno, Required<br>1 Yes<br>0 No
	238	[sub3b_d_3]<br><br>Show the field ONLY if:<br>[sub3b_d] = '1'	Please briefly name or describe the deer-targeted methods your agency deploys on private property:	text
	239	[sub3b_d_2]<br><br>Show the field ONLY if:<br>[sub3b_d] = '1'	What triggers your agency to deploy deer-targeted tick control on private property?[select all that apply]	checkbox, Required<br>1 sub3b_d_2___1 landowner request<br>2 sub3b_d_2___2 according to field surveillance data<br>3 sub3b_d_2___3 according to human case data<br>4 sub3b_d_2___4 other
	240	[sub3b_d_2_1]<br><br>Show the field ONLY if:<br>[sub3b_d_2(4)] = '1'	Please describe any other reason(s) that trigger your agency to deploy deer-targeted tick control on private property:	text

	241	[sub3b_e]<br><br>Show the field ONLY if:<br>[sub3_c_methods_used(2)] = '1'	How does your agency evaluate the efficacy of deer-targeted tick control?[select all that apply]	checkbox, Required<br>1 sub3b_e___1 reduction of tick density based on surveillance (e.g. tick flagging / dragging)<br>2 sub3b_e___2 reduction in ticks submitted by the public<br>3 sub3b_e___3 reduction in public complaints or service requests<br>4 sub3b_e___4 reduction in human tickborne disease cases<br>5 sub3b_e___5 other evaluation metric<br>6 sub3b_e___6 unfamiliar with evaluation methods<br>7 sub3b_e___7 no evaluation
	242	[sub3b_e_1]<br><br>Show the field ONLY if:<br>[sub3b_e(5)] = '1'	Please describe any other way(s) your agency evaluates deer-targeted tick control:	text
	243	[sub3b_g]<br><br>Show the field ONLY if:<br>[sub3_c_methods_used(2)] = '1'	Do you want to expand your agency's capacity to deploy deer-targeted tick control on public property?	yesno, Required<br>1 Yes<br>0 No
	244	[sub3b_g_1_header]<br><br>Show the field ONLY if:<br>[sub3b_g] = '1'	What would most help expand your agency's capacity to deploy deer-targeted tick control on public property?<br><br>Please rank below choices in order of priority, with highest priority as '1' and lowest priority as '5':	descriptive
	245	[sub3b_g_1a]<br><br>Show the field ONLY if:<br>[sub3b_g] = '1'	funding	radio (Matrix - ranking), Required<br>1 1<br>2 2<br>3 3<br>4 4<br>5 5

	246	[sub3b_g_1b]<br><br>Show the field ONLY if:<br>[sub3b_g] = '1'	personnel	radio (Matrix - ranking), Required<br>1 1<br>2 2<br>3 3<br>4 4<br>5 5
	247	[sub3b_g_1c]<br><br>Show the field ONLY if:<br>[sub3b_g] = '1'	equipment	radio (Matrix - ranking), Required<br>1 1<br>2 2<br>3 3<br>4 4<br>5 5
	248	[sub3b_g_1d]<br><br>Show the field ONLY if:<br>[sub3b_g] = '1'	standardized protocols	radio (Matrix - ranking), Required<br>1 1<br>2 2<br>3 3<br>4 4<br>5 5
	249	[sub3b_g_1e]<br><br>Show the field ONLY if:<br>[sub3b_g] = '1'	training	radio (Matrix - ranking), Required<br>1 1<br>2 2<br>3 3<br>4 4<br>5 5
	250	[sub3b_g_1_a]<br><br>Show the field ONLY if:<br>[sub3b_g] = '1'	Please describe anything else you might require to help expand your agency's capacity to deploy deer-targeted tick control on public property:	text

	251	[sub3b_k_1]<br>Show the field ONLY if:<br>[sub3b_g] = '1'	Please select any other potential roadblocks to the development or expansion of your agency's capacity to deploy deer-targeted tick control.[select all that apply]	checkbox, Required<br>1 sub3b_k_1___1 constrained by legislative mandate<br>2 sub3b_k_1___2 concerns for safety of staff<br>3 sub3b_k_1___3 public perceptions of environmental concerns<br>4 sub3b_k_1___4 public perceptions of personal/family health concerns<br>5 sub3b_k_1___5 limited evidence of control efficacy<br>6 sub3b_k_1___6 lack of administrative support<br>7 sub3b_k_1___7 public not likely to use information<br>8 sub3b_k_1___8 high risk areas protected under federal and state regulation<br>9 sub3b_k_1___9 local / state / federal agency concern with effects on wildlife population / health<br>10 sub3b_k_1___10 other
	252	[sub3b_k_1_a]<br>Show the field ONLY if:<br>[sub3b_k_1(10)] = '1'	Please describe any other potential roadblocks to the development or expansion of your agency's capacity to deploy deer-targeted tick control:	text
	253	[sub3b_h]<br>Show the field ONLY if:<br>[sub3_c_methods_used(2)] = '0'	If resources, including funding and training opportunities, were available, would your agency be interested in developing the capacity to deploy deer-targeted tick control?	yesno, Required<br>1 Yes<br>0 No

<table>
<tr><td></td><td>254</td><td>[sub3b_h_1]

Show the field ONLY if:
[sub3b_h] = '1'</td><td>If your agency were to deploy deer-targeted tick control, what type(s) of public property would be most feasible for your agency to target?[select all that apply]</td><td>checkbox, Required<table><tr><td>1</td><td>sub3b_h_1___1</td><td>parks</td></tr><tr><td>2</td><td>sub3b_h_1___2</td><td>picnic areas</td></tr><tr><td>3</td><td>sub3b_h_1___3</td><td>community centers</td></tr><tr><td>4</td><td>sub3b_h_1___4</td><td>school grounds</td></tr><tr><td>5</td><td>sub3b_h_1___5</td><td>forested areas</td></tr><tr><td>6</td><td>sub3b_h_1___6</td><td>campgrounds</td></tr><tr><td>7</td><td>sub3b_h_1___7</td><td>summer camps</td></tr><tr><td>8</td><td>sub3b_h_1___8</td><td>golf course</td></tr><tr><td>9</td><td>sub3b_h_1___9</td><td>other</td></tr></table></td></tr>
<tr><td></td><td>255</td><td>[sub3b_h_1_a]

Show the field ONLY if:
[sub3b_h_1(9)] = '1'</td><td>Please describe any other type(s) of public property your agency might target for deer-targeted tick control:</td><td>text</td></tr>
<tr><td></td><td>256</td><td>[sub3b_h_2]

Show the field ONLY if:
[sub3b_h] = '1'</td><td>Would your agency deploy deer-targeted tick control on private property?</td><td>yesno, Required<table><tr><td>1</td><td>Yes</td></tr><tr><td>0</td><td>No</td></tr></table></td></tr>
<tr><td></td><td>257</td><td>[sub3b_h_3_header]

Show the field ONLY if:
[sub3b_h] = '1'</td><td>What would be most helpful to expand your agency's capacity to deploy deer-targeted tick control?

Please rank below choices in order of priority, with highest priority as '1' and lowest priority as '5':</td><td>descriptive</td></tr>
<tr><td></td><td>258</td><td>[sub3b_h_3a]

Show the field ONLY if:
[sub3b_h] = '1'</td><td>funding</td><td>radio (Matrix - ranking), Required<table><tr><td>1</td><td>1</td></tr><tr><td>2</td><td>2</td></tr><tr><td>3</td><td>3</td></tr><tr><td>4</td><td>4</td></tr><tr><td>5</td><td>5</td></tr></table></td></tr>
</table>

	259	[sub3b_h_3b]<br>Show the field ONLY if:<br>[sub3b_h] = '1'	personnel	radio (Matrix - ranking), Required<br>1 1<br>2 2<br>3 3<br>4 4<br>5 5
	260	[sub3b_h_3c]<br>Show the field ONLY if:<br>[sub3b_h] = '1'	equipment	radio (Matrix - ranking), Required<br>1 1<br>2 2<br>3 3<br>4 4<br>5 5
	261	[sub3b_h_3d]<br>Show the field ONLY if:<br>[sub3b_h] = '1'	standardized protocols	radio (Matrix - ranking), Required<br>1 1<br>2 2<br>3 3<br>4 4<br>5 5
	262	[sub3b_h_3e]<br>Show the field ONLY if:<br>[sub3b_h] = '1'	training	radio (Matrix - ranking), Required<br>1 1<br>2 2<br>3 3<br>4 4<br>5 5
	263	[sub3b_h_3_a]<br>Show the field ONLY if:<br>[sub3b_h] = '1'	Please describe anything else you might require to help expand your agency's capacity to deploy deer-targeted tick control:	text

	264	[sub3b_l_1]<br><br>Show the field ONLY if:<br>[sub3b_h] = '1'	Please select any other potential roadblocks to the development or expansion of your agency's capacity to deploy deer-targeted tick control.[select all that apply]	checkbox, Required<br>1 | sub3b_l_1___1 | constrained by legislative mandate<br>2 | sub3b_l_1___2 | concerns for safety of staff<br>3 | sub3b_l_1___3 | public perceptions of environmental concerns<br>4 | sub3b_l_1___4 | public perceptions of personal/family health concerns<br>5 | sub3b_l_1___5 | limited evidence of control efficacy<br>6 | sub3b_l_1___6 | lack of administrative support<br>7 | sub3b_l_1___7 | public not likely to use information<br>8 | sub3b_l_1___8 | high risk areas protected under federal and state regulation<br>9 | sub3b_l_1___9 | local / state / federal agency concern with effects on wildlife population / health<br>10 | sub3b_l_1___10 | other
	265	[sub3b_l_1_a]<br><br>Show the field ONLY if:<br>[sub3b_l_1(10)] = '1'	Please describe any other potential roadblocks to the development or expansion of your agency's capacity to deploy deer-targeted tick control:	text

	266	[sub3b_h_4]<br>Show the field ONLY if:<br>[sub3b_h] = '0'	Why is your agency uninterested in deploying deer-targeted tick control?[select all that apply]	checkbox, Required<br>1 sub3b_h_4___1 ticks and tickborne disease are not a priority concern in our jurisdiction<br>2 sub3b_h_4___2 constrained by legislative mandate<br>3 sub3b_h_4___3 concerns for safety of staff<br>4 sub3b_h_4___4 local / state / federal agency concern with effects on wildlife population / health<br>5 sub3b_h_4___5 public perceptions of environmental concerns<br>6 sub3b_h_4___6 public perceptions of personal/family health concerns<br>7 sub3b_h_4___7 limited evidence of control efficacy<br>8 sub3b_h_4___8 lack of administrative support<br>9 sub3b_h_4___9 lack of trained personnel<br>10 sub3b_h_4___10 other
	267	[sub3b_h_4_a_2]<br>Show the field ONLY if:<br>[sub3b_h_4(10)] = '1'	Please describe any other reason(s) your agency is uninterested in deploying deer-targeted tick control:	text
	268	[subsection_3b_deer_targeted_intervention_complete]	Section Header: *Form Status*<br>Complete?	dropdown<br>0 Incomplete<br>1 Unverified<br>2 Complete

Instrument: **Subsection 4: Tick control guidance for residential property owners** (subsection_4_tick_control_guidance_for_residential)
Enabled as survey

	269	[sub4_a]	Section Header: *Capacity to provide tick control guidance to owners of private residential properties*<br>Does your agency provide guidance to deploy tick control on private, residential properties through educational materials or site visits in your jurisdiction?	radio, Required<br>1 yes<br>2 no
	270	[sub4_aa]	Is there another agency or group that provides guidance to deploy tick control on private, residential properties through educational materials or site visits in your jurisdiction?	yesno, Required<br>1 Yes<br>0 No
	271	[sub4_aa1]<br>Show the field ONLY if:<br>[sub4_aa] = '1'	What other groups or agencies provide guidance to deploy tick control on private, residential properties through educational materials or site visits in your jurisdiction? [select all that apply]	checkbox, Required<br>1 sub4_aa1___1 state or local department of health<br>2 sub4_aa1___2 state department of the environment / natural resources / agriculture<br>3 sub4_aa1___3 local parks and recreation agencies<br>4 sub4_aa1___4 university extension services<br>5 sub4_aa1___5 local non-profit<br>6 sub4_aa1___6 other
	272	[sub4_aa1_a]<br>Show the field ONLY if:<br>[sub4_aa1(6)] = '1'	What other groups or agencies provide guidance to deploy tick control on private, residential properties through educational materials or site visits in your jurisdiction?	text
	273	[sub4_a_1]<br>Show the field ONLY if:<br>[sub4_a] = '1'	What are the funding sources for your guidance to deploy tick control on private, residential properties? [select all that apply]	checkbox, Required<br>1 sub4_a_1___1 local property taxes<br>2 sub4_a_1___2 state taxes<br>3 sub4_a_1___3 county taxes<br>4 sub4_a_1___4 town or city taxes<br>5 sub4_a_1___5 private donations<br>6 sub4_a_1___6 surcharge on services or goods<br>7 sub4_a_1___7 federal funds<br>8 sub4_a_1___8 other

<table>
<tr><td></td><td>274</td><td>[sub4_a_1_a]

Show the field ONLY if:
[sub4_a_1(8)] = '1'</td><td>Please describe any other type(s) of funding sources for tick control guidance on private, residential properties:</td><td>text</td></tr>
<tr><td></td><td>275</td><td>[sub4_a_1_b]

Show the field ONLY if:
[sub4_a] = '1'</td><td>For what type(s) of tick control methods does your agency provide guidance to residential property owners?[select all that apply]</td><td>checkbox, Required<table><tr><td>1</td><td>sub4_a_1_b___1</td><td>application of acaricides to vegetation</td></tr><tr><td>2</td><td>sub4_a_1_b___2</td><td>deer-targeted intervention (e.g. deer fencing and botanical deterrents)</td></tr><tr><td>3</td><td>sub4_a_1_b___3</td><td>rodent-targeted intervention</td></tr><tr><td>4</td><td>sub4_a_1_b___4</td><td>habitat or landscape management</td></tr><tr><td>5</td><td>sub4_a_1_b___5</td><td>other</td></tr></table></td></tr>
<tr><td></td><td>276</td><td>[sub4_a_1_c]

Show the field ONLY if:
[sub4_a_1_b(5)] = '1'</td><td>Please describe any other type(s) of tick control guidance your agency provides to residential property owners:</td><td>text</td></tr>
<tr><td></td><td>277</td><td>[sub4_b]

Show the field ONLY if:
[sub4_a] = '1'</td><td>What methods does your agency currently use to communicate directly with the public?[select all that apply]</td><td>checkbox, Required<table><tr><td>1</td><td>sub4_b___1</td><td>websites</td></tr><tr><td>2</td><td>sub4_b___2</td><td>social media</td></tr><tr><td>3</td><td>sub4_b___3</td><td>print materials</td></tr><tr><td>4</td><td>sub4_b___4</td><td>public events</td></tr><tr><td>5</td><td>sub4_b___5</td><td>other</td></tr></table></td></tr>
<tr><td></td><td>278</td><td>[sub4_b_1]

Show the field ONLY if:
[sub4_b(5)] = '1'</td><td>Please describe any other method(s) your agency currently uses to communicate directly with the public:</td><td>text</td></tr>
<tr><td></td><td>279</td><td>[sub4_a_1_d]

Show the field ONLY if:
[sub4_a] = '1'</td><td>Do you want to expand your agency's capacity to provide tick control guidance to residential property owners?</td><td>radio, Required<table><tr><td>1</td><td>yes</td></tr><tr><td>2</td><td>no</td></tr></table></td></tr>
</table>

	280	[sub4_a_1_e_header]<br><br>Show the field ONLY if:<br>[sub4_a_1_d] = '1'	What would be most helpful to expand your agency's capacity to provide tick control guidance to residential property owners?<br><br>Please rank choices below in order or priority, with highest priority as '1' and lowest priority as '5':	descriptive
	281	[sub4_a_1_e1]<br><br>Show the field ONLY if:<br>[sub4_a_1_d] = '1'	funding	radio (Matrix - ranking), Required<br>1 1<br>2 2<br>3 3<br>4 4<br>5 5
	282	[sub4_a_1_e2]<br><br>Show the field ONLY if:<br>[sub4_a_1_d] = '1'	personnel	radio (Matrix - ranking), Required<br>1 1<br>2 2<br>3 3<br>4 4<br>5 5
	283	[sub4_a_1_e3]<br><br>Show the field ONLY if:<br>[sub4_a_1_d] = '1'	equipment	radio (Matrix - ranking), Required<br>1 1<br>2 2<br>3 3<br>4 4<br>5 5

	284	[sub4_a_1_e4]<br>Show the field ONLY if:<br>[sub4_a_1_d] = '1'	standardized protocols	radio (Matrix - ranking), Required<br>1 1<br>2 2<br>3 3<br>4 4<br>5 5
	285	[sub4_a_1_e5]<br>Show the field ONLY if:<br>[sub4_a_1_d] = '1'	training	radio (Matrix - ranking), Required<br>1 1<br>2 2<br>3 3<br>4 4<br>5 5
	286	[sub4_a_1_f]<br>Show the field ONLY if:<br>[sub4_a_1_d] = '1'	Please describe anything else you might require to help expand your agency's capacity to provide tick control guidance to residential property owners:	text

	287	[sub4_a_1_g]<br>Show the field ONLY if:<br>[sub4_a_1_d] = '1'	Please select any other potential roadblocks to the development or expansion of your agency's capacity to provide tick control guidance to residential property owners?[select all that apply]	checkbox, Required<br>1 | sub4_a_1_g___1 | constrained by legislative mandate<br>2 | sub4_a_1_g___2 | concerns for safety of staff<br>3 | sub4_a_1_g___3 | public perceptions of environmental concerns<br>4 | sub4_a_1_g___4 | public perceptions of personal/family health concerns<br>5 | sub4_a_1_g___5 | limited evidence of control efficacy<br>6 | sub4_a_1_g___6 | lack of administrative support<br>7 | sub4_a_1_g___7 | public not likely to use information<br>8 | sub4_a_1_g___8 | inability to effectively disseminate information<br>9 | sub4_a_1_g___9 | other
	288	[sub4_a_2_g_1]<br>Show the field ONLY if:<br>[sub4_a_1_g(9)] = '1'	Please describe any other potential roadblocks to the development or expansion of your agency's capacity to provide tick control guidance to residential property owners:	text
	289	[sub4_a_2]<br>Show the field ONLY if:<br>[sub4_a] = '2'	If resources, including funding and training opportunities, were available, would your agency be interested in providing tick control guidance to residential property owners?	yesno, Required<br>1 | Yes<br>0 | No
	290	[sub4_a_2_a_header]<br>Show the field ONLY if:<br>[sub4_a_2] = '1'	What would be most helpful to expand your agency's capacity to provide tick control guidance to residential property owners?<br><br>Please rank choices below in order or priority, with highest priority as '1' and lowest priority as '5':	descriptive

	291	[sub4_a_2_a1]<br><br>Show the field ONLY if:<br>[sub4_a_2] = '1'	funding	radio (Matrix - ranking), Required<br><table><tr><td>1</td><td>1</td></tr><tr><td>2</td><td>2</td></tr><tr><td>3</td><td>3</td></tr><tr><td>4</td><td>4</td></tr><tr><td>5</td><td>5</td></tr></table>
	292	[sub4_a_2_a2]<br><br>Show the field ONLY if:<br>[sub4_a_2] = '1'	personnel	radio (Matrix - ranking), Required<br><table><tr><td>1</td><td>1</td></tr><tr><td>2</td><td>2</td></tr><tr><td>3</td><td>3</td></tr><tr><td>4</td><td>4</td></tr><tr><td>5</td><td>5</td></tr></table>
	293	[sub4_a_2_a3]<br><br>Show the field ONLY if:<br>[sub4_a_2] = '1'	equipment	radio (Matrix - ranking), Required<br><table><tr><td>1</td><td>1</td></tr><tr><td>2</td><td>2</td></tr><tr><td>3</td><td>3</td></tr><tr><td>4</td><td>4</td></tr><tr><td>5</td><td>5</td></tr></table>
	294	[sub4_a_2_a4]<br><br>Show the field ONLY if:<br>[sub4_a_2] = '1'	standardized protocols	radio (Matrix - ranking), Required<br><table><tr><td>1</td><td>1</td></tr><tr><td>2</td><td>2</td></tr><tr><td>3</td><td>3</td></tr><tr><td>4</td><td>4</td></tr><tr><td>5</td><td>5</td></tr></table>

<table>
<tr><td></td><td>295</td><td>[sub4_a_2_a5]
Show the field ONLY if:
[sub4_a_2] = '1'</td><td>training</td><td>radio (Matrix - ranking), Required<table><tr><td>1</td><td>1</td></tr><tr><td>2</td><td>2</td></tr><tr><td>3</td><td>3</td></tr><tr><td>4</td><td>4</td></tr><tr><td>5</td><td>5</td></tr></table></td></tr>
<tr><td></td><td>296</td><td>[sub4_a_2_b]
Show the field ONLY if:
[sub4_a_2] = '1'</td><td>Please describe anything else you might require to help expand your agency's capacity to provide tick control guidance to residential property owners:</td><td>text</td></tr>
<tr><td></td><td>297</td><td>[sub4_a_2_e]
Show the field ONLY if:
[sub4_a_2] = '1'</td><td>Please select any other potential roadblocks to the development or expansion of your agency's capacity to provide tick control guidance to residential property owners?[select all that apply]</td><td>checkbox, Required<table><tr><td>1</td><td>sub4_a_2_e___1</td><td>constrained by legislative mandate</td></tr><tr><td>2</td><td>sub4_a_2_e___2</td><td>concerns for safety of staff</td></tr><tr><td>3</td><td>sub4_a_2_e___3</td><td>public perceptions of environmental concerns</td></tr><tr><td>4</td><td>sub4_a_2_e___4</td><td>public perceptions of personal/family health concerns</td></tr><tr><td>5</td><td>sub4_a_2_e___5</td><td>limited evidence of control efficacy</td></tr><tr><td>6</td><td>sub4_a_2_e___6</td><td>lack of administrative support</td></tr><tr><td>7</td><td>sub4_a_2_e___7</td><td>public not likely to use information</td></tr><tr><td>8</td><td>sub4_a_2_e___8</td><td>inability to effectively disseminate information</td></tr><tr><td>9</td><td>sub4_a_2_e___9</td><td>other</td></tr></table></td></tr>
<tr><td></td><td>298</td><td>[sub4_a_2_e_1]
Show the field ONLY if:
[sub4_a_2_e(9)] = '1'</td><td>Please describe any other potential roadblocks to the development or expansion of your agency's capacity to provide tick control guidance to residential property owners:</td><td>text</td></tr>
</table>

	299	[sub4_a_2_c]<br>Show the field ONLY if:<br>[sub4_a_2] = '0'	Why is your agency uninterested in providing tick control guidance to residential property owners?[select all that apply]	checkbox, Required<br>1 | sub4_a_2_c___1 | ticks and tickborne disease are not a priority concern in our jurisdiction<br>2 | sub4_a_2_c___2 | constrained by legislative mandate<br>3 | sub4_a_2_c___3 | concern for expanded public use of acaricides<br>4 | sub4_a_2_c___4 | public perceptions of environmental concerns<br>5 | sub4_a_2_c___5 | public perceptions of personal/family health concerns<br>6 | sub4_a_2_c___6 | limited evidence of control efficacy<br>7 | sub4_a_2_c___7 | lack of administrative support<br>8 | sub4_a_2_c___8 | lack of trained personnel<br>9 | sub4_a_2_c___9 | inability to effectively disseminate information<br>10 | sub4_a_2_c___10 | information already available elsewhere or offered by another organization<br>11 | sub4_a_2_c___11 | other
	300	[sub4_a_2_d]<br>Show the field ONLY if:<br>[sub4_a_2_c(11)] = '1'	Please describe any other reason(s) your agency is uninterested in providing tick control guidance to residential property owners:	text
	301	[sub4_c]<br>Show the field ONLY if:<br>[sub4_a_2] = '1'	How would your agency distribute information relevant to tick and tickborne disease exposure risks in the community, personal tick-bite prevention, and tick control guidance for residential property owners? [select all that apply]	checkbox, Required<br>1 | sub4_c___1 | websites<br>2 | sub4_c___2 | social media<br>3 | sub4_c___3 | print materials<br>4 | sub4_c___4 | public events<br>5 | sub4_c___5 | other

	302	[sub4_c_1]<br>Show the field ONLY if:<br>[sub4_c(5)] = '1'	Please describe any other way(s) that your agency may distribute information relevant to tick and tickborne disease exposure risks in the community, personal tick-bite prevention, and tick control guidance for residential property owners:	text
	303	[subsection_4_tick_control_guidance_for_residential_complete]	Section Header: *Form Status*<br>Complete?	dropdown<br>0 Incomplete<br>1 Unverified<br>2 Complete
Instrument: **Subsection 5: Tick control efficacy assessments** (subsection_5_tick_control_efficacy_assessments) Enabled as survey				
	304	[sub5_a]	Section Header: *Capacity to assess the efficacy of tick control on public and/or private property*<br>Does your agency assess the efficacy of tick control deployed by pest control firms or property owners on public or private property?	yesno, Required<br>1 Yes<br>0 No
	305	[sub5_b]	Is there another group or agency that assesses the efficacy of tick control deployed by pest control firms or property owners in your jurisdiction?	yesno, Required<br>1 Yes<br>0 No
	306	[sub5_b_1]<br>Show the field ONLY if:<br>[sub5_b] = '1'	What other groups or agencies assess the efficacy of tick control deployed by tick control firms or property owners in your jurisdiction?	checkbox, Required<br>1 sub5_b_1___1 state or local department of health<br>2 sub5_b_1___2 state department of the environment / natural resources / agriculture<br>3 sub5_b_1___3 local parks and recreation agencies<br>4 sub5_b_1___4 university extension services<br>5 sub5_b_1___5 pest control firms<br>6 sub5_b_1___6 local non-profit<br>7 sub5_b_1___7 other

	307	[sub5_a_1_a]<br>Show the field ONLY if:<br>[sub5_b_1(7)] = '1'	What other groups or agencies assess the efficacy of tick control deployed by pest control firm or property owners in your jurisdiction?	text
	308	[sub5_a_1]<br>Show the field ONLY if:<br>[sub5_a] = '1'	What are the funding sources for tick control efficacy assessments? [select all that apply]	checkbox, Required<br>1 sub5_a_1___1 local property taxes<br>2 sub5_a_1___2 state taxes<br>3 sub5_a_1___3 county taxes<br>4 sub5_a_1___4 town or city taxes<br>5 sub5_a_1___5 private donations<br>6 sub5_a_1___6 surcharge on services or goods<br>7 sub5_a_1___7 federal funds<br>8 sub5_a_1___8 other
	309	[sub5_a_2]<br>Show the field ONLY if:<br>[sub5_a_1(8)] = '1'	Please describe any other funding source(s) for tick control efficacy assessments:	text
	310	[sub5_a_7]<br>Show the field ONLY if:<br>[sub5_a] = '1'	Does your agency assess the efficacy of tick control for private land owners or pest control firms?	checkbox, Required<br>1 sub5_a_7___1 private landowners<br>2 sub5_a_7___2 pest control firms
	311	[sub5_a_3]<br>Show the field ONLY if:<br>[sub5_a] = '1'	At what scale are efficacy assessments conducted? [select all that apply]	checkbox, Required<br>1 sub5_a_3___1 individual residential property<br>2 sub5_a_3___2 individual public property<br>3 sub5_a_3___3 groups of residential properties<br>4 sub5_a_3___4 groups of public properties<br>5 sub5_a_3___5 high-use public spaces<br>6 sub5_a_3___6 other
	312	[sub5_a_3_a]<br>Show the field ONLY if:<br>[sub5_a_3(6)] = '1'	Please describe any other scale(s) at which efficacy assessments are conducted:	text

	313	[sub5_a_4]<br>Show the field ONLY if:<br>[sub5_a] = '1'	How are efficacy assessments organized or requested? [select all that apply]	checkbox, Required<br>1 | sub5_a_4___1 | on-demand by individual residents<br>2 | sub5_a_4___2 | on-demand by community group<br>3 | sub5_a_4___3 | prescribed monitoring as determined by vector management program<br>4 | sub5_a_4___4 | other
	314	[sub5_a_4_a]<br>Show the field ONLY if:<br>[sub5_a_4(4)] = '1'	Please describe any other way(s) that your agency organizes requests for efficacy assessments:	text
	315	[sub5_a_5]<br>Show the field ONLY if:<br>[sub5_a] = '1'	Do you want to expand your agency's capacity to assess tick control efficacy on public property?	radio, Required<br>1 | yes<br>2 | no
	316	[sub5_a_5_a_header]<br>Show the field ONLY if:<br>[sub5_a_5] = '1'	What would be most helpful to expand your agency's capacity to assess tick control efficacy on public property?<br><br>Please rank below choices in order of priority, with highest priority as '1' and lowest priority as '5':	descriptive
	317	[sub5_a_5_a1]<br>Show the field ONLY if:<br>[sub5_a_5] = '1'	funding	radio (Matrix - ranking), Required<br>1 | 1<br>2 | 2<br>3 | 3<br>4 | 4<br>5 | 5
	318	[sub5_a_5_a2]<br>Show the field ONLY if:<br>[sub5_a_5] = '1'	personnel	radio (Matrix - ranking), Required<br>1 | 1<br>2 | 2<br>3 | 3<br>4 | 4<br>5 | 5

	319	[sub5_a_5_a3]<br>Show the field ONLY if:<br>[sub5_a_5] = '1'	equipment	radio (Matrix - ranking), Required<br>1 1<br>2 2<br>3 3<br>4 4<br>5 5
	320	[sub5_a_5_a4]<br>Show the field ONLY if:<br>[sub5_a_5] = '1'	standardized protocols	radio (Matrix - ranking), Required<br>1 1<br>2 2<br>3 3<br>4 4<br>5 5
	321	[sub5_a_5_a5]<br>Show the field ONLY if:<br>[sub5_a_5] = '1'	training	radio (Matrix - ranking), Required<br>1 1<br>2 2<br>3 3<br>4 4<br>5 5
	322	[sub5_a_5_a_1]<br>Show the field ONLY if:<br>[sub5_a_5] = '1'	Please describe anything else you might require to help expand your agency's capacity to assess tick control efforts on public property:	text

<table>
<tr><td></td><td>323</td><td>[sub5_a_5_d]

Show the field ONLY if:
[sub5_a_5] = '1'</td><td>Please select any other potential roadblocks to the development or expansion of your agency's capacity to assess the efficacy of tick control deployed on public property.[select all that apply]</td><td>checkbox, Required<table><tr><td>1</td><td>sub5_a_5_d___1</td><td>constrained by legislative mandate</td></tr><tr><td>2</td><td>sub5_a_5_d___2</td><td>concerns for safety of staff</td></tr><tr><td>3</td><td>sub5_a_5_d___3</td><td>lack of public support</td></tr><tr><td>4</td><td>sub5_a_5_d___4</td><td>lack of administrative support</td></tr><tr><td>5</td><td>sub5_a_5_d___5</td><td>public not likely to use information</td></tr><tr><td>6</td><td>sub5_a_5_d___6</td><td>inability to effectively disseminate information</td></tr><tr><td>7</td><td>sub5_a_5_d___7</td><td>other</td></tr></table></td></tr>
<tr><td></td><td>324</td><td>[sub5_a_5_d_1]

Show the field ONLY if:
[sub5_a_5_d(7)] = '1'</td><td>Please describe any other potential roadblocks to the development or expansion of your agency's capacity to assess the efficacy of tick control deployed on public property:</td><td>text</td></tr>
<tr><td></td><td>325</td><td>[sub5_a_5_b]

Show the field ONLY if:
[sub5_a] = '1'</td><td>Do you want to expand your agency's capacity to assess tick control efficacy on private, residential property?</td><td>radio, Required<table><tr><td>1</td><td>yes</td></tr><tr><td>2</td><td>no</td></tr></table></td></tr>
<tr><td></td><td>326</td><td>[sub5_a_5_c_header]

Show the field ONLY if:
[sub5_a_5_b] = '1'</td><td>What would be most helpful to expand your agency's capacity to assess tick control efforts on private, residential property?

Please rank choices below in order of priority, with highest priority as '1' and lowest priority as '5':</td><td>descriptive</td></tr>
<tr><td></td><td>327</td><td>[sub5_a_5_c1]

Show the field ONLY if:
[sub5_a_5_b] = '1'</td><td>funding</td><td>radio (Matrix), Required<table><tr><td>1</td><td>1</td></tr><tr><td>2</td><td>2</td></tr><tr><td>3</td><td>3</td></tr><tr><td>4</td><td>4</td></tr><tr><td>5</td><td>5</td></tr></table></td></tr>
</table>

	328	[sub5_a_5_c2]<br><br>Show the field ONLY if:<br>[sub5_a_5_b] = '1'	personnel	radio (Matrix), Required<br>1 1<br>2 2<br>3 3<br>4 4<br>5 5
	329	[sub5_a_5_c3]<br><br>Show the field ONLY if:<br>[sub5_a_5_b] = '1'	equipment	radio (Matrix), Required<br>1 1<br>2 2<br>3 3<br>4 4<br>5 5
	330	[sub5_a_5_c4]<br><br>Show the field ONLY if:<br>[sub5_a_5_b] = '1'	standardized protocols	radio (Matrix), Required<br>1 1<br>2 2<br>3 3<br>4 4<br>5 5
	331	[sub5_a_5_c5]<br><br>Show the field ONLY if:<br>[sub5_a_5_b] = '1'	training	radio (Matrix), Required<br>1 1<br>2 2<br>3 3<br>4 4<br>5 5
	332	[sub5_a_5_c_1]<br><br>Show the field ONLY if:<br>[sub5_a_5_b] = '1'	Please describe anything else you might require to help expand your agency's capacity to assess tick control efforts on private, residential property:	text

	333	[sub5_a_5_e]<br><br>Show the field ONLY if:<br>[sub5_a_5_b] = '1'	Please select any other potential roadblocks to the development or expansion of your agency's capacity to assess the efficacy of tick control deployed on private, residential property.[select all that apply]	checkbox, Required<br>1 | sub5_a_5_e___1 | constrained by legislative mandate<br>2 | sub5_a_5_e___2 | lack of public support<br>3 | sub5_a_5_e___3 | lack of administrative support<br>4 | sub5_a_5_e___4 | public not likely to use information<br>5 | sub5_a_5_e___5 | inability to effectively disseminate this service<br>6 | sub5_a_5_e___6 | other
	334	[sub5_a_5_e_1]<br><br>Show the field ONLY if:<br>[sub5_a_5_e(6)] = '1'	Please describe any other potential roadblocks to the development or expansion of your agency's capacity to assess the efficacy of tick control deployed on private, residential property:	text
	335	[sub5_a_6]<br><br>Show the field ONLY if:<br>[sub5_a] = '0'	If resources, including funding and training opportunities, were available, would your agency be interested in assessing tick control efficacy?	radio, Required<br>1 | yes<br>2 | no
	336	[sub5_a_6_a]<br><br>Show the field ONLY if:<br>[sub5_a_6] = '1'	At what scale is it realistic to conduct tick control efficacy assessments? [select all that apply]	checkbox, Required<br>1 | sub5_a_6_a___1 | individual residential property<br>2 | sub5_a_6_a___2 | individual public property<br>3 | sub5_a_6_a___3 | groups of residential properties<br>4 | sub5_a_6_a___4 | groups of public properties<br>5 | sub5_a_6_a___5 | high-use public spaces<br>6 | sub5_a_6_a___6 | other
	337	[sub5_a_6_b]<br><br>Show the field ONLY if:<br>[sub5_a_6_a(6)] = '1'	Please describe any other scale(s)s at which it is realistic to conduct tick control efficacy assessments:	text

	338	[sub5_a_6_c]<br><br>Show the field ONLY if:<br>[sub5_a_6] = '1'	How would your agency organize requests for tick control efficacy assessments?[select all that apply]	checkbox, Required<br>1 | sub5_a_6_c___1 | on-demand by individual residents<br>2 | sub5_a_6_c___2 | community-organized<br>3 | sub5_a_6_c___3 | prescribed monitoring as determined by vector management program<br>4 | sub5_a_6_c___4 | other
	339	[sub5_a_6_c_1]<br><br>Show the field ONLY if:<br>[sub5_a_6_c(4)] = '1'	Please describe any other way(s) that your agency may organize requests for efficacy assessments:	text
	340	[sub5_a_6_e_header]<br><br>Show the field ONLY if:<br>[sub5_a_6_a(2)] = '1' or [sub5_a_6_a(4)] = '1' or [sub5_a_6_a(5)] = '1'	What would be most helpful to expand your agency's capacity to assess tick control efforts on public property?<br><br>Please rank choices below in order of priority, with highest priority as '1' and lowest priority as '5':	descriptive
	341	[sub5_a_6_e1]<br><br>Show the field ONLY if:<br>[sub5_a_6_a(2)] = '1' or [sub5_a_6_a(4)] = '1' or [sub5_a_6_a(5)] = '1'	funding	radio (Matrix - ranking), Required<br>1 | 1<br>2 | 2<br>3 | 3<br>4 | 4<br>5 | 5
	342	[sub5_a_6_e2]<br><br>Show the field ONLY if:<br>[sub5_a_6_a(2)] = '1' or [sub5_a_6_a(4)] = '1' or [sub5_a_6_a(5)] = '1'	personnel	radio (Matrix - ranking), Required<br>1 | 1<br>2 | 2<br>3 | 3<br>4 | 4<br>5 | 5

	343	[sub5_a_6_e3]<br>Show the field ONLY if: [sub5_a_6_a(2)] = '1' or [sub5_a_6_a(4)] = '1' or [sub5_a_6_a(5)] = '1'	equipment	radio (Matrix - ranking), Required<br>1 1<br>2 2<br>3 3<br>4 4<br>5 5
	344	[sub5_a_6_e4]<br>Show the field ONLY if: [sub5_a_6_a(2)] = '1' or [sub5_a_6_a(4)] = '1' or [sub5_a_6_a(5)] = '1'	standardized protocols	radio (Matrix - ranking), Required<br>1 1<br>2 2<br>3 3<br>4 4<br>5 5
	345	[sub5_a_6_e5]<br>Show the field ONLY if: [sub5_a_6_a(2)] = '1' or [sub5_a_6_a(4)] = '1' or [sub5_a_6_a(5)] = '1'	training	radio (Matrix - ranking), Required<br>1 1<br>2 2<br>3 3<br>4 4<br>5 5
	346	[sub5_a_6_e_1]<br>Show the field ONLY if: [sub5_a_6_a(2)] = '1' or [sub5_a_6_a(4)] = '1' or [sub5_a_6_a(5)] = '1'	Please describe anything else you might require to help expand your agency's capacity to assess tick control efforts on public property:	text

	347	[sub5_a_6_h]<br><br>Show the field ONLY if:<br>[sub5_a_6_a(2)] = '1' or [sub5_a_6_a(4)] = '1' or [sub5_a_6_a(5)] = '1'	Please select any other potential roadblocks to the development or expansion of your agency's capacity to assess the efficacy of tick control deployed on public property.[select all that apply]	checkbox, Required<br>1 | sub5_a_6_h___1 | constrained by legislative mandate<br>2 | sub5_a_6_h___2 | lack of public support<br>3 | sub5_a_6_h___3 | lack of administrative support<br>4 | sub5_a_6_h___4 | public not likely to use information<br>5 | sub5_a_6_h___5 | inability to effectively disseminate this service<br>6 | sub5_a_6_h___6 | other
	348	[sub5_a_6_h_1]<br><br>Show the field ONLY if:<br>[sub5_a_6_h(6)] = '1'	Please describe any other potential roadblocks to the development or expansion of your agency's capacity to assess the efficacy of tick control deployed on public property:	text
	349	[sub5_a_6_f_header]<br><br>Show the field ONLY if:<br>[sub5_a_6_a(1)] = '1' or [sub5_a_6_a(3)] = '1'	What would be most helpful to expand your agency's capacity to assess tick control efforts on private, residential property?<br><br>Please rank choices below in order of priority, with highest priority as '1' and lowest priority as '5':	descriptive
	350	[sub5_a_6_f1]<br><br>Show the field ONLY if:<br>[sub5_a_6_a(1)] = '1' or [sub5_a_6_a(3)] = '1'	funding	radio (Matrix - ranking), Required<br>1 | 1<br>2 | 2<br>3 | 3<br>4 | 4<br>5 | 5

	351	[sub5_a_6_f2]<br>Show the field ONLY if:<br>[sub5_a_6_a(1)] = '1' or [sub5_a_6_a(3)] = '1'	personnel	radio (Matrix - ranking), Required<br>1 1<br>2 2<br>3 3<br>4 4<br>5 5
	352	[sub5_a_6_f3]<br>Show the field ONLY if:<br>[sub5_a_6_a(1)] = '1' or [sub5_a_6_a(3)] = '1'	equipment	radio (Matrix - ranking), Required<br>1 1<br>2 2<br>3 3<br>4 4<br>5 5
	353	[sub5_a_6_f4]<br>Show the field ONLY if:<br>[sub5_a_6_a(1)] = '1' or [sub5_a_6_a(3)] = '1'	standardized protocols	radio (Matrix - ranking), Required<br>1 1<br>2 2<br>3 3<br>4 4<br>5 5
	354	[sub5_a_6_f5]<br>Show the field ONLY if:<br>[sub5_a_6_a(1)] = '1' or [sub5_a_6_a(3)] = '1'	training	radio (Matrix - ranking), Required<br>1 1<br>2 2<br>3 3<br>4 4<br>5 5
	355	[sub5_a_6_f_1]<br>Show the field ONLY if:<br>[sub5_a_6_a(1)] = '1' or [sub5_a_6_a(3)] = '1'	Please describe anything else you might require to help expand your agency's capacity to assess tick control efforts on private, residential property:	text

<table>
<tr><td></td><td>356</td><td>[sub5_a_6_i]

Show the field ONLY if:
[sub5_a_6_a(1)] = '1' or [sub5_a_6_a(3)] = '1'</td><td>Please select any other potential roadblocks to the development or expansion of your agency's capacity to assess the efficacy of tick control deployed on private, residential property.[select all that apply]</td><td>checkbox, Required<table>
<tr><td>1</td><td>sub5_a_6_i___1</td><td>constrained by legislative mandate</td></tr>
<tr><td>2</td><td>sub5_a_6_i___2</td><td>lack of public support</td></tr>
<tr><td>3</td><td>sub5_a_6_i___3</td><td>lack of administrative support</td></tr>
<tr><td>4</td><td>sub5_a_6_i___4</td><td>public not likely to use information</td></tr>
<tr><td>5</td><td>sub5_a_6_i___5</td><td>inability to effectively disseminate information</td></tr>
<tr><td>6</td><td>sub5_a_6_i___6</td><td>other</td></tr>
</table></td></tr>
<tr><td></td><td>357</td><td>[sub5_a_6_i_1]

Show the field ONLY if:
[sub5_a_6_i(6)] = '1'</td><td>Please describe any other potential roadblocks to the development or expansion of your agency's capacity to assess the efficacy of tick control deployed on private, residential property:</td><td>text</td></tr>
<tr><td></td><td>358</td><td>[sub5_a_6_g]

Show the field ONLY if:
[sub5_a_6] = '2'</td><td>Why is your agency uninterested in assessing the efficacy of tick control deployed by property owners and pest control firms? [select all that apply]</td><td>checkbox, Required<table>
<tr><td>1</td><td>sub5_a_6_g___1</td><td>ticks and tickborne disease are not a priority concern in our jurisdiction</td></tr>
<tr><td>2</td><td>sub5_a_6_g___2</td><td>constrained by legislative mandate</td></tr>
<tr><td>3</td><td>sub5_a_6_g___3</td><td>lack of public support</td></tr>
<tr><td>4</td><td>sub5_a_6_g___4</td><td>lack of administrative support</td></tr>
<tr><td>5</td><td>sub5_a_6_g___5</td><td>lack of trained personnel</td></tr>
<tr><td>6</td><td>sub5_a_6_g___6</td><td>service area too large</td></tr>
<tr><td>7</td><td>sub5_a_6_g___7</td><td>inability to effectively disseminate information</td></tr>
<tr><td>8</td><td>sub5_a_6_g___8</td><td>population in service area too large</td></tr>
<tr><td>9</td><td>sub5_a_6_g___9</td><td>information already available elsewhere or collected by another organization</td></tr>
<tr><td>10</td><td>sub5_a_6_g___10</td><td>other</td></tr>
</table></td></tr>
</table>

	359	[sub5_a_6_g_1]<br><br>Show the field ONLY if:<br>[sub5_a_6_g(10)] = '1'	Please describe any other reason(s) your agency is uninterested in assessing the efficacy of tick control deployed by property owners and pest control firms:	text
	360	[subsection_5_tick_control_efficacy_assessments_complete]	Section Header: *Form Status*<br><br>Complete?	dropdown<br>0 Incomplete<br>1 Unverified<br>2 Complete

Instrument: **Subsection 6: Community education regarding personal tick-bite prevention** (subsection_6_community_education_regarding_persona) Enabled as survey

	361	[sub6_a]	Section Header: *Capacity to educate the public regarding personal tick-bite prevention*<br><br>Does your agency create their own educational materials (e.g. website / print / social media / public event displays) regarding personal tick bite prevention?	yesno, Required<br>1 Yes<br>0 No
	362	[sub6_b]	Does your agency work with a group or link to a group's information for education regarding personal tick bite prevention?	yesno, Required<br>1 Yes<br>0 No
	363	[sub6_b_1]<br><br>Show the field ONLY if:<br>[sub6_b] = '1'	What sources of information does your agency work with or link to for education regarding personal tick bite prevention?[select all that apply]	checkbox, Required<br>1 sub6_b_1___1 Centers for Disease Control and Prevention<br>2 sub6_b_1___2 university extension and outreach services<br>3 sub6_b_1___3 state or local health department<br>4 sub6_b_1___4 professional organizations (AMCA, ASTHO, NACCHO, etc.)<br>5 sub6_b_1___5 Lyme disease support groups<br>6 sub6_b_1___6 other
	364	[sub6_b_2]<br><br>Show the field ONLY if:<br>[sub6_b_1(6)] = '1'	What other sources for educational materials does your agency use?	text

	365	[sub6_a_y1]<br><br>Show the field ONLY if:<br>[sub6_a] = '1'	What are the funding sources for tick- bite prevention education? [select all that apply]	checkbox, Required<br>1 | sub6_a_y1___1 | local property taxes<br>2 | sub6_a_y1___2 | state taxes<br>3 | sub6_a_y1___3 | county taxes<br>4 | sub6_a_y1___4 | town or city taxes<br>5 | sub6_a_y1___5 | private donations<br>6 | sub6_a_y1___6 | surcharge on services or goods<br>7 | sub6_a_y1___7 | federal funds<br>8 | sub6_a_y1___8 | other
	366	[sub6_a_y1_a]<br><br>Show the field ONLY if:<br>[sub6_a_y1(8)] = '1'	Please describe any other funding source(s) for tick-bite prevention education:	text
	367	[sub6_a_y2]<br><br>Show the field ONLY if:<br>[sub6_a] = '1'	What are effective ways your agency has found to distribute tick-bite prevention information? [select all that apply]	checkbox, Required<br>1 | sub6_a_y2___1 | video (youtube / vimeo / tik-tok, etc.)<br>2 | sub6_a_y2___2 | social media (facebook / twitter / instagram, etc.)<br>3 | sub6_a_y2___3 | printed materials<br>4 | sub6_a_y2___4 | public forum<br>5 | sub6_a_y2___5 | radio ads<br>6 | sub6_a_y2___6 | tv ads<br>7 | sub6_a_y2___7 | billboards<br>8 | sub6_a_y2___8 | signage on city owned property<br>9 | sub6_a_y2___9 | signage on public transportation<br>10 | sub6_a_y2___10 | other
	368	[sub6_a_y2_a]<br><br>Show the field ONLY if:<br>[sub6_a_y2(10)] = '1'	Please describe any other effective way(s) your agency has found to distribute tick-bite prevention information:	text

	369	[sub6_a_y3]<br>Show the field ONLY if:<br>[sub6_a] = '1'	Do you want to expand your agency's capacity to educate the public about tick-bite prevention?	yesno, Required<br>1 Yes<br>0 No
	370	[sub6_a_y3_matrix]<br>Show the field ONLY if:<br>[sub6_a_y3] = '1'	What resources would be most helpful to expand your agency's capacity to educate the public about tick-bite prevention?Please rank below choices in order of priority, with highest priority as '1' and lowest priority as '5':	descriptive
	371	[sub6_a_y3_matrix_a]<br>Show the field ONLY if:<br>[sub6_a_y3] = '1'	funding	radio (Matrix - ranking), Required<br>1 1<br>2 2<br>3 3<br>4 4<br>5 5
	372	[sub6_a_y3_matrix_b]<br>Show the field ONLY if:<br>[sub6_a_y3] = '1'	personnel	radio (Matrix - ranking), Required<br>1 1<br>2 2<br>3 3<br>4 4<br>5 5
	373	[sub6_a_y3_matrix_c]<br>Show the field ONLY if:<br>[sub6_a_y3] = '1'	access to physical educational material	radio (Matrix - ranking), Required<br>1 1<br>2 2<br>3 3<br>4 4<br>5 5

	374	[sub6_a_y3_matrix_d]<br>Show the field ONLY if:<br>[sub6_a_y3] = '1'	access to digital educational material	radio (Matrix - ranking), Required<br>1 1<br>2 2<br>3 3<br>4 4<br>5 5
	375	[sub6_a_y3_matrix_e]<br>Show the field ONLY if:<br>[sub6_a_y3] = '1'	training	radio (Matrix - ranking), Required<br>1 1<br>2 2<br>3 3<br>4 4<br>5 5
	376	[sub6_a_y3_matrix_other]<br>Show the field ONLY if:<br>[sub6_a_y3] = '1'	Please describe anything else you might require to help expand your agency's capacity to educate the public about tick-bite prevention:	text
	377	[sub6_a_n2]<br>Show the field ONLY if:<br>[sub6_a_y3] = '1'	Please select any other potential roadblocks to the development or expansion of your agency's capacity to educate the public about personal tick-bite prevention. [select all that apply]	checkbox, Required<br>1 sub6_a_n2___1 constrained by legislative mandate<br>2 sub6_a_n2___2 lack of public support<br>3 sub6_a_n2___3 lack of administrative support<br>4 sub6_a_n2___4 public not likely to use information<br>5 sub6_a_n2___5 inability to effectively disseminate information<br>6 sub6_a_n2___6 other
	378	[sub6_a_n2_a]<br>Show the field ONLY if:<br>[sub6_a_n2(6)] = '1'	Please describe any other potential roadblocks to the development or expansion of your agency's capacity to educate the public about personal tick-bite prevention:	text

	379	[sub6_a_n1]<br>Show the field ONLY if:<br>[sub6_a] = '0'	If resources, including funding and training opportunities were available, would your agency be interested in developing the capacity to educate the public about personal tick-bite prevention?	yesno, Required<br>1 Yes<br>0 No
	380	[sub6_a_n1_y1_matrix]<br>Show the field ONLY if:<br>[sub6_a_n1] = '1'	What resources would be most helpful to expand your agency's capacity to educate the public about tick-bite prevention?<br><br>Please rank below choices in order of priority, with highest priority as '1' and lowest priority as '5':	descriptive
	381	[sub6_a_n1_y1_a]<br>Show the field ONLY if:<br>[sub6_a_n1] = '1'	funding	radio (Matrix - ranking), Required<br>1 1<br>2 2<br>3 3<br>4 4<br>5 5
	382	[sub6_a_n1_y1_b]<br>Show the field ONLY if:<br>[sub6_a_n1] = '1'	personnel	radio (Matrix - ranking), Required<br>1 1<br>2 2<br>3 3<br>4 4<br>5 5
	383	[sub6_a_n1_y1_c]<br>Show the field ONLY if:<br>[sub6_a_n1] = '1'	access to physical educational material	radio (Matrix - ranking), Required<br>1 1<br>2 2<br>3 3<br>4 4<br>5 5

	384	[sub6_a_n1_y1_d]<br>Show the field ONLY if:<br>[sub6_a_n1] = '1'	access to digital educational material	radio (Matrix - ranking), Required<br>1 | 1<br>2 | 2<br>3 | 3<br>4 | 4<br>5 | 5
	385	[sub6_a_n1_y1_e]<br>Show the field ONLY if:<br>[sub6_a_n1] = '1'	training	radio (Matrix - ranking), Required<br>1 | 1<br>2 | 2<br>3 | 3<br>4 | 4<br>5 | 5
	386	[sub6_a_n1_y1_matrix_other]<br>Show the field ONLY if:<br>[sub6_a_n1] = '1'	Please describe anything else you might require to help expand your agency's capacity to educate the public about tick-bite prevention:	text
	387	[sub6_a_n4]<br>Show the field ONLY if:<br>[sub6_a_n1] = '1'	Please select any other potential roadblocks to the development or expansion of your agency's capacity to educate the public about personal tick-bite prevention. [select all that apply]	checkbox, Required<br>1 | sub6_a_n4___1 | constrained by legislative mandate<br>2 | sub6_a_n4___2 | lack of public support<br>3 | sub6_a_n4___3 | lack of administrative support<br>4 | sub6_a_n4___4 | public not likely to use information<br>5 | sub6_a_n4___5 | inability to effectively disseminate information<br>6 | sub6_a_n4___6 | other
	388	[sub6_a_n3_a]<br>Show the field ONLY if:<br>[sub6_a_n4(6)] = '1'	Please describe any other potential roadblocks to the development or expansion of your agency's capacity to educate the public about personal tick-bite prevention:	text

	389	[sub6_a_n1_y2]<br><br>Show the field ONLY if:<br>[sub6_a_n1] = '1'	If your agency were to distribute tick-bite prevention information, that dissemination methods would you be likely to use? [select all that apply]	checkbox, Required<br>1 | sub6_a_n1_y2___1 | video (youtube / vimeo / tik-tok, etc.)<br>2 | sub6_a_n1_y2___2 | social media (facebook / twitter / instagram, etc.)<br>3 | sub6_a_n1_y2___3 | printed materials<br>4 | sub6_a_n1_y2___4 | public forum<br>5 | sub6_a_n1_y2___5 | radio ads<br>6 | sub6_a_n1_y2___6 | tv ads<br>7 | sub6_a_n1_y2___7 | billboards<br>8 | sub6_a_n1_y2___8 | signage on city owned property<br>9 | sub6_a_n1_y2___9 | signage on public transportation<br>10 | sub6_a_n1_y2___10 | other
	390	[sub6_a_n1_y2_other]<br><br>Show the field ONLY if:<br>[sub6_a_n1_y2(10)] = '1'	Please describe any other way(s) your agency may effectively distribute information to the public:	text

	391	[sub6_a_n1_n1]<br><br>Show the field ONLY if:<br>[sub6_a_n1] = '0'	Why is your agency uninterested in educating the public about personal tick-bite prevention? [select all that apply]	checkbox, Required<br>1 | sub6_a_n1_n1___1 | ticks and tickborne disease are not a priority concern in our jurisdiction<br>2 | sub6_a_n1_n1___2 | not in agency mission statement<br>3 | sub6_a_n1_n1___3 | lack of public support<br>4 | sub6_a_n1_n1___4 | lack of administrative support<br>5 | sub6_a_n1_n1___5 | public not likely to use information<br>6 | sub6_a_n1_n1___6 | inability to effectively disseminate information<br>7 | sub6_a_n1_n1___7 | information already available elsewhere or offered by another organization<br>8 | sub6_a_n1_n1___8 | other
	392	[sub6_an1_n1_other]<br><br>Show the field ONLY if:<br>[sub6_a_n1_n1(8)] = '1'	Please describe any other reason(s) your agency is uninterested in educating the public about personal tick-bite prevention:	text
	393	[subsection_6_community_education_regarding_persona_complete]	Section Header: *Form Status*<br><br>Complete?	dropdown<br>0 | Incomplete<br>1 | Unverified<br>2 | Complete

Instrument: **Subsection 7: Private operators contracted by government entities** (subsection_7_private_operators_contracted_by_gover)

Enabled as survey

	394	[sub7_a]	Section Header: *Capacity of private operators contracted by government entities to deploy tick control*<br>What type(s) of public agency/agencies are you contracted under?[select all that apply]	checkbox, Required<br>1 | sub7_a___1 | mosquito or vector control district<br>2 | sub7_a___2 | public health agency<br>3 | sub7_a___3 | environmental health agency<br>4 | sub7_a___4 | public works<br>5 | sub7_a___5 | animal control<br>6 | sub7_a___6 | parks and recreation department<br>7 | sub7_a___7 | federal agency<br>8 | sub7_a___8 | other
	395	[sub7_a_1]<br>Show the field ONLY if:<br>[sub7_a(8)] = '1'	Please describe any other type(s) of public agency/agencies by which your company is contracted for vector control:	text
	396	[sub7_b]	What is the jurisdiction of the agency under which you are contracted?[select all that apply]	checkbox, Required<br>1 | sub7_b___1 | city<br>2 | sub7_b___2 | county<br>3 | sub7_b___3 | state<br>4 | sub7_b___4 | regional or multi-state<br>5 | sub7_b___5 | federal<br>6 | sub7_b___6 | other
	397	[sub7_b_1]<br>Show the field ONLY if:<br>[sub7_b(6)] = '1'	Please describe any other jurisdictional level of the agency by which you are contracted:	text

	398	[sub7_c]	At what scale are your current vector control efforts conducted?[select all that apply]	checkbox, Required<br>1 sub7_c___1 individual public property<br>2 sub7_c___2 groups of public properties<br>3 sub7_c___3 high-use public spaces<br>4 sub7_c___4 private residential properties<br>5 sub7_c___5 private non-residential properties<br>6 sub7_c___6 other
	399	[sub7_c_1]<br>Show the field ONLY if:<br>[sub7_c(6)] = '1'	Please describe any other scale(s) of your vector control activities:	text
	400	[sub7_d]	How often is your organization contracted to deploy tick control?	radio, Required<br>1 multiple times per year<br>2 once a year<br>3 never<br>4 other
	401	[sub7_d_1]<br>Show the field ONLY if:<br>[sub7_d] = '4'	Please describe any other frequency/frequencies at which your organization is contracted to deploy tick control:	text

	402	[sub7_e]	Where does your organization acquire information regarding available tick control methods?[select all that apply]	checkbox, Required<br>1 | sub7_e___1 | scientific literature<br>2 | sub7_e___2 | county health department<br>3 | sub7_e___3 | state health department<br>4 | sub7_e___4 | Centers for Disease Control and Prevention<br>5 | sub7_e___5 | university extension services<br>6 | sub7_e___6 | academic institution<br>7 | sub7_e___7 | meetings<br>8 | sub7_e___8 | private companies<br>9 | sub7_e___9 | professional groups<br>10 | sub7_e___10 | other
	403	[sub7_e_1]<br>Show the field ONLY if:<br>[sub7_e(10)] = '1'	Please describe any other source(s) your organization uses to acquire information regarding tick control methods:	text
	404	[sub7_f]<br>Show the field ONLY if:<br>[sub7_d] = '1' or [sub7_d] = '2' or [sub7_d] = '4'	What tick control methods does your organization currently deploy?[select all that apply]	checkbox, Required<br>1 | sub7_f___1 | application of acaricides to vegetation (synthetic, biological, or natural)<br>2 | sub7_f___2 | deer-targeted intervention<br>3 | sub7_f___3 | rodent-targeted intervention<br>4 | sub7_f___4 | habitat or landscape management<br>5 | sub7_f___5 | other
	405	[sub7_f_1]<br>Show the field ONLY if:<br>[sub7_f(5)] = '1'	Please describe any other tick control method(s) your organization deploys:	text

	406	[subsection_7_private_operators_contracted_by_gover_complete]	Section Header: *Form Status*<br>Complete?	dropdown<br>0 Incomplete<br>1 Unverified<br>2 Complete

Instrument: **Subsection 7a: Acaricide Application** (subsection_7a_acaricide_application) Enabled as survey

	407	[sub7a_e]<br>Show the field ONLY if:<br>[sub7_f(1)] = '1'	Section Header: *Capacity of private operators contracted by government entities to deploy acaricides*<br>What types of acaricide products does your organization deploy for tick control?[select all that apply]	checkbox, Required<br>1 sub7a_e___1 synthetic chemical<br>2 sub7a_e___2 natural botanical product<br>3 sub7a_e___3 entomopathogenic fungus<br>4 sub7a_e___4 other
	408	[sub7a_e_1]<br>Show the field ONLY if:<br>[sub7a_e(4)] = '1'	Please describe any other type(s) of acaricide your organization deploys for tick control:	text
	409	[sub7a_b]<br>Show the field ONLY if:<br>[sub7_f(1)] = '1'	Do your organization's acaricide applications target private or public property, or both?	radio, Required<br>1 private properties<br>2 public properties<br>3 both
	410	[sub7a_a]<br>Show the field ONLY if:<br>[sub7a_b] = '2' or [sub7a_b] = '3'	At approximately how many public properties does your organization generally deploy acaricide applications per year?	radio, Required<br>1 < 10<br>2 10 - 50<br>3 50 - 100<br>4 100 - 500<br>5 > 500

	411	[sub7a_a_2]<br><br>Show the field ONLY if:<br>[sub7a_b] = '1' or [sub7a_b] = '3'	At approximately how many private properties does your organization generally deploy acaricide applications per year?	radio, Required<br>1 | < 10<br>2 | 10 - 50<br>3 | 50 - 100<br>4 | 100 - 500<br>5 | > 500
	412	[sub7a_b_1]<br><br>Show the field ONLY if:<br>[sub7a_b] = '2' or [sub7a_b] = '3'	What type(s) of public property does your organization target for acaricide application(s)?[select all that apply]	checkbox, Required<br>1 | sub7a_b_1___1 | parks<br>2 | sub7a_b_1___2 | picnic areas<br>3 | sub7a_b_1___3 | community centers<br>4 | sub7a_b_1___4 | school grounds<br>5 | sub7a_b_1___5 | forested areas<br>6 | sub7a_b_1___6 | campgrounds<br>7 | sub7a_b_1___7 | summer camps<br>8 | sub7a_b_1___8 | golf course<br>9 | sub7a_b_1___9 | other
	413	[sub7a_b_1_a]<br><br>Show the field ONLY if:<br>[sub7a_b_1(9)] = '1'	Please describe any other type(s) of public property your organization targets for acaricide applications:	text
	414	[sub7a_b_2]<br><br>Show the field ONLY if:<br>[sub7a_b] = '1' or [sub7a_b] = '3'	What triggers your organization to deploy acaricides on private property?[select all that apply]	checkbox, Required<br>1 | sub7a_b_2___1 | request of property owners<br>2 | sub7a_b_2___2 | according to field surveillance data<br>3 | sub7a_b_2___3 | according to human case data<br>4 | sub7a_b_2___4 | other
	415	[sub7a_b_2_a]<br><br>Show the field ONLY if:<br>[sub7a_b_2(4)] = '1'	Please describe any other reason(s) that trigger your organization to deploy acaricides on private property:	notes

<table>
<tr><td></td><td>416</td><td>[sub7a_c]

Show the field ONLY if:
[sub7_f(1)] = '1'</td><td>How does your organization evaluate the efficacy of acaricide applications?[select all that apply]</td><td>checkbox, Required<table><tr><td>1</td><td>sub7a_c___1</td><td>reduction in host-seeking ticks</td></tr><tr><td>2</td><td>sub7a_c___2</td><td>reduction in ticks submitted by the public</td></tr><tr><td>3</td><td>sub7a_c___3</td><td>reduction in public complaints or service requests</td></tr><tr><td>4</td><td>sub7a_c___4</td><td>reduction in human tickborne disease cases</td></tr><tr><td>5</td><td>sub7a_c___5</td><td>other evaluation metric</td></tr><tr><td>6</td><td>sub7a_c___6</td><td>no evaluation</td></tr></table></td></tr>
<tr><td></td><td>417</td><td>[sub7a_c_1]

Show the field ONLY if:
[sub7a_c(5)] = '1'</td><td>Please describe any other way(s) that your organization evaluates the efficacy of acaricide applications:</td><td>text</td></tr>
<tr><td></td><td>418</td><td>[sub7a_d]

Show the field ONLY if:
[sub7_f(1)] = '0'</td><td>If training opportunities were available, and you were asked by your contracting agency, would your organization be interested in learning how to deploy acaricide applications?</td><td>yesno, Required<table><tr><td>1</td><td>Yes</td></tr><tr><td>0</td><td>No</td></tr></table></td></tr>
<tr><td></td><td>419</td><td>[sub7a_d_1]

Show the field ONLY if:
[sub7a_d] = '1'</td><td>If your organization were to deploy acaricide applications, what type(s) of public property would be most feasible for your operation to target?[select all that apply]</td><td>checkbox, Required<table><tr><td>1</td><td>sub7a_d_1___1</td><td>parks</td></tr><tr><td>2</td><td>sub7a_d_1___2</td><td>picnic areas</td></tr><tr><td>3</td><td>sub7a_d_1___3</td><td>community centers</td></tr><tr><td>4</td><td>sub7a_d_1___4</td><td>school grounds</td></tr><tr><td>5</td><td>sub7a_d_1___5</td><td>forested areas</td></tr><tr><td>6</td><td>sub7a_d_1___6</td><td>campgrounds</td></tr><tr><td>7</td><td>sub7a_d_1___7</td><td>summer camps</td></tr><tr><td>8</td><td>sub7a_d_1___8</td><td>golf course</td></tr><tr><td>9</td><td>sub7a_d_1___9</td><td>other</td></tr></table></td></tr>
<tr><td></td><td>420</td><td>[sub7a_d_1_a]

Show the field ONLY if:
[sub7a_d_1(9)] = '1'</td><td>Please describe any other type(s) of public property your organization might target for acaricide application:</td><td>text</td></tr>
</table>

	421	[sub7a_d_2]<br><br>Show the field ONLY if:<br>[sub7a_d] = '1'	If your organization were to deploy acaricide applications, would it treat private properties?	yesno, Required<br>1 Yes<br>0 No
	422	[sub7a_d_3]<br><br>Show the field ONLY if:<br>[sub7a_d] = '0'	Why is your organization uninterested in deploying acaricide applications?[select all that apply]	checkbox, Required<br>1 sub7a_d_3___1 ticks and tickborne disease are not a priority concern in our service area<br>2 sub7a_d_3___2 concerns for safety of staff<br>3 sub7a_d_3___3 environmental concerns<br>4 sub7a_d_3___4 lack of interest from contracting agency<br>5 sub7a_d_3___5 lack of trained personnel<br>6 sub7a_d_3___6 not profitable<br>7 sub7a_d_3___7 other
	423	[sub7a_d_3_a]<br><br>Show the field ONLY if:<br>[sub7a_d_3(7)] = '1'	Please describe any other reason(s) your organization is uninterested in deploying acaricide applications:	text
	424	[subsection_7a_acaricide_application_complete]	Section Header: *Form Status*<br><br>Complete?	dropdown<br>0 Incomplete<br>1 Unverified<br>2 Complete

Instrument: **Subsection 7c: Rodent targeted intervention** (subsection_7c_rodent_targeted_intervention_26cc) Enabled as survey

	425	[sub7c_e]<br><br>Show the field ONLY if:<br>[sub7_f(3)] = '1'	Section Header: *Capacity of private operators contracted by government entities to deploy rodent-targeted tick control*<br><br>What rodent-targeted tick control methods does your organization deploy?[select all that apply]	checkbox, Required<br>1 sub7c_e___1 damminix tick tubes<br>2 sub7c_e___2 thermacell tick tubes<br>3 sub7c_e___3 select TCS bait boxes<br>4 sub7c_e___4 other

	426	[sub7c_e_1]<br><br>Show the field ONLY if:<br>[sub7c_e(4)] = '1'	Please describe any other rodent-targeted tick control method(s) your organization deploys:	text
	427	[sub7c_b]<br><br>Show the field ONLY if:<br>[sub7_f(3)] = '1'	Does your organization target private or public property, or both, when deploying rodent-targeted tick control?	radio, Required<br>1 private properties<br>2 public properties<br>3 both
	428	[sub7c_a]<br><br>Show the field ONLY if:<br>[sub7c_b] = '2' or [sub7c_b] = '3'	At approximately how many public properties does your organization deploy rodent-targeted tick control per year?	radio, Required<br>1 < 10<br>2 10 - 50<br>3 50 - 100<br>4 100 - 500<br>5 > 500
	429	[sub7c_a_2]<br><br>Show the field ONLY if:<br>[sub7c_b] = '1' or [sub7c_b] = '3'	At approximately how many private properties does your organization deploy rodent-targeted tick control per year?	radio, Required<br>1 < 10<br>2 10 - 50<br>3 50 - 100<br>4 100 - 500<br>5 > 500

	430	[sub7c_b_1]<br><br>Show the field ONLY if:<br>[sub7c_b] = '2' or [sub7c_b] = '3'	What type(s) of public property does your organization target when deploying rodent-targeted tick control?[select all that apply]	checkbox, Required<br>1 sub7c_b_1___1 parks<br>2 sub7c_b_1___2 picnic areas<br>3 sub7c_b_1___3 community centers<br>4 sub7c_b_1___4 school grounds<br>5 sub7c_b_1___5 forested areas<br>6 sub7c_b_1___6 campgrounds<br>7 sub7c_b_1___7 summer camps<br>8 sub7c_b_1___8 golf course<br>9 sub7c_b_1___9 other
	431	[sub7c_b_1_a]<br><br>Show the field ONLY if:<br>[sub7c_b_1(9)] = '1'	Please describe any other type(s) of public property your organization targets with rodent-targeted tick control:	text
	432	[sub7c_b_2]<br><br>Show the field ONLY if:<br>[sub7c_b] = '1' or [sub7c_b] = '3'	What triggers your organization to deploy rodent-targeted tick control on private property?[select all that apply]	checkbox, Required<br>1 sub7c_b_2___1 request of property owners<br>2 sub7c_b_2___2 according to field surveillance data<br>3 sub7c_b_2___3 according to human case data<br>4 sub7c_b_2___4 other
	433	[sub7c_b_2_a]<br><br>Show the field ONLY if:<br>[sub7c_b_2(4)] = '1'	Please describe any other reason(s) that trigger your organization to deploy rodent-targeted tick control on private property:	notes

	434	[sub7c_c]<br><br>Show the field ONLY if:<br>[sub7_f(3)] = '1'	How does your organization evaluate the efficacy of rodent-targeted tick control?[select all that apply]	checkbox, Required<br>1 | sub7c_c___1 | reduction in host-seeking ticks<br>2 | sub7c_c___2 | reduction in ticks submitted by the public<br>3 | sub7c_c___3 | reduction in public complaints or service requests<br>4 | sub7c_c___4 | reduction in human tickborne disease cases<br>5 | sub7c_c___5 | other evaluation metric<br>6 | sub7c_c___6 | no evaluation
	435	[sub7c_c_1]<br><br>Show the field ONLY if:<br>[sub7c_c(5)] = '1'	Please describe any other way(s) your organization evaluates the efficacy of rodent-targeted tick control:	text
	436	[sub7c_d]<br><br>Show the field ONLY if:<br>[sub7_f(3)] = '0'	If training opportunities were available, and you were asked by your contracting agency, would your organization be interested in developing the capacity to deploy rodent-targeted tick control?	yesno, Required<br>1 | Yes<br>0 | No
	437	[sub7c_d_1]<br><br>Show the field ONLY if:<br>[sub7c_d] = '1'	If your organization were to deploy rodent-targeted tick control, what type(s) of public property would be most feasible for your operation to target?[select all that apply]	checkbox, Required<br>1 | sub7c_d_1___1 | parks<br>2 | sub7c_d_1___2 | picnic areas<br>3 | sub7c_d_1___3 | community centers<br>4 | sub7c_d_1___4 | school grounds<br>5 | sub7c_d_1___5 | forested areas<br>6 | sub7c_d_1___6 | campgrounds<br>7 | sub7c_d_1___7 | summer camps<br>8 | sub7c_d_1___8 | golf course<br>9 | sub7c_d_1___9 | other
	438	[sub7c_d_1_a]<br><br>Show the field ONLY if:<br>[sub7c_d_1(9)] = '1'	Please describe any other type(s) of public property your organization might target for rodent-targeted tick control:	text

	439	[sub7c_d_2]<br><br>Show the field ONLY if:<br>[sub7c_d] = '1'	If your organization were to deploy rodent-targeted tick control, would it target private properties?	yesno, Required<br>1 Yes<br>0 No
	440	[sub7c_d_3]<br><br>Show the field ONLY if:<br>[sub7c_d] = '0'	Why is your organization uninterested in deploying rodent-targeted tick control?[select all that apply]	checkbox, Required<br>1 sub7c_d_3___1 ticks and tickborne disease are not a priority concern in our service area<br>2 sub7c_d_3___2 concerns for safety of staff<br>3 sub7c_d_3___3 environmental concerns<br>4 sub7c_d_3___4 lack of interest from contracting agency<br>5 sub7c_d_3___5 lack of trained personnel<br>6 sub7c_d_3___6 not profitable<br>7 sub7c_d_3___7 other
	441	[sub7c_d_3_a]<br><br>Show the field ONLY if:<br>[sub7c_d_3(7)] = '1'	Please describe any other reason(s) your organization is uninterested in deploying rodent-targeted tick control:	text
	442	[subsection_7c_rodent_targeted_intervention_26cc_complete]	Section Header: *Form Status*<br>Complete?	dropdown<br>0 Incomplete<br>1 Unverified<br>2 Complete

Instrument: **Subsection 7d: Habitat or landscape management** (subsection_7d_habitat_or_landscape_management_52f5) Enabled as survey

	443	[sub7d_e]<br><br>Show the field ONLY if:<br>[sub7_f(4)] = '1'	Section Header: *Capacity of private operators contracted by government entities to deploy habitat or landscape management tick control*<br>What habitat or landscape modification tick control methods does your organization deploy?[select all that apply]	checkbox, Required<br>1 | sub7d_e___1 | mowing<br>2 | sub7d_e___2 | brush clearing<br>3 | sub7d_e___3 | leaf litter removal<br>4 | sub7d_e___4 | xeric borders along high-risk tick habitat<br>5 | sub7d_e___5 | burning<br>6 | sub7d_e___6 | other
	444	[sub7d_e_1]<br><br>Show the field ONLY if:<br>[sub7d_e(6)] = '1'	What other habitat or landscape modification method(s) does your organization deploy for tick control:	text
	445	[sub7d_b]<br><br>Show the field ONLY if:<br>[sub7_f(4)] = '1'	Does your organization conduct habitat or landscape management tick control on private or public property, or both?	radio, Required<br>1 | private properties<br>2 | public properties<br>3 | both
	446	[sub7d_a]<br><br>Show the field ONLY if:<br>[sub7d_b] = '2' or [sub7d_b] = '3'	At approximately how many public properties does your organization conduct habitat or landscape management tick control per year?	radio, Required<br>1 | < 10<br>2 | 10 - 50<br>3 | 50 - 100<br>4 | 100 - 500<br>5 | > 500
	447	[sub7d_a_2]<br><br>Show the field ONLY if:<br>[sub7d_b] = '1' or [sub7d_b] = '3'	At approximately how many private properties does your organization conduct habitat or landscape management tick control per year?	radio, Required<br>1 | < 10<br>2 | 10 - 50<br>3 | 50 - 100<br>4 | 100 - 500<br>5 | > 500

	448	[sub7d_b_1]<br><br>Show the field ONLY if:<br>[sub7d_b] = '2' or [sub7d_b] = '3'	On what type(s) of public property does your organization conduct habitat or landscape management tick control?[select all that apply]	checkbox, Required<br>1 | sub7d_b_1___1 | parks<br>2 | sub7d_b_1___2 | picnic areas<br>3 | sub7d_b_1___3 | community centers<br>4 | sub7d_b_1___4 | school grounds<br>5 | sub7d_b_1___5 | forested areas<br>6 | sub7d_b_1___6 | campgrounds<br>7 | sub7d_b_1___7 | summer camps<br>8 | sub7d_b_1___8 | golf course<br>9 | sub7d_b_1___9 | other
	449	[sub7d_b_1_a]<br><br>Show the field ONLY if:<br>[sub7d_b_1(9)] = '1'	Please describe any other type(s) of public property where your organization conducts habitat or landscape management tick control:	text
	450	[sub7d_b_2]<br><br>Show the field ONLY if:<br>[sub7d_b] = '1' or [sub7d_b] = '3'	How is the need for your organization to use habitat or landscape management tick control methods on private property determined?[select all that apply]	checkbox, Required<br>1 | sub7d_b_2___1 | request of property owners<br>2 | sub7d_b_2___2 | according to field surveillance data<br>3 | sub7d_b_2___3 | according to human case data<br>4 | sub7d_b_2___4 | other
	451	[sub7d_b_2_a]<br><br>Show the field ONLY if:<br>[sub7d_b_2(4)] = '1'	Please describe any other ways that your organization determines the need to conduct habitat or landscape management tick control methods on private property:	notes

	452	[sub7d_c]<br>Show the field ONLY if:<br>[sub7_f(4)] = '1'	How does your organization evaluate the efficacy of habitat or landscape management tick control?[select all that apply]	checkbox, Required<br>1 sub7d_c___1 reduction in host-seeking ticks<br>2 sub7d_c___2 reduction in ticks submitted by the public<br>3 sub7d_c___3 reduction in public complaints or service requests<br>4 sub7d_c___4 reduction in human tickborne disease cases<br>5 sub7d_c___5 other evaluation metric<br>6 sub7d_c___6 no evaluation
	453	[sub7d_c_1]<br>Show the field ONLY if:<br>[sub7d_c(5)] = '1'	Please describe any other way(s) your organization evaluates the efficacy of habitat or landscape management tick control:	text
	454	[sub7d_d]<br>Show the field ONLY if:<br>[sub7_f(4)] = '0'	If training opportunities were available, and you were asked by your contracting agency, would your organization be interested in developing the capacity to conduct habitat or landscape management tick control?	yesno, Required<br>1 Yes<br>0 No
	455	[sub7d_d_1]<br>Show the field ONLY if:<br>[sub7d_d] = '1'	If your organization were to conduct habitat or landscape management tick control, what type(s) of public property would be most feasible for your operation to target?[select all that apply]	checkbox, Required<br>1 sub7d_d_1___1 parks<br>2 sub7d_d_1___2 picnic areas<br>3 sub7d_d_1___3 community centers<br>4 sub7d_d_1___4 school grounds<br>5 sub7d_d_1___5 forested areas<br>6 sub7d_d_1___6 campgrounds<br>7 sub7d_d_1___7 summer camps<br>8 sub7d_d_1___8 golf course<br>9 sub7d_d_1___9 other
	456	[sub7d_d_1_a]<br>Show the field ONLY if:<br>[sub7d_d_1(9)] = '1'	Please describe any other type(s) of public property your organization might target for habitat or landscape management tick control:	text

	457	[sub7d_d_2]<br><br>Show the field ONLY if:<br>[sub7d_d] = '1'	If your organization were to conduct habitat or landscape management tick control, would it target private properties?	yesno, Required<br><table><tr><td>1</td><td>Yes</td></tr><tr><td>0</td><td>No</td></tr></table>
	458	[sub7d_d_3]<br><br>Show the field ONLY if:<br>[sub7d_d] = '0'	Why is your organization uninterested in conducting habitat or landscape management tick control?[select all that apply]	checkbox, Required<br><table><tr><td>1</td><td>sub7d_d_3___1</td><td>ticks and tickborne disease are not a priority concern in our service area</td></tr><tr><td>2</td><td>sub7d_d_3___2</td><td>concerns for safety of staff</td></tr><tr><td>3</td><td>sub7d_d_3___3</td><td>environmental concerns</td></tr><tr><td>4</td><td>sub7d_d_3___4</td><td>lack of interest from contracting agency</td></tr><tr><td>5</td><td>sub7d_d_3___5</td><td>not profitable</td></tr><tr><td>6</td><td>sub7d_d_3___6</td><td>other</td></tr></table>
	459	[sub7d_d_3_a]<br><br>Show the field ONLY if:<br>[sub7d_d_3(6)] = '1'	Please describe any other reason(s) your organization is uninterested in conducting habitat or landscape management tick control:	text
	460	[subsection_7d_habitat_or_landscape_management_52f5_complete]	Section Header: *Form Status*<br><br>Complete?	dropdown<br><table><tr><td>0</td><td>Incomplete</td></tr><tr><td>1</td><td>Unverified</td></tr><tr><td>2</td><td>Complete</td></tr></table>
Instrument: **Subsection 7b: Deer targeted intervention** (subsection_7b_deer_targeted_intervention_d8a0) Enabled as survey				
	461	[sub7b_e]<br><br>Show the field ONLY if:<br>[sub7_f(2)] = '1'	Section Header: *Capacity of private operators contracted by government entities to deploy deer-targeted tick control*<br><br>What deer-targeted tick control methods does your organization deploy?[select all that apply]	checkbox, Required<br><table><tr><td>1</td><td>sub7b_e___1</td><td>deer removal</td></tr><tr><td>2</td><td>sub7b_e___2</td><td>deer reproductive control</td></tr><tr><td>3</td><td>sub7b_e___3</td><td>4-poster for topical application of acaricide to deer</td></tr><tr><td>4</td><td>sub7b_e___4</td><td>deer fencing</td></tr><tr><td>5</td><td>sub7b_e___5</td><td>planting plants unpalatable to deer</td></tr><tr><td>6</td><td>sub7b_e___6</td><td>other</td></tr></table>

<table>
<tr><td></td><td>462</td><td>[sub7b_i_1]

Show the field ONLY if:
[sub7b_e(6)] = '1'</td><td>Please describe any other deer-targeted tick control method(s) your organization deploys:</td><td>text</td></tr>
<tr><td></td><td>463</td><td>[sub7b_a]

Show the field ONLY if:
[sub7_f(2)] = '1'</td><td>Approximately what size is the area covered by your organization's deer-targeted tick control?</td><td>radio, Required<table>
<tr><td>1</td><td>0 - 1 acre (0 - 0.001 sq mi)</td></tr>
<tr><td>2</td><td>1 - 10 acres (0.001 - 0.016 sq mi)</td></tr>
<tr><td>3</td><td>10 - 100 acres (0.01 - 0.156 sq mi)</td></tr>
<tr><td>4</td><td>100 - 1000 acres (0.156 - 1.563 sq mi)</td></tr>
<tr><td>6</td><td>1,000 - 10,000 acres (1.563 - 15.625 sq mi)</td></tr>
<tr><td>7</td><td>> 10,000 acres (> 15.625 sq mi)</td></tr>
</table></td></tr>
<tr><td></td><td>464</td><td>[sub7b_b_1]

Show the field ONLY if:
[sub7_f(2)] = '1'</td><td>What type(s) of public property does your organization target with deer-targeted tick control?[select all that apply]</td><td>checkbox, Required<table>
<tr><td>1</td><td>sub7b_b_1___1</td><td>parks</td></tr>
<tr><td>2</td><td>sub7b_b_1___2</td><td>picnic areas</td></tr>
<tr><td>3</td><td>sub7b_b_1___3</td><td>community centers</td></tr>
<tr><td>4</td><td>sub7b_b_1___4</td><td>school grounds</td></tr>
<tr><td>5</td><td>sub7b_b_1___5</td><td>forested areas</td></tr>
<tr><td>6</td><td>sub7b_b_1___6</td><td>campgrounds</td></tr>
<tr><td>7</td><td>sub7b_b_1___7</td><td>summer camps</td></tr>
<tr><td>8</td><td>sub7b_b_1___8</td><td>golf course</td></tr>
<tr><td>9</td><td>sub7b_b_1___9</td><td>other</td></tr>
</table></td></tr>
<tr><td></td><td>465</td><td>[sub7b_b_1_a]

Show the field ONLY if:
[sub7b_b_1(9)] = '1'</td><td>Please describe any other type(s) of public property your organization targets with deer-targeted tick control:</td><td>text</td></tr>
<tr><td></td><td>466</td><td>[sub7b_b]

Show the field ONLY if:
[sub7_f(2)] = '1'</td><td>Does your organization target private property when deploying deer-targeted tick control?</td><td>yesno, Required<table>
<tr><td>1</td><td>Yes</td></tr>
<tr><td>0</td><td>No</td></tr>
</table></td></tr>
<tr><td></td><td>467</td><td>[sub7b_b_3]

Show the field ONLY if:
[sub7b_b] = '1'</td><td>Please briefly name or describe the deer-targeted methods deployed on private property:</td><td>notes</td></tr>
</table>

	468	[sub7b_b_2]<br><br>Show the field ONLY if:<br>[sub7b_b] = '1'	What triggers your organization to deploy deer-targeted tick control on private property?[select all that apply]	checkbox, Required<br>1 | sub7b_b_2___1 | request of property owners<br>2 | sub7b_b_2___2 | according to field surveillance data<br>3 | sub7b_b_2___3 | according to human case data<br>4 | sub7b_b_2___4 | other
	469	[sub7b_b_2_a]<br><br>Show the field ONLY if:<br>[sub7b_b_2(4)] = '1'	Please describe any other reason(s) that trigger your organization to deploy deer-targeted tick control on private property:	notes
	470	[sub7b_c]<br><br>Show the field ONLY if:<br>[sub7_f(2)] = '1'	How does your organization evaluate the efficacy of deer-targeted tick control?[select all that apply]	checkbox, Required<br>1 | sub7b_c___1 | reduction in host-seeking ticks<br>2 | sub7b_c___2 | reduction in ticks submitted by the public<br>3 | sub7b_c___3 | reduction in public complaints or service requests<br>4 | sub7b_c___4 | reduction in human tickborne disease cases<br>5 | sub7b_c___5 | other evaluation metric<br>6 | sub7b_c___6 | no evaluation
	471	[sub7b_c_1]<br><br>Show the field ONLY if:<br>[sub7b_c(5)] = '1'	Please describe any other way(s) that your organization evaluates the efficacy of deer-targeted tick control:	text
	472	[sub7b_d]<br><br>Show the field ONLY if:<br>[sub7_f(2)] = '0'	If training opportunities were available, and you were asked by your contracting agency, would your organization be interested in developing the capacity to deploy deer-targeted tick control?	yesno, Required<br>1 | Yes<br>0 | No

	473	[sub7b_d_1]<br><br>Show the field ONLY if:<br>[sub7b_d] = '1'	If your organization were to deploy deer-targeted tick control, what type(s) of public property would be most feasible for your operation to target?[select all that apply]	checkbox, Required<br>1 | sub7b_d_1___1 | parks<br>2 | sub7b_d_1___2 | picnic areas<br>3 | sub7b_d_1___3 | community centers<br>4 | sub7b_d_1___4 | school grounds<br>5 | sub7b_d_1___5 | forested areas<br>6 | sub7b_d_1___6 | campgrounds<br>7 | sub7b_d_1___7 | summer camps<br>8 | sub7b_d_1___8 | golf course<br>9 | sub7b_d_1___9 | other
	474	[sub7b_d_1_a]<br><br>Show the field ONLY if:<br>[sub7b_d_1(9)] = '1'	Please describe any other type(s) of public property your organization might target for deer-targeted tick control:	text
	475	[sub7b_d_2]<br><br>Show the field ONLY if:<br>[sub7b_d] = '1'	If your organization were to deploy deer-targeted tick control, would it treat private properties?	yesno, Required<br>1 | Yes<br>0 | No
	476	[sub7b_d_3]<br><br>Show the field ONLY if:<br>[sub7b_d] = '0'	Why is your organization uninterested in deploying deer-targeted tick control?[select all that apply]	checkbox, Required<br>1 | sub7b_d_3___1 | ticks and tickborne disease are not a priority concern in our service area<br>2 | sub7b_d_3___2 | concerns for safety of staff<br>3 | sub7b_d_3___3 | environmental concerns<br>4 | sub7b_d_3___4 | lack of interest from contracting agency<br>5 | sub7b_d_3___5 | lack of trained personnel<br>6 | sub7b_d_3___6 | not profitable<br>7 | sub7b_d_3___7 | other

	477	[sub7b_d_3_a]<br>Show the field ONLY if:<br>[sub7b_d_3(7)] = '1'	Please describe any other reason(s) your organization is uninterested in deploying deer-targeted tick control:	text
	478	[subsection_7b_deer_targeted_intervention_d8a0_complete]	Section Header: *Form Status*<br>Complete?	dropdown<br>0 Incomplete<br>1 Unverified<br>2 Complete
Instrument: **Subsection 8: Overall tick management program services** (subsection_8_overall_tick_management_program_servi)<br>Enabled as survey				
	479	[sub8_a]	Section Header: *Overall capacity of agencies to deploy tick control*<br>If funding and resources were available to develop a comprehensive tick management program in your agency, what services would you offer? [select all that apply]	checkbox, Required<br>1 sub8_a___1 tick surveillance to identify and inform community of local areas with high risk of tick and tickborne disease exposure<br>2 sub8_a___2 control of tick populations on private properties (e.g., acaricide application, deer-targeted intervention, rodent-targeted intervention)<br>3 sub8_a___3 control of tick populations on public properties or public natural lands (e.g., acaricide application, deer-targeted intervention, rodent-targeted intervention)<br>4 sub8_a___4 guidance for residential property owners or public property managers conducting or contracting for tick control on their property<br>5 sub8_a___5 tick control efficacy assessments for residential property owners or public property managers and pest control firms<br>6 sub8_a___6 community outreach regarding tick-bite prevention and tick control methods

	480	[sub8_b_matrix]<br>Show the field ONLY if:<br>[sub8_a(1)] = '1' or [sub8_a(2)] = '1' or [sub8_a(3)] = '1' or [sub8_a(4)] = '1' or [sub8_a(5)] = '1' or [sub8_a(6)] = '1'	Rank the priority (1 = highest / 6 = lowest) of the services selected above for your agency's tick management program:	descriptive
	481	[sub8_b_matrix_a]<br>Show the field ONLY if:<br>[sub8_a(1)] = '1'	tick surveillance to identify and inform community of local areas with high risk of tick and tickborne disease exposure	radio (Matrix - ranking), Required<br>1 1<br>2 2<br>3 3<br>4 4<br>5 5<br>6 6
	482	[sub8_b_matrix_b]<br>Show the field ONLY if:<br>[sub8_a(2)] = '1'	control of tick populations on private properties (e.g., acaricide application, deer-targeted intervention, rodent-targeted intervention)	radio (Matrix - ranking), Required<br>1 1<br>2 2<br>3 3<br>4 4<br>5 5<br>6 6
	483	[sub8_b_matrix_f]<br>Show the field ONLY if:<br>[sub8_a(3)] = '1'	control of tick populations on public properties or public natural lands (e.g., acaricide application, deer-targeted intervention, rodent-targeted intervention)	radio (Matrix - ranking), Required<br>1 1<br>2 2<br>3 3<br>4 4<br>5 5<br>6 6

	484	[sub8_b_matrix_c]<br><br>Show the field ONLY if:<br>[sub8_a(4)] = '1'	guidance for residential property owners or public property managers conducting or contracting for tick control on their property	radio (Matrix - ranking), Required<br>1 1<br>2 2<br>3 3<br>4 4<br>5 5<br>6 6
	485	[sub8_b_matrix_d]<br><br>Show the field ONLY if:<br>[sub8_a(5)] = '1'	tick control efficacy assessments for residential property owners or public property managers and pest control firms	radio (Matrix - ranking), Required<br>1 1<br>2 2<br>3 3<br>4 4<br>5 5<br>6 6
	486	[sub8_b_matrix_e]<br><br>Show the field ONLY if:<br>[sub8_a(6)] = '1'	community outreach regarding tick-bite prevention and tick control methods	radio (Matrix - ranking), Required<br>1 1<br>2 2<br>3 3<br>4 4<br>5 5<br>6 6
	487	[sub8_c_matrix]<br><br>Show the field ONLY if:<br>[sub8_a(1)] = '1' or [sub8_a(2)] = '1' or [sub8_a(3)] = '1' or [sub 8_a(4)] = '1' or [sub8_a(5)] = '1' or [sub8_a(6)] = '1'	If your agency were to offer each of the services mentioned in the previous question individually, how much of an annual increase do you estimate your organization's budget would need for each service, considering increases in personnel or equipment or the need to reorganize existing staff / equipment?	descriptive

	488	[sub8_c_matrix_a]<br>Show the field ONLY if:<br>[sub8_a(1)] = '1'	tick surveillance to identify and inform community of local areas with high risk of tick and tickborne disease exposure	radio (Matrix), Required<br>1 0%<br>2 1-25%<br>3 25-50%<br>4 50-75%<br>5 75-100%<br>6 more than 100%
	489	[sub8_c_matrix_b]<br>Show the field ONLY if:<br>[sub8_a(2)] = '1'	control of tick populations on private properties (e.g., acaricide application, deer-targeted intervention, rodent-targeted intervention)	radio (Matrix), Required<br>1 0%<br>2 1-25%<br>3 25-50%<br>4 50-75%<br>5 75-100%<br>6 more than 100%
	490	[sub8_c_matrix_f]<br>Show the field ONLY if:<br>[sub8_a(3)] = '1'	control of tick populations on public properties or public natural lands (e.g., acaricide application, deer-targeted intervention, rodent-targeted intervention)	radio (Matrix), Required<br>1 0%<br>2 1-25%<br>3 25-50%<br>4 50-75%<br>5 75-100%<br>6 more than 100%
	491	[sub8_c_matrix_c]<br>Show the field ONLY if:<br>[sub8_a(4)] = '1'	guidance for residential property owners or public property managers conducting or contracting for tick control on their property	radio (Matrix), Required<br>1 0%<br>2 1-25%<br>3 25-50%<br>4 50-75%<br>5 75-100%<br>6 more than 100%

	492	[sub8_c_matrix_d]<br>Show the field ONLY if:<br>[sub8_a(5)] = '1'	tick control efficacy assessments for residential property owners or public property managers and pest control firms	radio (Matrix), Required<br>1 0%<br>2 1-25%<br>3 25-50%<br>4 50-75%<br>5 75-100%<br>6 more than 100%
	493	[sub8_c_matrix_e]<br>Show the field ONLY if:<br>[sub8_a(6)] = '1'	community outreach regarding tick-bite prevention and tick control methods	radio (Matrix), Required<br>1 0%<br>2 1-25%<br>3 25-50%<br>4 50-75%<br>5 75-100%<br>6 more than 100%
	494	[sub8_d]	Do you believe your community would support a tax increase to initiate or expand a comprehensive tick management program?	yesno, Required<br>1 Yes<br>0 No
	495	[sub8_d_y1]<br>Show the field ONLY if:<br>[sub8_d] = '1'	By what percentage would current taxes or fees devoted to vector management have to be increased in order to achieve a sustainable program?	slider (number), Required<br>Slider labels: 0%, 50%, 100%<br>Custom alignment: RH
	496	[sub8_e_header]	If training in tick control operations and specific tick control methods were provided by CDC or professional organizations (e.g., ESA, AMCA), please rank the preferred training vehicles from the selection below.<br><br>Please rank below choices in order of preference, with most preferred as '1' and least preferred as '6':	descriptive

	497	[sub8_e1]	webinars	radio (Matrix - ranking), Required<br>1 1<br>2 2<br>3 3<br>4 4<br>5 5<br>6 6
	498	[sub8_e2]	in-person courses including hands-on training	radio (Matrix - ranking), Required<br>1 1<br>2 2<br>3 3<br>4 4<br>5 5<br>6 6
	499	[sub8_e3]	in-person courses without hands-on training	radio (Matrix - ranking), Required<br>1 1<br>2 2<br>3 3<br>4 4<br>5 5<br>6 6
	500	[sub8_e4]	digital materials	radio (Matrix - ranking), Required<br>1 1<br>2 2<br>3 3<br>4 4<br>5 5<br>6 6

	501	[sub8_e5]	mailers	radio (Matrix - ranking), Required<br>1 | 1<br>2 | 2<br>3 | 3<br>4 | 4<br>5 | 5<br>6 | 6
	502	[sub8_e6]	pre-recorded videos	radio (Matrix - ranking), Required<br>1 | 1<br>2 | 2<br>3 | 3<br>4 | 4<br>5 | 5<br>6 | 6
	503	[sub8_e_other]	Please describe any other optimal tick control training vehicle(s):	notes
	504	[sub8_f]	If funding and resources were available, would your agency be interested and capable of testing new or alternative tick control methods?	yesno, Required<br>1 | Yes<br>0 | No<br><br>Custom alignment: RH
	505	[subsection_8_overall_tick_management_program_servi_complete]	Section Header: *Form Status*<br>Complete?	dropdown<br>0 | Incomplete<br>1 | Unverified<br>2 | Complete
